# Non-functional ubiquitin C-terminal hydrolase L1 drives podocyte injury through impairing proteasomes in autoimmune glomerulonephritis

**DOI:** 10.1038/s41467-023-37836-8

**Published:** 2023-04-13

**Authors:** Julia Reichelt, Wiebke Sachs, Sarah Frömbling, Julia Fehlert, Maja Studencka-Turski, Anna Betz, Desiree Loreth, Lukas Blume, Susanne Witt, Sandra Pohl, Johannes Brand, Maire Czesla, Jan Knop, Bogdan I. Florea, Stephanie Zielinski, Marlies Sachs, Elion Hoxha, Irm Hermans-Borgmeyer, Gunther Zahner, Thorsten Wiech, Elke Krüger, Catherine Meyer-Schwesinger

**Affiliations:** 1 Institute of Cellular and Integrative Physiology, Center for Experimental Medicine, University Medical Center Hamburg-Eppendorf, Hamburg, Germany; 2 Institute of Medical Biochemistry and Molecular Biology, University Medicine Greifswald, Hamburg, Germany; 3 Protein production Core Facility, Centre for Structural Systems Biology, Deutsches Elektronen-Synchrotron DESY, Hamburg, Germany; 4 Skeletal Pathobiochemistry, Department of Osteology and Biomechanics, Center for Experimental Medicine, University Medical Center Hamburg-Eppendorf, Hamburg, Germany; 5 Bio-organic synthesis group, Leiden University, Leiden, The Netherlands; 6 III Medical Clinic and Polyclinic, Nephrology, University Medical Center Hamburg-Eppendorf, Hamburg, Germany; 7 Transgenic Animal Service Group, Center for Molecular Neurobiology Hamburg, University Medical Center Hamburg-Eppendorf, Hamburg, Germany; 8 Institute of Pathology, Nephropathology Section, University Medical Center Hamburg-Eppendorf, Hamburg, Germany

**Keywords:** Membranous nephropathy, Mechanisms of disease, Deubiquitylating enzymes

## Abstract

Little is known about the mechanistic significance of the ubiquitin proteasome system (UPS) in a kidney autoimmune environment. In membranous nephropathy (MN), autoantibodies target podocytes of the glomerular filter resulting in proteinuria. Converging biochemical, structural, mouse pathomechanistic, and clinical information we report that the deubiquitinase Ubiquitin C-terminal hydrolase L1 (UCH-L1) is induced by oxidative stress in podocytes and is directly involved in proteasome substrate accumulation. Mechanistically, this toxic gain-of-function is mediated by non-functional UCH-L1, which interacts with and thereby impairs proteasomes. In experimental MN, UCH-L1 becomes non-functional and MN patients with poor outcome exhibit autoantibodies with preferential reactivity to non-functional UCH-L1. Podocyte-specific deletion of UCH-L1 protects from experimental MN, whereas overexpression of non-functional UCH-L1 impairs podocyte proteostasis and drives injury in mice. In conclusion, the UPS is pathomechanistically linked to podocyte disease by aberrant proteasomal interactions of non-functional UCH-L1.

## Abstract (web summary)

In membranous nephropathy autoantibodies target podocytes of the kidney filter resulting in injury. Here the authors show that the ensuing proteostatic disturbances and proteinuria relate to aberrant interactions of non-functional UCH-L1 enzyme with the proteasome, curtailing its capacity.

## Introduction

There is an unmet need for quantitative and mechanistic research into the role of the ubiquitin proteasome system (UPS) in human pathophysiology^1^, especially in kidney disease. As a major quality control system responsible for degradation of proteins, the UPS comprises a cascade of E1-E2-E3 enzymes, which ubiquitinate substrate proteins and hence target them for proteasomal degradation. Deubiquitinating enzymes (DUBs) edit ubiquitin chains from substrate proteins and thereby modify their cellular fate^2^. The proteasome, as a complex protease, exists in multiple structural isoforms but contains two general assemblies: a proteolytic chamber (20S core particle) harboring the proteolytical active β subunits, and a regulatory particle (19S particle) which are functionally linked by a gated protein translocation channel to form the 26S/30S proteasome^3^. Protein degradation through the 20S core particle is achieved in an ATP and ubiquitin independent manner, whereas ATP and ubiquitin are required for degradation through the 26S/30S proteasome^2^.

An induction of the UPS in conjunction with an accumulation of (K48-) polyubiquitinated proteins^3,4^ is one hallmark of membranous nephropathy (MN) and indicative of progressive podocyte injury^3,4^. MN is an autoimmune disease of the kidney, which targets podocytes, the key cells of the glomerular filter^5^. These terminally differentiated epithelial cells embrace the glomerular capillaries with their intricate net of interdigitating foot processes. Together with endothelial cells and the glomerular basement membrane, podocytes form the three-layered size and charge selective filtration barrier to blood. Nephrotic syndrome is the clinical hallmark of podocytopathies such as MN and presents with massive proteinuria, hypoalbuminemia, edema, and hyperlipidemia. Besides diabetes and focal segmental glomerulosclerosis (FSGS), MN is the most frequent cause of nephrotic range protein loss to the urine in adults^6,7^. MN is initiated by circulating autoantibodies directed to the podocyte foot process antigens such as PLA_2_R1^8^ and THSD7A^9^. The ensuing pathomechanistic sequelae of this disease is unclear. Besides the subepithelial glomerular deposition of immune complexes and complement factors, the occurrence of oxidative stress^10,11^ within podocytes are common findings.

In 2009 we^12^ and others^13,14^ described a de novo expression of Ubiquitin C-terminal hydrolase L1 (UCH-L1) in injured podocytes in human and rodents, with MN being one of the prominent glomerulonephritis forms^12^. The role of UCH-L1 in podocyte injury remains enigmatic. As a central neuronal DUB, with unknown direct substrates, UCH-L1 is thought to stabilize the pool of monoubiquitin in neurons^15^, a finding however not reproduced in a new murine model of UCH-L1 deficiency in the brain^16^ or kidney^17^. Point mutations of UCH-L1 have been identified in human neurodegenerative disorders, such as the I93M, C90S, and the E7A^18^ mutations, which all result in a decreased hydrolysis activity of the enzyme^19^. The I93M mutation additionally gives rise to a dominant toxic gain-of-function variant. While the overall crystal structure of the wildtype and the I93M variant is very similar^20^, comparative biophysical analysis show an apparent loss of α-helical structure in solution, hence decreasing solubility of the I93M variant^21,22^. In the brain, UCH-L1 is thought to promote anti-oxidative properties^23^ and to be itself modified by oxidative stress^24^. Oxidatively-modified UCH-L1 has been shown to acquire comparable structural and biochemical properties as the I93M-UCH-L1, including a decreased hydrolysis function and an increased tendency to insolubility and abnormal interactions^22,25^ for example with Lamp2^26^ and tubulin^22^.

This report unravels the role of UCH-L1 in podocyte injury by integrating experimental evidence from 1) biopsies and sera from well-defined MN patient cohorts, 2) podocyte culture and mouse models of experimental MN, 3) genetically modified mice with podocyte-specific UCH-L1-deletion compared to overexpression of functional versus non-functional (I93M) UCH-L1, and 4) mechanistic studies on proteasome function. Our data demonstrate that UCH-L1 is not only induced by oxidative stress in podocytes, but also presents as an enzymatic non-functional protein in experimental MN. Non-functional (but not functional) UCH-L1 drives podocyte injury and ubiquitin accumulation in experimental MN, mechanistically by binding to the proteasome and reducing constitutive proteasomal subunit activities. MN patients exhibit autoantibodies to UCH-L1 with a differential reactivity to non-functional UCH-L1, an indirect indication that UCH-L1 modification might occur in humans.

## Results

### Oxidative and proteotoxic stress is induced in a UCH-L1-dependent manner in MN

**Fig. 1:**
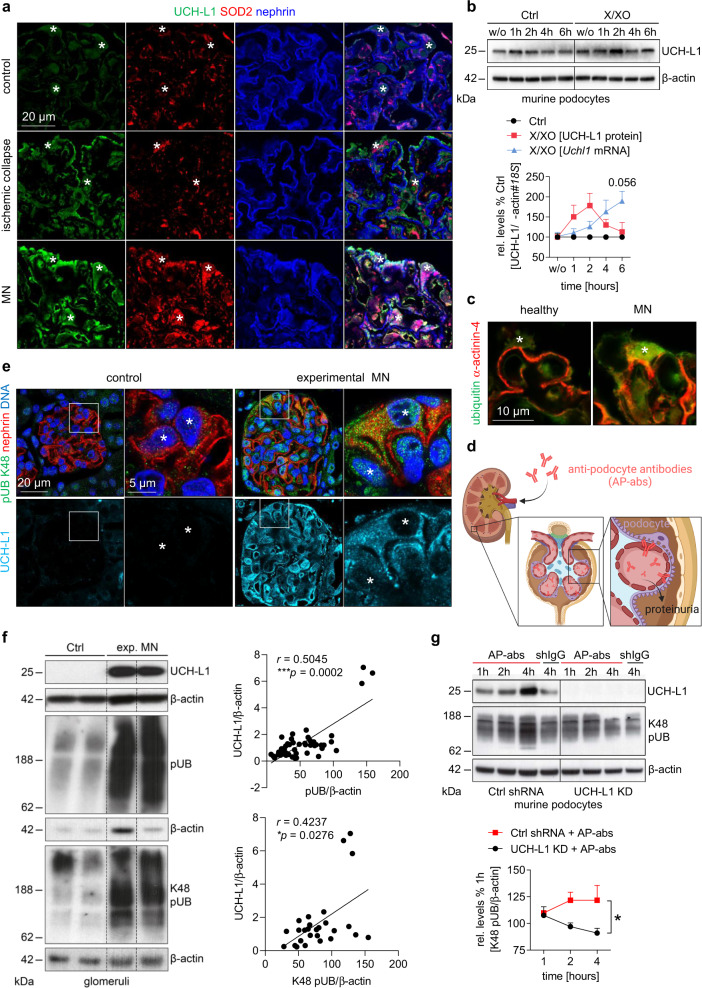
Oxidative stress leads to UCH-L1 upregulation, which induces ubiquitin accumulations in injured podocytes. **a** Confocal analyses of 2 independent experiments with *n* = 3 exhibiting de novo UCH-L1 expression (green) correlating with enhanced SOD2 scavenger protein levels (red) in podocytes (asterisks) in renal patient biopsies with ischemic glomerular collapse or membranous nephropathy (MN). Nephrin (blue) demarcates the glomerular filtration barrier. **b** To induce oxidative stress podocytes were treated without (w/o) as well as for 1, 2, 4 and 6 hours with 150 µM xanthine (X) and 200 mU xanthine oxidase (XO) or equal volume of NaOH and K_2_HPO_4_ (Ctrl). Relative UCH-L1 protein (red line) and transcript (blue line) abundance in comparison to respective controls (Ctrl, black line) assessed by immunoblot normalized to β-actin or via qPCR normalized to *18* *S*. Pooled values of 4 independent experiments with *n* = 1, mean + /-SEM, Two-Way ANOVA with Geisser-Greenhouse correction. **c** Confocal images of 2 independent experiments with *n* = 3 illustrate ubiquitinated protein accumulations (green) in aggrieved podocytes (asterisk) in a renal MN biopsy. α-actinin-4 (red) marks podocytes. **d** Mouse model of experimental MN is induced by injection of sheep anti-podocyte antibodies (AP-abs), which lead to podocyte injury and proteinuria by binding podocyte foot-process antigens (created with BioRender.com). **e** Experimental MN was initiated by i.v. injection of AP-abs or sheep IgG (shIgG, control) in mice. Kidneys were collected on day 14. Confocal images of 2 independent experiments with *n* = 3 show K48-polyubiquitinated protein aggregation (green) in UCH-L1-expressing podocytes (turquoise). Asterisks = podocyte nuclei, nephrin (red), DNA (Hoechst, blue). **f** Glomerular immunoblot quantification of UCH-L1 and (K48-) polyubiquitinated proteins (pUB) normalized to β-actin. Graphs show Spearman´s correlation (two-tailed) between UCH-L1 and (K48-) polyubiquitinated proteins. Pooled values of 4 (K48 pUB) or 6 (pUB) independent experiments with *n* = 27 (K48 pUB) or *n* = 49 (pUB). **g** ShRNA-mediated knockdown (KD) of UCH-L1 in podocytes prevents K48-polyubiquitinated protein accumulation after exposure to AP-abs for 1, 2 and 4 hours contrary to scrambled shRNA transduced podocytes (Ctrl shRNA). Pooled values of 3 independent experiments with *n* = 1, mean + /-SEM, *p = 0.0301, Two-Way ANOVA with Geisser-Greenhouse correction. Source data are provided as a Source Data file.

We established whether oxidative stress, a prominent feature of MN, induces UCH-L1 in podocytes. In biopsy samples of patients diagnosed with ischemic glomerular collapse and MN, podocyte UCH-L1 immunoreactivity correlated with an enhanced expression of superoxide dismutase 2 (SOD2), a mitochondrial scavenger of reactive oxygen species (ROS) (Fig. 1a). Mouse podocytes exposed to oxidative stress through incubation with xanthine oxidase (which catalyzes the oxidation of xanthine to uric acid and thereby generates ROS), exhibited an upregulation of UCH-L1 protein and transcript. UCH-L1 protein peaked after the first 2 hours of ROS exposure, whereas the increase of UCH-L1 transcript followed by a lag of 2-4 hours (Fig. 1b). The early peak of UCH-L1 protein pointed to a protein stabilization effect caused by impaired protein homeostasis (proteostasis) (Supplementary Fig. 1) rather than an upregulation of expression. The timing of UCH-L1 transcript increase exhibited a comparable time course to other ROS-detoxifying transcripts (Supplementary Fig. 2a–c) and scavenging of ROS diminished UCH-L1 expression (Supplementary Fig. 2d, e), suggesting that UCH-L1 was part of an anti-oxidative stress response in podocytes.

Besides oxidative stress, the accumulation of ubiquitin is a prominent feature of podocyte injury in MN^4^ (Fig. 1c). We therefore assessed whether UCH-L1 expression correlated with ubiquitin accumulation in a mouse model of experimental MN induced by sheep anti-podocyte antibodies^27^ (scheme Fig. 1d). These anti-podocyte antibodies exhibit a polyclonal reactivity to podocyte proteins^27^, including to human and murine UCH-L1 protein (Supplementary Fig. 3). Comparable to human MN, UCH-L1 was de novo induced in podocytes in this model (Supplementary Fig. 4). UCH-L1-expressing podocytes exhibited abundant K48-polyubiquitin immunoreactivity in the cytoplasm, major processes, and nuclei (Fig. 1e). Immunoblot quantification of glomerular UCH-L1 and ubiquitin protein abundance demonstrated a significant correlation of both polyubiquitin and K48-polyubiquitin levels to respective UCH-L1 levels in experimental MN (Fig. 1f). Importantly, UCH-L1-deficiency obtained via shRNA-mediated knockdown in mouse podocytes prevented the accumulation of K48-polyubiquitin after exposure to sheep anti-podocyte antibodies (Fig. 1g). Together, these results suggest that UCH-L1 is upregulated upon oxidative stress in podocytes and is directly involved in the accumulation of ubiquitylated proteasome substrates, therefore potentially promoting podocyte injury through impairment of proteostasis. We therefore assessed whether the podocyte expression of UCH-L1 protein at the time of diagnosis correlated with an unfavorable disease outcome in MN. Typically, 1/3 of MN patients go into spontaneous remission, whereas 1/3 progress to end-stage kidney injury. To this end, our analyses confirmed that UCH-L1 protein was present in podocytes of patients with MN but revealed no correlation of UCH-L1 expression with later disease outcome (Supplementary Fig. 5). This finding led us to hypothesize that not a functional but rather a non-functional (i.e., oxidative-modified) UCH-L1 enzyme perturbs podocyte proteostasis.

### Induced UCH-L1 is non-functional in experimental MN and MN patients exhibit autoantibodies to non-functional UCH-L1

**Fig. 2:**
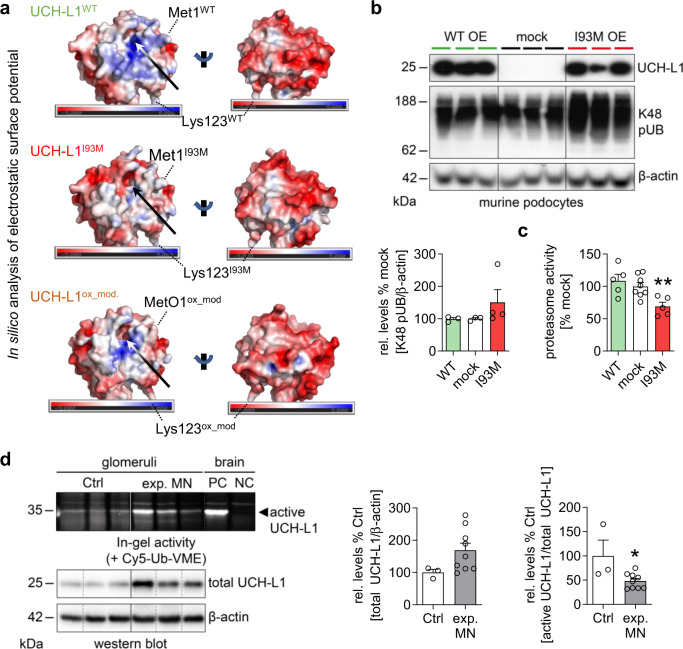
UCH-L1 is modified in experimental MN. **a** Calculated electrostatic surface potential of the crystal structures of UCH-L1 wildtype (PDB: 2ETL, UCH-L1^WT^), and its I93M (PDB:3IRT, UCH-L1^I93M^) variant and of the predicted crystal structure of an oxidative-modified variant (UCH-L1^ox_mod^) in two orientations in surface representation. Surface is colored according to the electrostatic surface potential, using a ramp from −5kT/e to +5kT/e at which the surface colors are clamped at red for a negative or blue for a positive electrostatic potential, visualized by a slider. Location of the amino acid residues Lys123 and Met1 in UCH-L1^WT^ and UCH-L1^I93M^ or methionine sulfoxide MetO1 in UCH-L1^ox_mod^ are indicated. While the surface of the active site entrance (arrows) in the oxidative-modified variant is less positively charged compared to the wildtype (upper panel), the dorsal side of the oxidative-modified variant shows positive surface charge which is missing in the wildtype (lower panel). **b**, **c** Doxycycline-induced overexpression of I93M-UCH-L1 or WT-UCH-L1 in murine podocytes. **b** K48 pUB levels were determined by immunoblot and normalized to β-actin, *n* = 3 (WT&mock) or *n* = 4 (I93M) of 2 independent experiments, mean + /-SEM, two-tailed Mann Whitney *U* test. **c** Examination of the main proteasomal chymotrypsin-like activity by measurement of Suc-LLVY-AMC peptide hydrolysis, *n* = 5 (WT&I93M) or *n* = 8 (mock) of 2 independent experiments, mean + /-SEM, **p = 0.0062, two-tailed Mann Whitney *U* test. **d** Experimental MN was initiated by i.v. injection of AP-abs or sheep IgG (Ctrl) in mice. Kidneys were collected on day 14. Glomerular enzymatic in-gel activity of UCH-L1 was measured using a ubiquitin-based activity probe (Cy5-Ub-VME), which irreversibly binds to the catalytic center of active deubiquitinating enzymes. Brain lysates of *Uchl1*^*-/-*^ mice were used as negative control (NC), of *Uchl1*^*+/+*^ mice as positive control (PC). Levels of active UCH-L1 were quantified by measuring in-gel fluorescence of labeled probe at 35 kDa subsequently normalized to respective total UCH-L1 protein levels (normalized to β-actin of the same membrane). Values are presented as mean + /-SEM, *n* = 3 (Ctrl) or *n* = 9 (exp. MN) of 1 experiment, *p = 0.0364, two-tailed Mann Whitney *U* test. Source data are provided as a Source Data file.

Oxidative stress results in UCH-L1 modification at multiple amino acids, which differ within the human and the mouse protein depending on the experimental systems analyzed^22,24,25,28^. Oxidative-modified UCH-L1 and its I93M variant are thought to have comparable biochemical and structural properties promoting a toxic gain-of-function in neurons^22^. In line, in silico analysis of the electrostatic surface potential calculated using the same constraints and ranges are overall similar between wildtype (WT)-UCH-L1 and modified UCH-L1 proteins. However, both, the I93M mutant and the ROS-modified UCH-L1, are less positively charged around the entrance to the active site compared to the (WT)-UCH-L1 based on identified oxidative-modified amino acids of human UCH-L1^24^. Further, the dorsal side of the oxidative-modified variant shows positive surface charge which is missing in the wildtype (Fig. 2a). We therefore used the I93M variant as a surrogate for “non-functional” UCH-L1 to determine its effect on podocyte proteostasis in comparison to “functional” WT-UCH-L1. Indeed, the doxycycline-induced overexpression of I93M-UCH-L1, but not of WT-UCH-L1, in mouse podocytes impaired proteostasis with an accumulation of K48-polyubiquitinated proteins (Fig. 2b) and a reduction of proteasome chymotrypsin-like activity (proteolysis of the substrate Suc-LLVY-AMC; Fig. 2c).

To assess whether UCH-L1 is rendered non-functional during experimental MN, we made use of the fact that non-functional UCH-L1 exhibits a reduction in accessible active sites when quantified using a fluorescent-tagged activity-based probe (Cy5-Ub-VME). This probe covalently binds to the catalytic center of only active DUBs, including UCH-L1 (Supplementary Fig. 6). In experimental MN the amount of enzymatically active UCH-L1 was strongly decreased in glomeruli, although total UCH-L1 levels were increased (ratio of active [fluorescent] to total [immunoblot] UCH-L1; Fig. 2d). This strongly suggests the presence of high amounts of non-functional UCH-L1 enzyme in experimental MN.

**Fig. 3:**
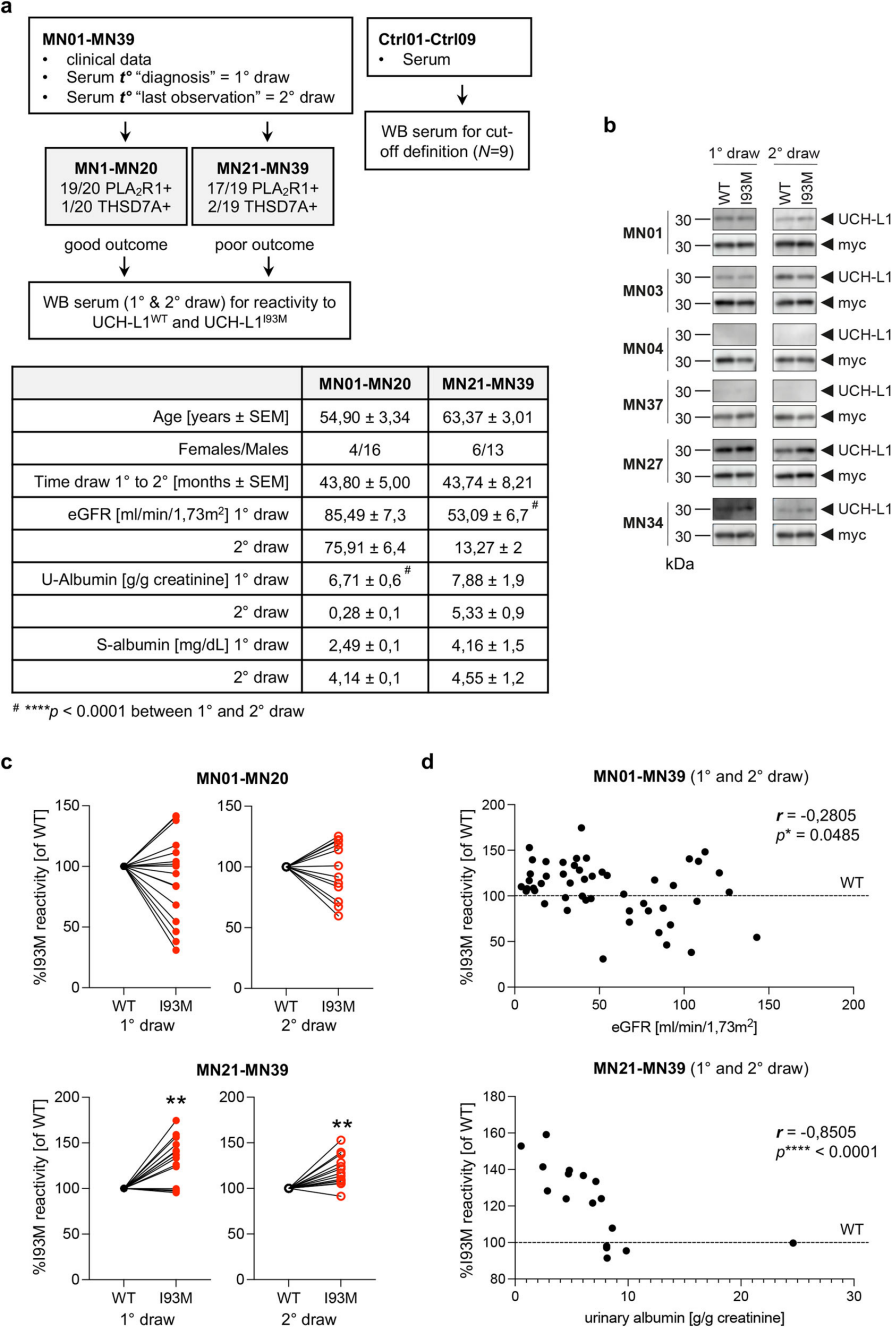
Patients with nephrotic syndrome exhibit autoantibodies with preferential affinity to non-functional UCH-L1 protein. **a** Characteristics of the membranous nephropathy patient cohort analyzed; ***t°*** = timepoint, MN = membranous nephropathy, WB = Western blot. The table summarizes clinical baseline characteristics of the cohort; ****p < 0.0001 between the first and second serum draw within one group, One Way ANOVA with Tukey’s multiple comparisons test. **b** Selected WB of patient sera diluted 1:50 to purified human UCH-L1^WT^ and UCH-L1^I93M^ protein and corresponding myc detection of the same membrane to control for UCH-L1 protein loading. MN01 & MN03 depict an equal/reduced reactivity to UCH-L1^I93M^ compared to UCH-L1^WT^; MN04 & MN37 are sero-negative for anti-UCH-L1 antibodies; MN27 & MN34 exhibit an enhanced reactivity to UCH-L1^I93M^ compared to UCH-L1^WT^. **c** Individual sera reactivity to UCH-L1^I93M^ was calculated in comparison to reactivity to UCH-L1^WT^ (set at 100%) of the same membrane. Preferential reactivity to UCH-L1^I93M^ was observed in MN patients with poor outcome at the 1° and 2° draw; values from *n* = 16 (MN01-MN20) or *n* = 14 (MN21-MN39) patients, **p = 0.0052 (1° draw), **p = 0.0012 (2° draw), two-tailed Wilcoxon test. **d** Correlation of % reactivity to UCH-L1^I93M^ in comparison to UCH-L1^WT^ (100%, dotted line) at the time of 1° and 2° draw to clinical parameters of the time-matched sera. Reactivity to UCH-L1^I93M^ inversely correlates to eGFR in MN patients (upper graph) and to urinary albumin abundance in MN patients with poor outcome (lower graph); Spearman´s correlation (two-tailed). Source data are provided as a Source Data file.

Whether non-functional UCH-L1 exists in humans, specifically whether the injury-related podocyte-expressed UCH-L1 in MN patients is non-functional technically cannot be assessed in archived tissue using the activity-based probe. Therefore, we set out to search for indirect evidence for the existence of non-functional UCH-L1 in MN patients. As autoantibodies to UCH-L1 have been identified in a subset of sera of patients with MN^29^, we evaluated the immunoreactivity of sera collected in a well-defined MN patient cohort (Fig. 3a) to non-functional I93M-UCH-L1 (as a surrogate of oxidative-modified UCH-L1) and functional UCH-L1. 39 MN patients were randomized based on eGFR, urinary protein loss, and serum creatinine values at time of diagnosis and at time of their last observation into a group with “good outcome” (MN01-MN20) and into a group with “poor outcome” (MN21-MN39) (Fig. 3a, table). To this end, 78 MN sera from the time of diagnosis and from the last observation timepoint and 9 healthy control sera (for definition of cut-off for serum negativity/positivity to UCH-L1) were analyzed by immunoblot for their reactivity towards flag-purified human WT-UCH-L1 and I93M-UCH-L1 protein (Fig. 3b). MN patients exhibited autoantibodies to UCH-L1 in 30/39 sera of the first draw and in 26/39 sera of the second draw, 9/39 MN patients were sero-negative for UCH-L1 at both serum draws. The binding reactivity to UCH-L1 protein varied between MN sera (Fig. 3b). When normalized to UCH-L1 protein loading, sero-positive sera exhibited a preferential reactivity to WT-UCH-L1 (i.e., MN01, MN03) or a stronger reactivity to I93M-UCH-L1 (i.e., MN27, MN34). In 38 of the 56 UCH-L1 autoantibody-positive sera the reactivity was significantly higher to I93M-UCH-L1 than to the WT-UCH-L1. Correlative analyses demonstrated that most (but not all) of the sera with preferential reactivity to I93M-UCH-L1 grouped to MN patients with “poor outcome” (Fig. 3c). Further, a negative correlation of MN sera with preferential reactivity to I93M-UCH-L1 was present to eGFR (Fig. 3d, upper graph), as well as a negative correlation to urinary albumin abundance in the MN group with “poor outcome” (Fig. 3d, lower graph). The detection of autoantibodies with a preferential affinity to I93M-UCH-L1 in MN patients suggests that modification of UCH-L1 might occur in humans. Whether these UCH-L1 autoantibodies are related to MN or to other unidentified patient conditions requires further investigations. Of note, neither a causative relationship between UCH-L1 autoantibody levels and disease state, nor a pathophysiologic significance of UCH-L1 autoantibodies in MN can be claimed based on the presented analyses.

Taken together, our investigations suggest that non-functional UCH-L1 protein is present in injured podocytes in experimental MN and that autoantibodies with enhanced binding affinity to non-functional UCH-L1 are present in a subset of MN patients.

### Transgenic overexpression of non-functional UCH-L1 induces podocyte ubiquitin accumulations

**Fig. 4:**
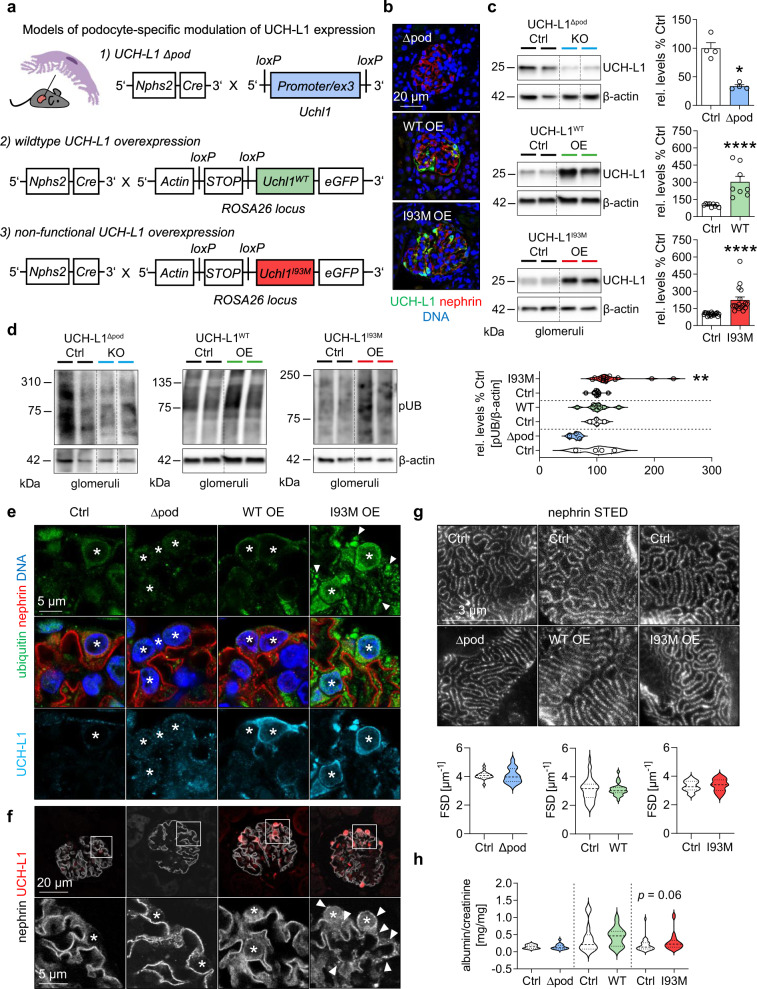
Transgenic overexpression of non-functional I93M-UCH-L1 protein leads to ubiquitin aggregation in podocytes. **a** Scheme of the transgenic mouse models of modified UCH-L1 expression in podocytes (created with BioRender.com). Naive mice were subsequently analyzed: **b** Confocal images from 2 independent experiments with *n* = 3 verifies podocyte UCH-L1 knockout/overexpression (green). Nephrin (red) depicts the glomerular filtration barrier, DNA (Hoechst, blue). **c** Glomerular immunoblot quantification of UCH-L1. Graph shows relative UCH-L1 abundance (normalized to β-actin) to respective littermate controls (Ctrl). Pooled values of 7 independent experiments, mean + /-SEM, *n* = 4 Ctrl or Δpod, *n* ≤ 9 WT (*n* = 9 Ctrl, *n* = 8 WT OE) or *n* ≤ 19 I93M (*n* = 19 Ctrl, *n* = 17 I93M OE), *p = 0.0286, ****p < 0.0001, two-tailed Mann Whitney *U* test. The faint UCH-L1 band in UCH-L1Δpod, arises from glomerular endothelial UCH-L1 expression. **d** Immunoblot quantification of glomerular polyubiquitinated proteins (pUB). Graph shows relative ubiquitin abundance (normalized to β-actin) to respective littermate controls (Ctrl). Pooled values of 7 independent experiments, mean + /-SEM, *n* ≤ 4 Δpod (*n* = 4 Ctrl, *n* = 3 Δpod), *n* ≤ 8 WT (*n* = 4 Ctrl, *n* = 8 WT OE) or *n* ≤ 15 I93M (*n* = 11 Ctrl, *n* = 15 I93M OE), **p = 0.003, two-tailed Mann Whitney *U* test. **e** Representative staining from 2 independent experiments with *n* = 3 of ubiquitinated proteins (green) in relation to UCH-L1 expression (turquoise), nephrin (red), DNA (Hoechst, blue), asterisks = podocyte nuclei, arrowheads = granular ubiquitin accumulations. **f** Confocal analyses of 2 independent experiments with *n* = 3 for nephrin (white) and UCH-L1 (red) demonstrates granular nephrin pattern (arrowheads) in UCH-L1^I93M^ overexpressing podocytes, asterisks = podocytes. **g** STED analyses of nephrin meanders to quantify filtration slit density (FSD) length per area (µm^−1^). Graphs depict pooled values of 2 independent experiments, mean + /-SEM, *n* ≤ 19 Δpod (*n* = 19 Ctrl, *n* = 14 Δpod), *n* ≤ 21 WT (*n* = 15 Ctrl, *n* = 21 WT OE) or *n* ≤ 31 I93M (*n* = 31 Ctrl, *n* = 23 I93M OE), two-tailed Unpaired *t*-test. **h** Urinary albumin to creatinine ratio. Pooled values of 15 independent experiments, mean + /-SEM, *n* ≤ 17 Δpod (*n* = 13 Ctrl, *n* = 17 Δpod), *n* ≤ 20 WT (*n* = 14 Ctrl, *n* = 20 WT OE) or *n* ≤ 30 I93M (*n* = 30 Ctrl, *n* = 25 I93M OE), two-tailed Mann Whitney *U* test. Source data are provided as a Source Data file.

To unravel the pathomechanistic significance of non-functional UCH-L1 for podocyte proteostasis and MN disease development, three mouse models with podocyte-specific modulation of UCH-L1 expression were generated (Fig. 4a–c, Supplemental Fig. 7): mice with UCH-L1-deletion (UCH-L1^Δpod^, 1), mice with overexpression of functional wildtype UCH-L1 (UCH-L1^WT^, 2), and mice with non-functional I93M-UCH-L1 (UCH-L1^I93M^, 3). Glomerular UCH-L1 protein abundance was comparable in both overexpressing transgenic mouse lines (UCH-L1^WT^ with a mean of 303,311% ±137,39 and UCH-L1^I93M^ with a mean of 223,045% ±111,94). Consistent with podocyte culture data (Fig. 2b, c), the expression of I93M-UCH-L1 but not of WT-UCH-L1 increased glomerular ubiquitin levels in vivo (Fig. 4d). Immunohistologically, these glomerular ubiquitin accumulations were restricted to UCH-L1^I93M^ podocytes, which exhibited an enhanced and granular staining for ubiquitin (Fig. 4e, Supplementary Fig. 8) as well as for the slit membrane protein nephrin (Fig. 4f). Like ubiquitin, nephrin levels also increased in UCH-L1^I93M^ glomeruli (Supplementary Fig. 9). We next determined whether modulated UCH-L1 expression resulted in a functional alteration of podocytes. Foot process slit membrane density (FSD) measurements using STED microscopy to visualize the nephrin meanders showed a comparable FSD between UCH-L1^Δpod^, UCH-L1^WT^, UCH-L1^I93M^ and their controls (Fig. 4g). Functionally, no gross alterations of filtration barrier function were visible, as no significant albuminuria to respective controls was present in the three genetic models, although UCH-L1^I93M^ mice exhibited a slightly increased leakiness to albumin (Fig. 4h). In summary, we successfully generated mouse models of altered podocyte-specific UCH-L1 modulation, in which only the expression of non-functional UCH-L1 results in a slight leakiness of the glomerular filtration barrier and to a granular accumulation of ubiquitin and nephrin in podocytes.

### Podocyte-specific deletion of UCH-L1 attenuates experimental MN

We next asked, whether genetic modulation of UCH-L1 in podocytes affected the course of experimental MN, hypothesizing that the presence of non-functional UCH-L1 should aggravate podocyte injury in the sense of a toxic gain-of-function.

**Fig. 5:**
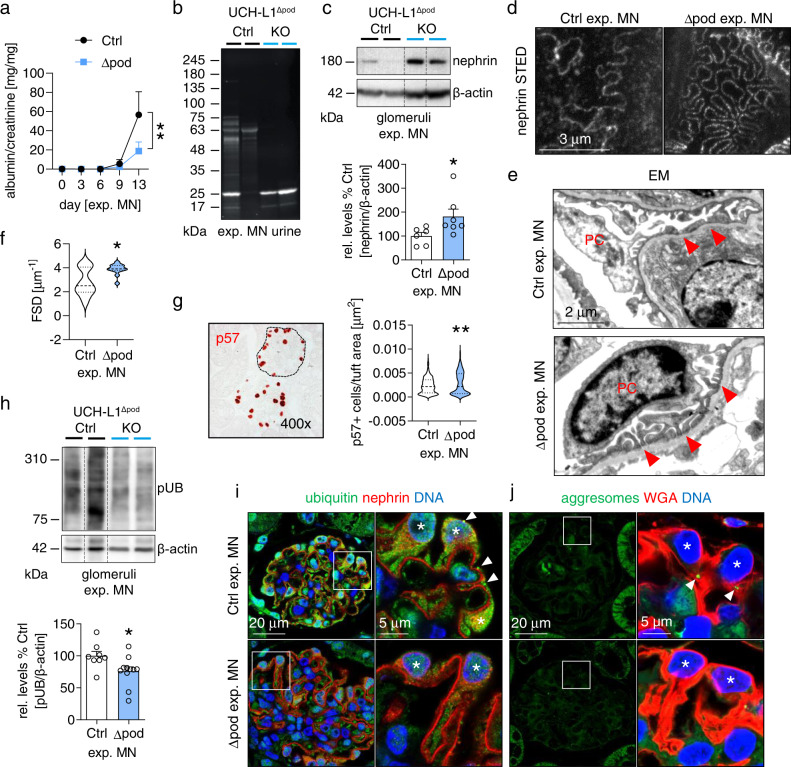
Podocyte-specific UCH-L1 deletion attenuates ubiquitin accumulations and podocyte injury in experimental MN. Experimental MN was induced by i.v. injection of AP-abs in UCH-L1-deficient (Δpod) and littermate (Ctrl) mice. Kidneys were collected on day 14. **a** Urinary albumin to creatinine ratio. Pooled values of 6 independent experiments, mean + /-SEM, per time *n* ≤ 15 (Ctrl) or *n* ≤ 18 (Δpod), **p = 0.0022, Two-Way ANOVA. **b** Stainfree SDS-PAGE from 1 experiment of creatinine-adapted urines depicts loss of high molecular weight proteins in Ctrl mice, albumin ~63 kDa. **c** Immunoblot quantification of glomerular nephrin abundance normalized to β-actin. Pooled values of 2 independent experiments, mean + /-SEM, *n* = 6 (Ctrl) or *n* = 7 (Δpod), *p = 0.014, two-tailed Mann Whitney *U* test. **d** STED analyses of nephrin meanders. **e** Electron microscopy (EM) demonstrates effaced foot processes in Ctrl and mostly preserved foot processes in Δpod mice, PC = podocyte, red arrowheads = foot processes. **f** Quantification of foot process effacement by PEMP analyses of filtration slit density (FSD) length per area (µm^−1^), pooled values of 1 experiment, mean + /-SEM, *n* = 15 (Ctrl) or *n* = 12 (Δpod), *p = 0.0321, two-tailed Mann Whitney *U* test. **g** Podocyte number per glomerular tuft area assessed by staining for p57 (podocyte marker). Pooled values of 3 independent experiments, mean + /-SEM, *n* = 271 (Ctrl) or *n* = 332 (Δpod) glomeruli, **p = 0.0014, two-tailed Unpaired *t*-test. **h** Glomerular immunoblots for polyubiquitinated proteins (pUB) normalized to β-actin. Pooled values of 3 independent experiments, mean + /-SEM, *n* = 8 (Ctrl) or *n* = 11 (Δpod), *p = 0.0328, two-tailed Mann Whitney *U* test. **i** Representative confocal images of 2 independent experiments with *n* = 3 showing enhanced signal for ubiquitinated proteins (green) in Ctrl podocytes with occasional ubiquitin aggregates (arrowheads). Nephrin (red) was used to depict the glomerular filtration barrier and DNA was stained with Hoechst (blue). Podocytes are marked by asterisks. **j** Representative confocal images of 2 independent experiments with *n* = 3 showing inclusion bodies (aggresomes, marked by arrowheads) in Ctrl podocytes. Wheat germ agglutinin (WGA, red) was used to highlight the podocyte plasma membrane. DNA was stained with Hoechst (blue). Asterisks demarcate podocytes. Source data are provided as a Source Data file.

UCH-L1^Δpod^ and controls were exposed to sheep anti-podocyte antibodies to induce experimental MN and functional and structural parameters were evaluated on day 14. To this end, albuminuria and loss of high molecular weight proteins were attenuated in UCH-L1^Δpod^ mice demonstrating decreased disruption of the filtration barrier in the absence of UCH-L1 (Fig. 5a, b). In line, the glomerular abundance of nephrin was enhanced in UCH-L1^Δpod^ in comparison to controls (Fig. 5c). This increased nephrin abundance was mirrored by mostly preserved nephrin meanders by STED microscopy and of mostly preserved foot process ultrastructure by electron microscopical examinations in UCH-L1^Δpod^ mice (Fig. 5d, e, Supplementary Fig. 10). Quantification of FSD was significant for foot process effacement in controls, whereas FSD was preserved in UCH-L1^Δpod^ mice (Fig. 5f). Of note, not only FSD was preserved, but also podocyte count per glomerular tuft area was higher in UCH-L1^Δpod^ mice (Fig. 5g), demonstrating a decreased loss of podocytes during experimental MN in UCH-L1^Δpod^ mice. Immunoblot quantification of glomerular polyubiquitin abundance demonstrated significantly decreased levels in UCH-L1^Δpod^ compared to controls (Fig. 5h). Confocal microscopy showed that the decrease in overall glomerular ubiquitin levels was most likely attributable to a lesser abundance of ubiquitin in podocytes of UCH-L1^Δpod^ mice (Fig. 5i). Aggresome formation, a sign of misfolded protein aggregation occurring when cellular protein degradation systems are overwhelmed, was present in control but not in UCH-L1^Δpod^ mice (Fig. 5j).

### Transgenic overexpression of non-functional UCH-L1 aggravates podocyte injury in experimental MN

**Fig. 6:**
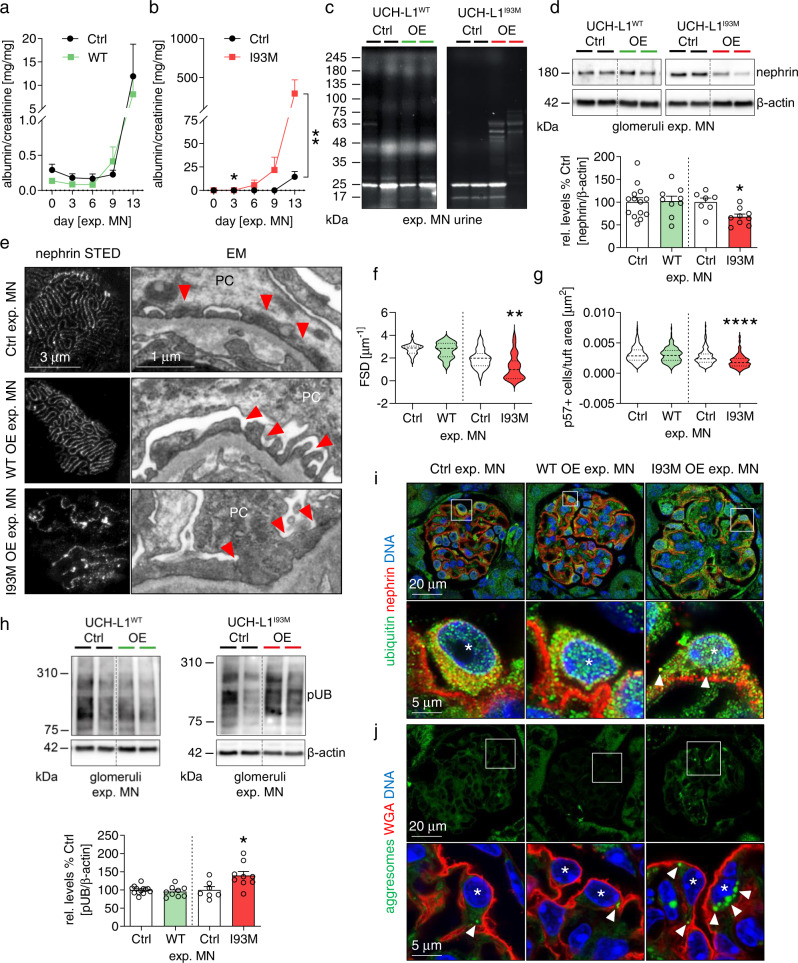
Overexpression of non-functional I93M-UCH-L1 perpetuates podocyte injury and ubiquitin accumulations in experimental MN. Experimental MN was induced in UCH-L1 overexpressing (WT OE and I93M OE) and respective littermate (Ctrl) mice. Kidneys were collected on day 14. **a**, **b** Urinary albumin to creatinine ratio, pooled values of 5 independent experiments (WT-UCH-L1, *n* ≤ 18 Ctrl or *n* ≤ 15 OE, per time) or of 2 independent experiments (I93M-UCH-L1, *n* ≤ 9 Ctrl or *n* = 10 OE, per time), mean + /-SEM, *p = 0.0434, **p = 0.0062, two-tailed Mann Whitney *U* test. **c** Stainfree SDS-PAGE from 1 experiment of creatinine-adapted urines depicts loss of high molecular weight proteins in I93M OE mice, albumin ~63 kDa. **d** Glomerular quantification of relative nephrin abundance (normalized to β-actin) to Ctrl. Pooled values of 4 independent experiments (WT-UCH-L1, *n* = 14 Ctrl or *n* = 9 WT OE) or of 3 independent experiments (I93M-UCH-L1, *n* = 7 Ctrl or *n* = 9 I93M OE), mean + /-SEM, *p = 0.0115, two-tailed Mann Whitney *U* test. **e** Left: STED analyses of nephrin meanders. Right: electron microscopy (EM) of glomerular filtration barrier integrity, arrowheads = foot processes, PC = podocyte. **f** Quantification of foot process effacement by PEMP analyses of filtration slit density (FSD) length per area (µm^−1^). Pooled values of 1 experiment (WT-UCH-L1, *n* = 24 Ctrl or *n* = 36 WT OE; I93M-UCH-L1, *n* = 23 Ctrl or *n* = 35 I93M OE), mean + /-SEM, **p = 0.0041, two-tailed Mann Whitney *U* test. **g** Quantification of podocyte loss by counting p57+ podocytes per glomerular tuft area. Pooled values of 2 independent experiments (WT-UCH-L1, *n* = 350 Ctrl or *n* = 198 WT OE) or of 1 experiment (I93M-UCH-L1, *n* = 150 Ctrl or *n* = 128 I93M OE) as mean + /-SEM, ****p < 0.0001, two-tailed Unpaired *t*-test. **h** Quantification of glomerular polyubiquitinated (pUB) proteins normalized to β-actin relative to Ctrl. Pooled values of 4 independent experiments (WT-UCH-L1, *n* = 13 Ctrl or *n* = 9 WT OE) or of 2 independent experiments (I93M-UCH-L1, *n* = 7 Ctrl or *n* = 9 I93M OE) are shown as mean + /-SEM, *p = 0.0164, two-tailed Mann Whitney *U* test. Confocal images from 2 independent experiments with *n* = 3 of (**i**) ubiquitinated proteins (green) in podocytes. Arrowheads = ubiquitin aggregates, nephrin (red) depicts the glomerular filtration barrier or of (**j**) inclusion bodies, aggresomes = arrowheads), wheat germ agglutinin (WGA, red) highlights the podocyte plasma membrane. Podocytes = asterisks, DNA (Hoechst, blue). Source data are provided as a Source Data file.

Loss of UCH-L1 expression protected podocytes from experimental MN. We therefore assessed the effects of WT-UCH-L1 and I93M-UCH-L1 expression for the functional and structural podocyte integrity in experimental MN on day 14. To this end the expression of non-functional UCH-L1 resulted in an aggravation of proteinuria compared to controls in experimental MN (Fig. 6a, b) and to an enhanced appearance of high molecular weight proteins in the urine (Fig. 6c). This was accompanied by a significant decrease of nephrin abundance in UCH-L1^I93M^ glomeruli (Fig. 6d). Morphological examinations were substantial for a severe broadening and disruption of nephrin meanders by STED microscopy (Fig. 6e left panel), severe foot process effacement by electron microscopical examinations (Fig. 6e right panel, Supplementary Fig. 11), and for a significant decrease of FSD in UCH-L1^I93M^ mice compared to controls (Fig. 6f). Of note UCH-L1^WT^ mice exhibited similar structural and functional podocyte alterations as their controls. Podocyte loss was severe in UCH-L1^I93M^ and was not observed in UCH-L1^WT^ mice compared to controls (Fig. 6g). Assessment of polyubiquitin levels showed an increased abundance of ubiquitin conjugates in UCH-L1^I93M^ glomeruli, whereas polyubiquitin levels were comparable between UCH-L1^WT^ and controls (Fig. 6h). Confocal microscopy demonstrated that the enhanced glomerular ubiquitin levels were mostly restricted to podocytes in UCH-L1^I93M^ mice (Fig. 6i). Precisely, podocytes of UCH-L1^I93M^ mice were hypertrophied and ubiquitin signal appeared more granular than in UCH-L1^WT^ and respective controls. Strikingly, podocytes of UCH-L1^I93M^ mice exhibited prominent aggresomes indicating severe proteotoxic stress in comparison to UCH-L1^WT^ mice and controls (Fig. 6j). Additional studies in the newly developed THSD7A-associated mouse model of MN^30^ mirrored the observed podocyte-injury promoting phenotype of non-functional UCH-L1 in affected podocytes (Supplementary Fig. 12). Together, these investigations suggest that ubiquitin-dependent protein degradation was strongly impaired in I93M-UCH-L1 but not in WT-UCH-L1 overexpressing podocytes, resulting in exacerbated podocyte injury.

### UCH-L1 interacts with the proteasome and non-functional UCH-L1 reduces proteasomal activity

**Fig. 7:**
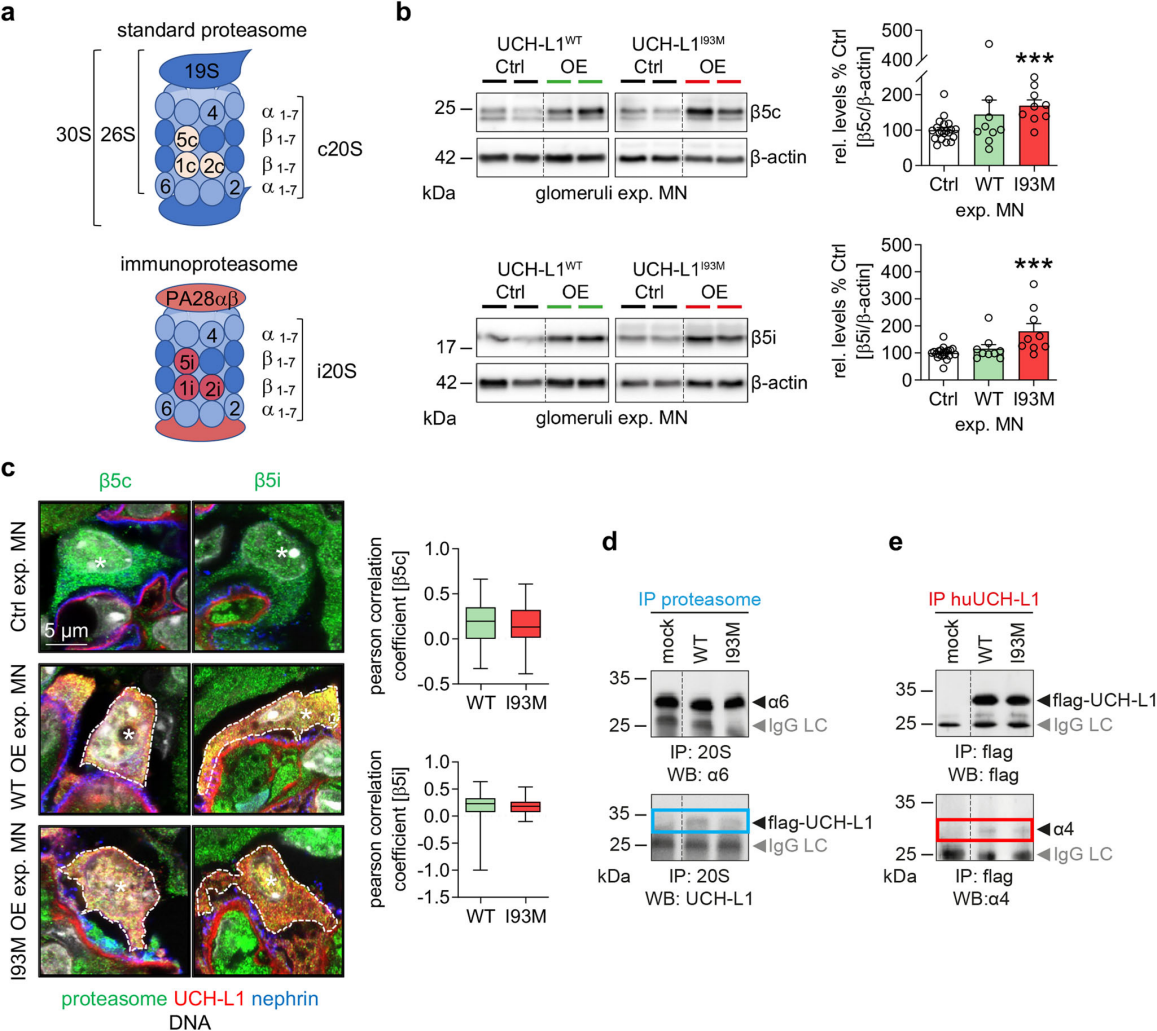
UCH-L1 interacts with the proteasome. **a** Scheme of the standard proteasome (proteolytic subunits β1c, β2c and β5c) and of the immunoproteasome (proteolytic subunits β1i, β2i and β5i). **b**, **c** Experimental MN was induced by i.v. injection of sheep anti-murine podocyte antibodies (AP-abs) in UCH-L1 overexpressing (WT OE and I93M OE, respectively) and their littermate (Ctrl) mice. Kidneys were collected on day 14. **b** Glomerular immunoblot analyses of main (β5c and β5i) proteasomal subunit abundance normalized to β-actin. Pooled values of 4 independent experiments (WT-UCH-L1, *n* = 20 Ctrl or *n* = 9 WT OE) or of 2 independent experiments (I93M-UCH-L1, *n* = 20 Ctrl or *n* = 9 I93M OE) for each subunit are shown as mean + /-SEM, ***p = 0.0004 (β5c), ***p = 0.0002 (β5i), two-tailed Mann Whitney *U* test. **c** Confocal images showing co-localization of respective proteolytic proteins (green) with UCH-L1 (red) in podocytes (marked by asterisks). Nephrin (blue) was used to depict the glomerular filtration barrier and DNA was stained with Hoechst (grey). Graphs: semiquantitative analysis of spatial proximity of β5c and β5i with UCH-L1 in selected podocytes (yellow overlay in framed area), respectively; strength of positive (0 to +1) or negative (0 to −1) linear relationship represented by Pearson correlation coefficient. The box illustrates the interquartile range (IQR), median line and whiskers including the lowest and highest values within ±1.5 × IQR and above. Values consist of *n* = 3 (WT-UCH-L1 or I93M-UCH-L1), two-tailed Unpaired *t*-test. **d**, **e** Interaction studies of UCH-L1 with the proteasome by using HEK293T cells transiently transfected with human (hu) wildtype UCH-L1-flag (WT-UCH-L1) and hu-I93M-UCH-L1-flag (I93M-UCH-L1) protein. Representative immunoblot analyses of 1 experiment demonstrate interaction between UCH-L1 and the proteasome. WT-UCH-L1 and non-functional I93M-UCH-L1 protein were co-immunoprecipitated with the 20S proteasome (**d**, lower panel). Successful proteasome IP is shown via immunoblot to the α6 subunit (**d**, upper panel). Vice versa the proteasome (detected via the α4 subunit, (**e**, lower panel)) was co-immunoprecipitated with UCH-L1 proteins, respectively (**e**, upper panel). Untransfected HEK293T cells served as control. IgG LC = immunoglobulin G light chain of the capture antibody. Source data are provided as a Source Data file.

**Fig. 8:**
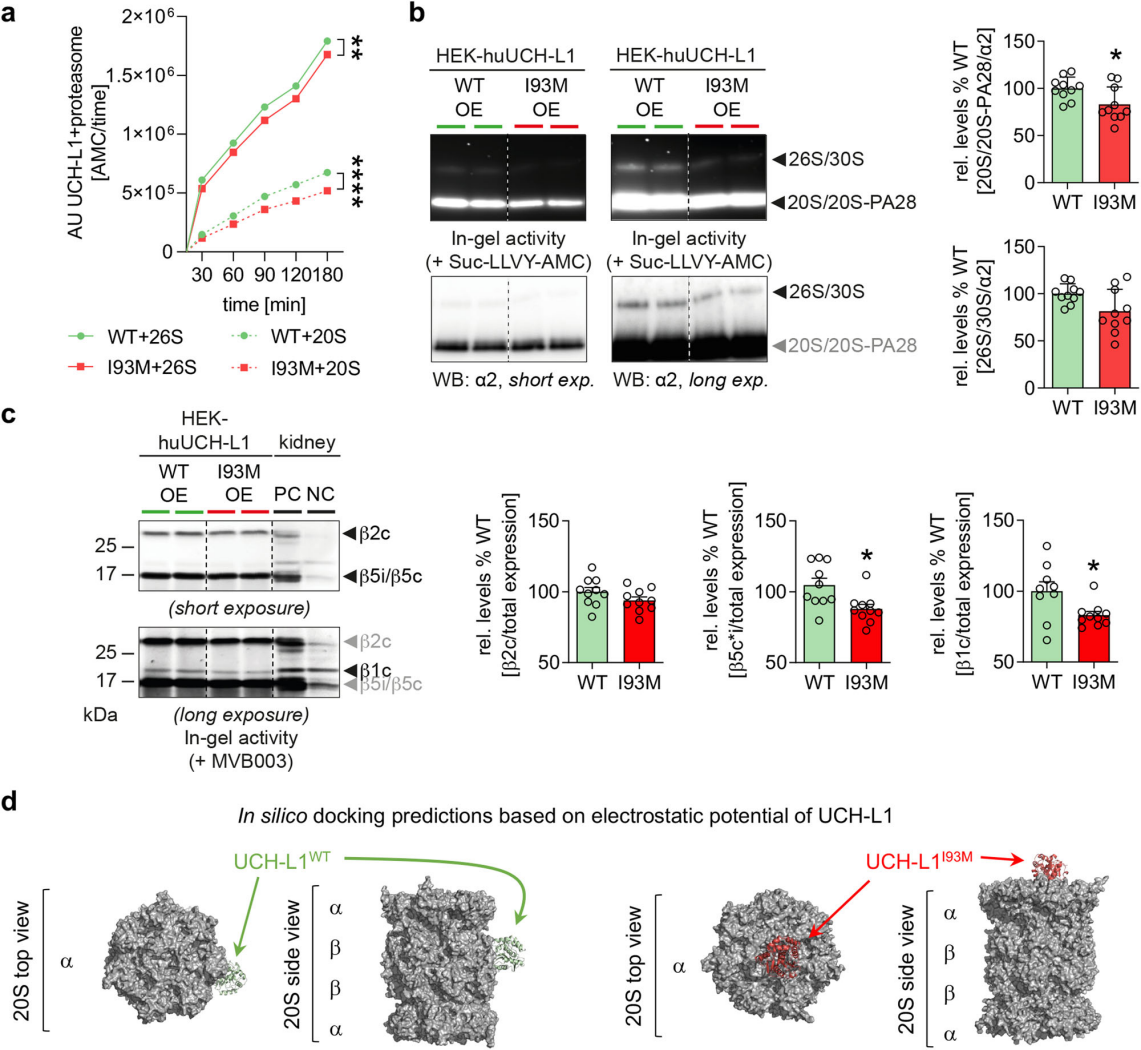
Non-functional protein curtails proteasomal capacity. **a** Time course of in vitro chymotrypsin-like activity of 10 µg purified UCH-L1-flag constructs incubated with 5 nM purified human (hu) 20S and 26S proteasome. Fluorescence intensities of converted proteasomal substrate Suc-LLVY-AMC (60 µM) per time were determined at 355 nm and 460 nm. Values are expressed as mean + /-SEM, *n* = 3 (WT-UCH-L1 or I93M-UCH-L1) per subunit, **p = 0.0057, ****p < 0.0001, Two-Way ANOVA with Geisser-Greenhouse correction. **b** Proteasomal in-gel activity of transfected HEK293T cells overexpressing human (hu) WT-UCH-L1 and I93M-UCH-L1 constructs by measuring the turnover of the fluorogenic β5c/β5i substrate Suc-LLVY-AMC (100 µM). In-gel activity was normalized to relative proteasome abundance of the same gel assessed by subsequent immunoblot against the α2 subunit of the 20S core particle. Pooled values of 4 independent experiments, *n* = 10, mean + /-SEM, *p = 0.0355, two-tailed Mann Whitney *U* test. **c** Subunit-specific proteasome activity was measured in-gel using 0,5 µM of the pan-proteasomal activity based-probe MVB003 in UCH-L1 overexpressing HEK293T cells. Total kidney lysates of BALB/c wildtype mice treated with 2 µM DMSO served as positive control (PC), treatment with 2 µM epoxomicin as negative control (NC). Constitutive β1c, β2c, and β5c subunit activities were normalized to total protein abundance of associated β subunits acquired from subsequent immunoblot of the activity gel. Pooled values of 3 independent experiments, *n* = 10 per construct (β2c or β5c*i) and *n* = 9 (WT-UCH-L1) or *n* = 10 (I93M-UCH-L1) for β1c, mean + /-SEM, *p = 0.0107 (β5c*i), *p = 0.0258 (β1c), two-tailed Unpaired *t*-test. **d** Selected representative binding configuration of hu-WT-UCH-L1 and hu-I93M variant to the human 20S proteasome as calculated using ClusPro (https://cluspro.bu.edu). Shown are top views and side views of the 20S proteasome (PDB 5LE5) in surface representation in grey and the exemplary configurations of UCH-L1 molecules in cartoon representation shown in green (WT-UCH-L1) bound to proteasomal β subunit and in red (I93M-UCH-L1) bound to a proteasomal α subunit. The collection of all calculated biologically relevant in silico docking predictions are shown in the Supplementary Fig. 17. Source data are provided as a Source Data file.

Non-functional UCH-L1 is present in experimental MN and drives podocyte injury through impairment of proteostasis, suggesting the presence of impaired proteasomal degradation potentially due to a toxic gain-of-function of non-functional UCH-L1. We first established total levels of standard and immunoproteasome in glomeruli of mutant mice on day 14 of experimental MN by immunoblot quantification of their respective main proteolytic subunits β5c and β5i (Fig. 7a, b). In comparison to controls, UCH-L1^I93M^ but not UCH-L1^WT^ mice exhibited a significantly increased abundance of both β subunits. Colocalization studies suggested a spatial proximity of UCH-L1 with both β subunits in UCH-L1^I93M^ and UCH-L1^WT^ podocytes in experimental MN (Fig. 7c, Supplementary Fig. 13). We next established that UCH-L1 binds to the proteasome using HEK293T cells expressing hu-WT-UCH-L1 or hu-I93M-UCH-L1 (Supplementary Fig. 14). To this end, both hu-I93M-UCH-L1 as well as hu-WT-UCH-L1, were co-immunoprecipitated with the proteasome (Fig. 7d), and vice versa proteasome subunits such as α4 were co-immunoprecipitated with both UCH-L1 constructs (Fig. 7e). No difference in proteasomal binding was observed between hu-WT-UCH-L1 or hu-I93M-UCH-L1. Fractionation of proteasome subtypes by glycerol gradient demonstrated, that both UCH-L1 proteins preferably co-migrated with hu-26S/30S proteasomes. Hu-I93M-UCH-L1, however, also co-migrated with the 20S core proteasome fraction (Supplementary Fig. 15). Since proteasome abundance was increased in UCH-L1^I93M^ mice, but ubiquitin accumulations and aggresomes were enhanced, we investigated whether I93M-UCH-L1 influenced proteasomal activity. The in vitro incubation of recombinant hu-20S or hu-26S proteasome with purified hu-I93M-UCH-L1 protein resulted in a decreased chymotrypsin-like activity when compared to proteasomes incubated with hu-WT-UCH-L1 protein (Fig. 8a). Of note, the activity of the hu-20S proteasome was affected strongest by hu-I93M-UCH-L1. This finding might be of pathophysiologic relevance, as the 20S proteasome degrades proteins in a ubiquitin and ATP-independent manner and is thought to exist especially in situations of oxidative stress^31,32^. Native in-gel measurements of the main proteasomal chymotrypsin-like activity demonstrated a decreased Suc-LLVY-AMC peptide hydrolysis especially by the 20S and less by 26S/30S proteasomes in HEK293T cells transfected with hu-I93M-UCH-L1 in comparison to when hu-WT-UCH-L1 was present (Fig. 8b). As the presence of hu-I93M-UCH-L1 reduced 20S activity in vitro and in HEK293T cells, we analyzed whether we could detect inhibitory effects of hu-I93M-UCH-L1 on subunit-specific β1c, β2c and β5c-activities situated in the constitutive 20S proteasome (Fig. 8c). In-gel active site subunit accessibility measurements using the pan-proteasomal activity-based probe MVB003 exhibited a significant reduction of β1c and β5c-activities, but not of β2c by hu-I93M-UCH-L1 compared to hu-WT-UCH-L1 (Fig. 8c) when normalized to respective constitutive β subunit protein abundance by immunoblot (Supplementary Fig. 16). Looking at representative sets of lowest energy configurations for the docking of UCH-L1 variants to the human 20S proteasome, we found that for WT-UCH-L1 docking to the α- and β subunits was rather equally likely, while for I93M-UCH-L1 we found a 60:40 preponderance of docking to the α subunit (Fig. 8d, Supplementary Fig. 17). These results could indicate that binding of I93M-UCH-L1 to the 20S α subunit alters unhindered access of substrates to the pore in the α-rings, thereby inhibiting proteasomal activity.

Together these results demonstrate that UCH-L1, independently of its functionality, co-localizes and binds to the proteasome. Only non-functional UCH-L1 inhibits proteasome activity, especially of the 20S proteasome and its β1c and β5c subunits, potentially by binding to α subunits and thus preventing unhindered access of substrates to the α-ring pore. In injured podocytes, a substantial portion of UCH-L1 is non-functional and patients exhibit autoantibodies with preferential binding activity to non-functional UCH-L1. The “compensatory” upregulation of both the standard and immunoproteasome, paired with an enhanced ubiquitin and aggresome abundance in UCH-L1^I93M^ podocytes suggest that non-functional UCH-L1 mediates proteasomal inhibition in vivo and hence perpetuates podocyte injury and experimental MN.

## Discussion

In this study we converge biochemical, structural, mouse pathomechanistic, and clinical information to unravel the significance of the ubiquitin proteasome system in human MN. Our work is centered on the hypothesis that protein degradation through the UPS is a common modifier of MN progression, severity, and outcome. The factors leading to and indicating progressive podocyte injury are much sought for, especially for the guidance of clinical decisions if disease outcome varies as strongly as in MN (1/3 of patients remit, 1/3 progress to end-stage kidney disease). Independent of the clinical outcome, podocytes respond uniformly to injury with structural damage such as foot process effacement. An upregulation of important UPS players, however, occurs in progressive but not in transient podocyte injury^4^. As terminally differentiated cells, injured podocytes are prone to develop proteasome impairment^4^, indirectly visible through the accumulation of (K48-) polyubiquitinated proteins. The factors triggering—and—the nature of proteasome impairment in MN are unclear but could range from clogging of the proteasome through free ubiquitin chains^33^, to posttranslational^34^ or ROS-mediated modification of proteasomes^35^, as described in non-renal disorders. In the autoimmunologic environment of MN we now demonstrate proteasomal impairment and aggravated disease to arise from a toxic gain-of-function UCH-L1, as one of the strongest upregulated UPS proteins in injured podocytes^12–14^.

In the mouse kidney, a conditional deficiency of UCH-L1 influences ubiquitin and protein homeostasis, proteasome composition^17^, and the glomerular filtration rate^36^. In neurons, UCH-L1 is not only part of an antioxidant response^23^ but is itself modified by ROS, thus reducing its enzymatic activity, and making it prone to a toxic gain-of-function^22,24^. In line with this proposed neuronal function of UCH-L1, we can show that podocytes upregulate UCH-L1 in response to oxidative stress, which is prominent in experimental MN^10,11^. Upregulated UCH-L1 in experimental MN exhibited reduced enzymatic activity, strongly suggesting oxidative damage to occur during podocyte injury. We could now demonstrate that UCH-L1 promotes podocyte injury, as UCH-L1^Δpod^ mice were protected from experimental MN^27,37^. In line, earlier investigations in rat experimental MN had shown that chemical modulation of UCH-L1 enzymatic activity enhances glomerular proteasomal activity and ameliorates the clinical course of disease^38^. Our efforts to correlate UCH-L1 protein expression to the clinical course of MN patients were inconclusive, even though in a previous study UCH-L1 expression differentiated patients with remitting minimal change disease from those that progressed to focal segmental glomerulosclerosis (FSGS)^4^. Besides technical issues, the distinct pathogenesis of podocyte injury (MN versus minimal change disease and FSGS) could explain the difference between the studies, as podocytes exhibit no UPS alterations in minimal change disease, whereas podocytes in FSGS and MN patients do^4,12^. Nonetheless, these findings add to the idea, that the presence of non-functional UCH-L1 rather than of functional UCH-L1 might be pivotal in the promotion of podocyte injury.

The mechanisms by which UCH-L1 perpetuates podocyte injury are not understood. This is partly due to (1) the complex biochemical functions of UCH-L1 (ligase and hydrolase-dependent and hydrolase-independent effects^19^); (2) the lack of knowledge regarding specific UCH-L1 targets in podocytes and (3) multiple UCH-L1 protein modifications, which alter UCH-L1 function and subcellular localization^19^. To unravel the disease-promoting effect of UCH-L1 in podocytes we therefore generated transgenic mice which expressed the enzymatic functional UCH-L1 wildtype protein or non-functional I93M-UCH-L1 in podocytes. We expected that expression of functional UCH-L1 would negatively affect podocyte function through the initiation of dedifferentiation^12^, through the induction of necroptosis^39^, or through the altered degradation of podocyte proteins^40^. However, UCH-L1^WT^ mice showed no major difference in their naive phenotype or in the clinical course of experimental MN compared to controls. This discrepancy might be related to the fact that most mechanistic findings to UCH-L1 are based on cell culture observations^12,39,40^, and relate on the analyses of whole glomeruli, where constitutive UCH-L1 expression in endothelial cells^41^ could mask de novo injury-induced UCH-L1 effects in podocytes. A further explanation could be related to technical issues, as potentially the overall expression level of UCH-L1^WT^ might have been too low (albeit comparable to UCH-L1^I93M^). To avoid too high UCH-L1 levels, most experiments were performed in heterozygous mice resulting in a UCH-L1 expression in 30-80% of podocytes within individual glomeruli.

While functional UCH-L1 overexpression did not aggravate podocyte injury, UCH-L1^I93M^ mice exhibited naive and injury-related proteostasis alterations. A comparable cellular injury inducing effect of the I93M variant was described in mice with transgenic expression of I93M-UCH-L1 under the PDGF-B promotor, which resulted in loss of dopaminergic neurons^42^. We could attribute part of this toxic gain-of-function to the interaction of UCH-L1 with the proteasome, which in case of I93M-UCH-L1 resulted in a decrease of 20S more than 26S/30S proteasome activity, and of the constitutive proteolytic β subunit activities. Based on structural modeling proteasome impairment by I93M-UCH-L1 could be related to binding of I93M-UCH-L1 to α subunits of the 20S core particle, which are less accessible in the 26S/30S proteasome, an observation recently supported by the interaction of UCH-L1 with α4 in a tumor setting^43^. Even though conflicting opinions exist considering the effect of I93M mutation on the extent of structural UCH-L1 alterations, the biological effects of oxidative-modified and I93M-UCH-L1 are comparable^19^. Therefore, we propose that the toxic gain-of-function sequelae observed in naive and injured podocytes of UCH-L1^I93M^ mice are synonymous to the sequelae one would observe by expressing oxidatively-modified UCH-L1 in podocytes or which result during MN.

Autoantibodies directed to intracellular proteins such as UCH-L1 have been identified in MN patient sera^29^. However, UCH-L1 autoantibodies are not specific to MN^44–46^. The origin, clinical^45^, and pathophysiologic significance of these UCH-L1 autoantibodies in podocyte injury remains to be experimentally established. They could be mere by-standers or indicate the additional existence of other (non-renal) medical conditions such as for example tumors^45^, which are a frequent co-occurrence in patients with MN. However, besides the primary autoantibodies to PLA_2_R1 and THSD7A, the additional presence of autoantibodies to intracellular proteins is thought to enhance the autoimmune burden of podocytes in MN^47^. Why MN patients exhibit autoantibodies to UCH-L1 is at this state not only an enigma for UCH-L1, but also for the main MN antigens PLA_2_R1 and THSD7A. One could speculate that UCH-L1 autoantibody development is the result of podocyte-expressed (non-functional) UCH-L1 being presented to the immune system by an unidentified glomerular exit route such as extracellular vesicles, in which UCH-L1 is present^48,49^. However, UCH-L1 autoantibody development could also represent a by-standing result from another underlying medical condition. Nevertheless, as non-functional UCH-L1 drives podocyte injury in experimental MN, further investigations in larger cohorts are warranted to establish whether there is a translational significance for autoantibodies to non-functional UCH-L1 in patients with podocytopathies.

In summary, we provide evidence, that non-functional UCH-L1 is present in podocytopathies such as MN. In addition, we show that non-functional UCH-L1 drives podocyte injury through binding to and inhibiting proteasomal activity, which lastly results in severe proteotoxic stress and in the aggravation of MN.

## Methods

All uncropped and unprocessed scans of the most important blots are presented in the Source Data file or as a supplementary figure in the Supplementary Information. Patient samples were provided by the Hamburg GN Registry. Patients provided written consent for the use of samples for research. The study was approved by the registries board. Similar samples cannot be accessed by external users. The presented research complies with all relevant ethical regulations for the patient studies (Hamburg GN Registry board: Prof. Dr. Wiech, PD. Dr. Hoxha, Prof. Dr. T.B. Huber) and the animal studies (Behörde für Justiz und Verbraucherschutz, Amt für Verbraucherschutz, Lebensmittelsicherheit und Veterinärwesen, Germany).

### Antibodies

Primary antibodies used for the study were: rat anti-UCH-L1 (immunofluorescence microscopy mouse (IF) 1:50, immunoblot (WB) 1:250, Sosna et al.^39^); mouse anti-UCH-L1 (IF human kidney 1:50, TSA-amplification, clone 13C4, Abcam); rabbit anti-UCH-L1 (WB 1:250, ab27053, Abcam); rabbit anti-SOD2 (IF 1:200, Origene); mouse anti-Flag (WB 1:1000, clone M2, Sigma-Aldrich); rabbit anti-α2 (WB 1:500, Cell Signaling); rabbit anti-α4 (WB 1:1000, laboratory stock); mouse anti-α6 (WB 1:1000, clone MCP20, Enzo); rabbit anti-β1c (WB 1:1000, PA1-978, Invitrogen); rabbit anti-β2c (WB 1:1000, PA5-30988, Invitrogen); rabbit anti-β5c (WB 1:1000, PA1-977, Invitrogen); rabbit anti-β5c (IF 1:300, WB 1:5000, X. Wang, University of South Dakota, USA); rabbit anti-β5i (IF, 1:300, WB 1:5000, laboratory stock); rabbit anti-ubiquitin (IF 1:300, NB300-129, Novus); mouse anti-ubiquitin (WB 1:250, clone Ubi-1, Millipore); rabbit anti-K48-polyubiquitin (IF 1:300, ab140601, Abcam); rabbit anti-K48-polyubiquitin (WB 1:1000, clone Apu2, Millipore); guinea-pig anti-nephrin (IF 1:200, WB 1:2000, GP-N2, Progen); rabbit anti-α-actinin 4 (IF 1:200, clone IG-701, ImmunoGlobe); rabbit anti-p57 (IF 1:400, Santa Cruz); rhodamine-wheat germ agglutinin (IF 1:400, WGA, Vector); rat anti-HA (WB 1:1000, clone 3F10, Roche); rabbit pan-proteasomal antibody (IP 2 µg, laboratory stock); mouse anti-β-actin (WB 1:10000, clone AC-15, Sigma-Aldrich); for the detection of aggresomes PROTEOSTAT® Protein aggregation assay (ENZO, ENZ-51023-KP050). All secondary antibodies used were either biotinylated, HRP- or fluorescent dye-conjugated affinity purified donkey antibodies (Jackson ImmunoResearch).

### Generation of the murine WT and I93M-UCH-L1 full-length knock-in target vector

To generate the Rosa26 based knock-in, target vector construct of the complete mouse wildtype or I93M UCH-L1 protein, 1 µg of total RNA preparation was reverse transcribed using M-MLV-reverse transcriptase (200 U, 50 minutes at 37 °C, Thermo Fisher Scientific) and 100 ng Oligo dT-primer. For PCR, 2 µl cDNA was specifically amplified with 10 mM mUCH-L1-forward (Fw) 5’ *AA AAA A*GG CGC GCC GCG AAG ATG CAG CTG AAG CCG AT ’3 and -reverse (Rev) 5’ *AA AAA A*GG CGC GCC TTA AGC TGC TTT GCA GAG AGC CAC GG ’3 primers, respectively, including an AA overhang (cursive) and AscI sites (underlined) at their 5´ ends according to the mUCH-L1 sequence (NM_011670). PCR was performed for 35 cycles (denaturing: 15 sec at 98 °C, annealing: 15 sec at 66 °C, extension 1 min at 72 °C) using 5 U Phusion-DNA polymerase (Thermo Fisher Scientific) and subsequently cut for two hours at 37 °C with 10 U AscI (NEB, Ipswich, MA, USA). For cloning, 5 µg of the Rosa26/CAG/Stop/eGFP knock-in target vector (Addgene, #15192) was also digested over night at 37 °C with 25 U AscI (NEB)^50^. The DNA-fragment and the vector were purified by phenol/chloroform extraction and ethanol-precipitation for one hour at −80 °C. Ligation was performed with 100 ng vector-DNA and different molar ratios of the PCR-fragment (3:1, 1:1, 1:3) using 1 U T4-DNA ligase (NEB) at 12 °C overnight and subsequently transformed in competent XL10 *E. coli* (Agilent Technologies, Santa Clara, CA, USA) for 30 seconds at 42 °C. The cloning was verified by PCR screening and DNA-sequencing of the complete mUCH-L1 cDNA at Seqlab (Göttingen, Germany) according to their recommendations. Finally, 70 µg of one positive mUCH-L1 full-length clone was linearized in 300 µl 10 mM Tris/HCl pH 8.0 with 200 U AsiSI (NEB) over night at 37 °C, purified by sequential phenol and chloroform extractions and ethanol precipitated. The dried DNA-pellet was reconstituted in H_2_O and integrity was verified by agarose gel electrophoresis.

### Generation of transgenic UCH-L1 mouse models

*Uchl1*^fl/fl^ mice were generated by Genoway^17^ by insertion of lox*P* sites flanking the putative promoter region and exon 3 of the murine *Uchl1* gene. To obtain a podocyte-specific deletion of *Uchl1*, *Uchl1*^fl/fl^ mice were crossed to *Nphs2*-Cre mice^51^ and backcrossed to the C57BL/6 background. For experiments, *Uchl1*^*d/d;cre+*^ (hereafter called *Uchl1*^Δpod^)^17^ were used and compared to *Uchl1*^*+/+;cre+*^ or *Uchl1*^*d/d;cre-*^ littermates.

Podocyte-specific *Uchl1*^*WT*^ and *Uchl1*^*I93M*^ overexpressing mice were generated as follows. Briefly, R1-embryonic stem (ES)-cells (kind gift from Dr. Nagy, Samuel Lunenfeld Research Institute, Mount Sinai Hospital, Toronto, Canada^52^) were obtained at passage 11 and expanded to passage 14 and 15. R1-ES-cells were grown on gelatinated cell culture plates supplemented with a confluent layer of inactivated mouse embryo fibroblast (MEF) feeder cells. R1-ES-cells were passaged every second day. In order to obtain mice that could overexpress mouse WT or I93M-UCH-L1, ES-cells containing a Rosa26/CAG/Stop/mWT-UCH-L1 or mI93M-UCH-L1 knock-in target vector^50^ were generated as detailed above and 1 × 10^7^ R1-ES-cells were electroporated with 50 µg of the linearized target vector in a 0.4 cm gap-electroporation-cuvette (Life Technologies) and subsequently selected in a medium containing 200 µg geneticin (G418, Life Technologies) for one week. Correct targeting was verified by Southern blotting of EcoRI digested genomic DNA derived from isolated clones using a 5’ external ROSA probe. Positive R1-ES-cell clones were further expanded before injection into blastocysts. For blastocyst injection, 15 trypsinized R1-ES-cells with clearly visible, small, round nuclei without vacuoles were collected and injected into one blastocyst. Eight of these blastocysts were implanted into the uterus of a female mouse mated with vasectomized males at day 2.5 post coitum. The resulting chimeric males were mated with wild-type C57BL/6 females. Littermates of the F1 generation were genotyped by PCR to obtain offsprings that pass on the mWT or mI93M-UCH-L1 knock-in. These mice carried the transgenic information of mWT- or mI93M-UCH-L1 under the control of a floxed stop-cassette to prevent transactivation by the CAG-enhancer. These animals are referred to as mWT-or mI93M-UCH-L1-negative precursor mice and were further used for breeding with *Nphs2*-Cre mice^51^ to obtain a podocyte-specific mWT- or mI93M-UCH-L1 expression. Due to the podocyte-specific action of the *Nphs2* (podocin) promoter-triggered Cre-recombinase, the stop cassette was removed and mWT- or mI93M-UCH-L1 overexpression on glomerular podocytes was initiated. For experiments *Uchl1*^*WT+/fl*;cre+^ (hereafter called WT-UCH-L1) and *Uchl1*^*I93M+fl/;cre+*^(hereafter called I93M-UCH-L1) were backcrossed to the C75BL/6 background and compared to *Uchl1*^*WT+/fl*;cre-^ or *Uchl1*^*I93M+/fl*;cre-^ littermates, respectively. Figure 4a depicts a scheme of all the mouse lines used for the study.

The different genotypes were verified by PCR. Briefly, for PCR reaction, tail biopsies were lysed in DirectPCR (Tail) (Viagen 102-T) with Proteinase K (20 mg/ml, Sigma P6556-1G) overnight at 55 °C. Primers for the Cre-locus were: cre-Fw: 5’-GCA TAA CCA GTG AAA CAG CAT TGC TG-3’ and cre-Rev: 5’-GGA CAT GTT CAG GGA TCG CCA GGC G-3’; for the flox-locus of *Uchl1*^*fl/fl*^ mice flox-Fw: 5’-GCA CCA GTG ATT CAG CTT TCT AAA AGG AAC-3’ and flox Rev: 5’-TCT CAC CTG ACT GTA AAC TGA TGA GGG C-3’; for the R26-knockin locus Fw: 5’-GTT ACT ATG GGA ACA TAC GTC ATT-3’ and Uchl1 Rev: 5’-GTT GCA ATA CCT TTC TGG GAG TT-3’; and for Uchl1 wildtype Fw: 5’-CGA AAA TCT GTG GGA AGT CTT GT-3’ combined with Uchl1 Rev primer. Gel electrophoresis was performed with a 1,5% agarose gel.

### Animal experimentation

Mice were housed in a pathogen-free animal facility at the University Medical Center Hamburg-Eppendorf. Mice used in the study were older than 8 weeks of age and were predominantly analyzed at 10–38 weeks of age. Mice were kept at ambient temperature and humidity, had free access to water and standard animal chow (Altromin 1328 P) and were synchronized to a 12 h light/12 h dark cycle. All experimental procedures were performed according to the institutional guidelines. Anti-podocyte nephritis was induced as published^27^ by intravenous injection of 225 µl of sheep anti-podocyte antibodies or equal amounts of unspecific pre-immune sheep IgG. Urine was collected on day −1 prior to induction of experimental MN and on days 3, 6, 9 and 13 after induction of experimental MN. Kidneys were removed and serum collected on day 14 after disease induction. For induction of THSD7A-associated MN I93M-UCH-L1 mice were backcrossed for 3 generations with BALB/c to enhance susceptibility to the rabbit anti-THSD7A antibodies^30^. THSD7A-MN was induced by intravenous injection of purified anti-THSD7A rabbit IgG or unspecific control IgG as described^30^. Kidneys were removed and serum collected on day 12 after disease induction. Animal euthanasia was performed following buprenorphine (0,1 mg/kg KG s.c.) administration for analgesia 30 minutes prior to cervical neck dissection under 3.5% isoflurane inhalation narcosis.

### Glomerular isolation

To isolate glomeruli, mice were euthanized. Kidneys were dissected, perfused with HBSS containing magnetic beads (450 µm, Invitrogen and Spherotec)^53^ through the renal arteries. The kidney capsule was removed, the kidney shred into small pieces and digested with 1,2 mg/ml collagenase (Sigma), 100 U/ml DNase (Roche) in HBSS (Thermo Fisher Scientific) for 15 min at 37 °C under constant agitation at 1300 rpm. The suspension was homogenized through a 100 µm filter and pelleted in a magnetic field. Glomeruli were washed multiple times with HBSS supplemented with 0.05% BSA (Sigma) and preparation quality and glomerular count was assessed microscopically.

### Urine analyses

*Urinary albumin to creatinine ratio:* Mouse urine was collected in metabolic cages. Urine albumin content was quantified using a commercially available ELISA system (Bethyl) according to the manufacturer’s instructions, using an ELISA plate reader (BioTek), as described^37^. Urinary albumin values were standardized against urine creatinine values of the same individuals determined by Jaffe (Hengler analytic) and plotted.

*Stainfree SDS-PAGE:* Stainfree protein profile analyses of creatinine-adapted urines on day 14 after disease induction were performed by loading creatinine-adapted urines (UCH-L1^WT^ and Ctrl littermates: 0.69 µg creatinine; UCH-L1^I93M^ and Ctrl littermates: 0,55 µg creatinine; UCH-L1^Δpod^ and Ctrl littermates: 0.24 µg creatinine). Similarly to Coomassie or silver stain approaches, this method allows for the “unspecific” visualization of protein by utilizing a polyacrylamide containing trihalo compound (BioRad), which is covalently bound to tryptophan residues. Upon photoactivation with UV light, proteins fluoresce directly in-gel.

### Cell culture

Undifferentiated murine podocytes^54^ were cultured in RPMI supplied with 10% FCS, 1% penicillin/streptomycin, 15 mM HEPES, 1 mM sodium pyruvate and 10 U/ml Interferon γ at 32 °C and 5% CO_2_. For differentiation, interferon γ was removed from the medium and podocytes were cultured at 37 °C and 5% CO_2_ for at least 14 days. For inducible overexpression of UCH-L1, the Retro-X^TM^ Tet-On Advanced Inducible expression system (Clontech) was used as described^39^. To generate the WT-UCH-L1 and I93M-UCH-L1 tet-on cells, podocytes were transduced with the pRetroX-Tet-On Advanced vector and with the pRetroX-Tight-Pur-WT-UCH-L1 or pRetroX-Tight-Pur-I93M-UCH-L1 vector. For the generation of negative control cells (tet-, mock), podocytes were transduced with the pRetroX-Tet-On Advanced vector and the pRetroX-Tight-Pur empty vector. For induction of UCH-L1 overexpression, UCH-L1 tet-on or tet- podocytes were cultured with 5 ng/ml doxycycline^40^. For stable knockdown experiments, shRNA to murine UCH-L1 (shRNA627 and shRNA817) or scrambled shRNA for control was overexpressed in podocytes^38^. To analyze podocytes in the experimental MN cell culture model, podocytes were treated for 1, 2, 4 h with 1% sheep anti-podocyte antibody or pre-immune sheep IgG in standard medium and then prepared for further mRNA and protein analysis.

For the induction of oxidative stress, podocytes were exposed to 150 µM xanthine (X, Sigma) and 200 mU xanthine oxidase (XO, Sigma) in standard medium for 1, 2, 4, 6 h. Controls were treated instead with equal volumes of solvents sodium hydroxide (xanthine) or potassium phosphate buffer (xanthine oxidase). Harvested cells were processed for further mRNA and protein analysis. Reactive oxygen species were scavenged with 10 mM sodium pyruvate (Gibco), 5000 U/ml pancreatic catalase (Sigma), 100 mM mannitol (Sigma), 100 µM carboxy-PTIO (Sigma), beginning 30 min after initiation of X/XO exposure.

HEK293T cells (Sigma, # 12022001) were cultured in DMEM supplied with 10% FCS and 1% penicillin/streptomycin at 37 °C and 5% CO_2_. Transient transfection of HEK293T cells with hu-WT-UCH-L1, hu-I93M-UCH-L1 and hu-THSD7A constructs was performed by the calcium phosphate precipitation method. For that purpose, 10 µg of respective plasmid DNA was thoroughly mixed with 2 M CaCl_2_ and 2x HBS (280 mM NaCl, 10 mM KCl, 1,5 mM Na_2_HPO_4_ x 2 H_2_O, 12 mM glucose, 50 mM HEPES, pH 7.05) and after incubation for 30 min at room temperature added to <50% confluent HEK293T cells followed by overnight incubation at 37 °C and 5% CO_2_ and medium change on day 2. Harvested cells were prepared for subsequent proteasomal activity assays and screening of patient sera.

### Quantitative PCR

Total messenger RNA was extracted from murine podocytes using phenol/chloroform extraction. Cell pellets were lysed with TRIzol (Ambion) and incubated with 1/6 volume chloroform to separate RNA. For RNA purification isopropanol was added to the aqueous phase for 30 min at 4 °C. RNA pellet was washed twice with 80% ethanol and afterwards solved in purified H_2_O. 200 µg of extracted RNA were reverse transcribed with random hexamer primer (Invitrogen) and RevertAid reverse transcriptase (Thermo Scientific) in a thermocycler (Biometra) at 25 °C for 10 min, 42 °C for 1 h, 70 °C for 10 min, 4 °C forever. mRNA expression was quantified with the QuantStudio 3 (Applied Biosystems) qPCR cycler using SYBR green. Exon spanning primer pairs to murine cDNA were used: *Uchl1 (fw*: 5´-AGC TGG AAT TTG AGG ATG GA-3´; *rev*: 5´-GGC CTC GTT CTT CTC GAA A-3´), *Sod2* (*fw:* 5´-ACA ACT CAG GTC GCT CTT CAG-3´; *rev:* 5´-TCC AGC AAC TCT CCT TTG GG-3´), *Gpx4* (*fw*: 5´-CAT TCC TGA ACC TTT CAA CCC G-3´; *rev*: 5´-ATG CAC ACA AGC CCA GGA ACT-3´), *Trx* (*fw*: 5´-AAA GGG TCA AAA GGT GGG GG-3´; *rev*: 5´-ACA GCT GGT AGC TGG TTA CAC-3´). *18* *S* (*fw*: 5´-CAC GGC CGG TAC AGT GAA AC-3´; *rev*: AGA GGA GCG AGC GAC CAA A-3´) was used as housekeeping control to normalize mRNA levels. Relative gene expression was calculated appropriating the ΔΔCT method.

### Immunoblot analysis

All immunoblots were performed with isolated glomeruli or cultured podocytes. Samples were lysed in T-Per (Thermo Fisher Scientific) containing complete EDTA-free (Roche) and denatured for 10 min at 95 °C in SDS solubilization buffer. Samples were separated on a 4-15% MiniProtean TGX gel (BioRad) in a Tris-glycine migration buffer (0.25 M Tris base, 1.92 M glycine, 1% SDS, pH 8.3). Protein transfer was performed in transfer buffer (0.25 M Tris base, 1.92 M glycine, 20% MeOH in H_2_O) in a TransBlot Turbo System (BioRad). After the transfer, all proteins were visualized by ponceau staining. PVDF membranes (Millipore) were blocked (3% non-fat milk) prior to incubation with primary antibodies diluted in Superblock reagent (Thermo Fisher Scientific) or non-fat milk. Binding was detected by incubation with HRP-coupled secondary antibodies (1:10000 − 1:20000, 3% non-fat milk). Protein expression was visualized with ECL SuperSignal (Thermo Fisher Scientific) according to manufacturer’s instructions on an Amersham Imager 600 (GE Healthcare). Immunoblots were analyzed using software from ImageJ^55^. β-actin stainings of the same membrane are shown and were used as loading control and for densitometric normalization. Bands of the same membrane are shown, fine dashed black lines indicate, where bands were not adjacent to another on the membrane.

### Ubiquitin derived activity-based DUB activity assay

Levels of enzymatic-active UCH-L1 or DUBs in glomerular lysates were assessed, using the ubiquitin derived activity-based ubiquitin-vinylmethylester probe (Ub-VME), which is an active-site directed probe with broad reactivity towards DUBs with the exception of the few metalloproteases^56^. This probe binds covalently to the active-site (Cys) of most DUBs, including UCH-L1, which is only accessible in active enzymes. The N-terminal epitope Cy5-tag allows detecting the modified DUBs after the separation of a protein mixture by SDS-PAGE, followed by fluorescent detection. Briefly, glomeruli-adapted lysates (TPer lysis buffer (Pierce) with 250 U/ml benzonase) in an end volume of 20 µl labeling buffer (50 mM Tris, pH 7.4, 5 mM MgCl_2_, 250 mM sucrose, 1 mM DTT, and 2 mM ATP) were labeled with 2.24 µM ubiquitin derived activity-based ubiquitin-vinylmethylester probe (Cy5-Ub-VME) and incubated for 1 h at 37 °C. The reaction was stopped while adding DTT containing solubilizer and heating at 70 °C for 10 min. Proteins were resolved by SDS-PAGE on 12.5% tris-glycine gels, fluorescent detection was performed applying a Capsule 640 nm and F-710 interference filter and using a FUSION FX07 (Vilber).

### Purification of UCH-L1 protein

Overexpressed WT- or I93M-UCH-L1-flag constructs from HEK293T cells were purified using anti-Flag M2 Affinity Gel (Sigma) according to the manufacturer’s instructions. Therefore approximately 10 µg total HEK293T lysates (TPer lysis buffer supplemented with complete EDTA-free) were used and flag protein could be eluted by IgG elution buffer pH 2.8 (ThermoScientific) with subsequent rebuffering into PBS using Zebra Spin Desalting Columns, 7 K MWCO (ThermoScientific). The outcome and purity was examined by SDS-PAGE on 4-15% MiniProtean TGX gels (BioRad).

### Proteasomal activity assays

In vitro chymotrypsin-like activity assay was performed using 10 µg purified WT- or I93M-UCH-L1-flag constructs incubated with 1 nM purified human (hu) 20S and 15 nM purified hu26S proteasome (BostonBiochem), respectively as well as hu20S activator (0.035%) SDS in incubation buffer (20 mM HEPES, 0.5 mM EDTA, 5 mM DTT, 1 mg ovalbumin, 60 mM ATP, pH 7.8) to a final volume of 50 µl. After preincubation for 2 hours at 4 °C, 60 µM Suc-LLVY-AMC substrate (Calbiochem, EMD Chemicals, Inc, division of Merck KGaA, Darmstadt, Germany) in incubation buffer was mixed with the samples. Proteasomal activity was measured at 355 nm and 460 nm using a fluorescent spectrophotometer (Mithras LB 940; Berthold Technologies GmbH & Co., Wildbad, Germany) after incubation at 37 °C for 30, 60, 90, 120 and 180 min in the dark.

For further evaluation of chymotrypsin-like activity 20 µg HEK293T cells overexpressing WT- or I93M-UCH-L1 mixed with 5x clear native loading buffer (250 mM Bis-Tris, pH 6.5, 250 mM NaCl, 50% glycerol, 0.25% bromphenol blue) excluding heating were resolved using a native PAGE. After electrophoresis gels were incubated with 100 µM Suc-LLVY-AMC substrate (see above) in activity buffer (20 mM Tris base, pH 7.5, 5 mM MgCl_2_, 2 mM ATP). The chymotrypsin-like activity of proteasomal complexes could be visualized by measuring fluorescence applying a Capsule 365 nm and F-450 interference filter using a FUSION FX07 (Vilber).

Proteasomal subunit activities were investigated using a pan-reactive β subunits activity-based probe MVB003. To this end HEK293T cells were freeze-thawed (7 cycles) in TSDG buffer (10 mM Tris HCl, pH 7.5, 10 mM NaCl, 25 mM KCl, 1 mM MgCl_2_, 0.1 mM EDTA, 10% glycerol, 1 mM DTT, 2 mM ATP) using an ethanol/dry ice mixture following centrifugation for 10 min with 16000 *g* at 4 °C. Protein concentration of the supernatant was determined by BCA assay. 20 µg total protein was mixed with 0.5 µM MVB003 and filled up to a total volume of 20 µl with TSDG buffer and incubated for 1 h at 37 °C. As a control, 20 µg of total protein from kidney tissue of BALB/c mice were incubated with either 2 µM epoxomicin (negative control) or equal volumes DMSO (positive control) for 1 h at 37 °C, followed by an incubation with 0.5 µM MVB003. After solubilization with DTT containing loading buffer for 10 min at 70 °C samples were resolved by SDS-PAGE on 12.5% tris-glycine gels with 3.3% crosslinker (acrylamide:bis 29:1). Fluorescent detection was performed applying a Capsule 530 nm and F-595 interference filter using a FUSION FX07 (Vilber) followed by subsequent blotting of the gel to determine total expression of β subunits for activity normalization.

### Immunoprecipitation

Proteins or protein complexes were precipitated from 150 µg total cell lysate in 1 ml TSDG buffer (20 mM Tris HCl, pH 7, 10 mM NaCl, 25 mM KCl, 1.1 mM MgCl_2_, 0.1 mM EDTA, 10% glycerol, 2 mM ATP, 0.5 mM DTT, complete w/o EDTA, 1 mg/ml bovine serum albumin, 0.1% NP40) over night at 4 °C under gentle rotation with 2 µg antibody as indicated. The antibody protein complex was incubated for 2 hours with protein G sepharose (Roche) and pulled-down by centrifugation. After 2 washing steps with PBS the proteins were eluted by SDS-PAGE sample buffer and employed for immunoblotting.

### Glycerol gradient fractionation

Protein complexes of 3 mg total protein in TSDG buffer were separated by glycerol gradient ultracentrifugation using a gradient from 10–40% glycerol and ultracentrifugation at 283.000 *g* for 16 hours. The gradients were fractionated into 1 ml fractions, 50 µl of the fractions were assayed for chymotrypsin-like proteasome activity using Suc-LLVY-AMC (see above) and 20 µl were analyzed by immunoblotting.

### Immunofluorescence

Kidney cortex was embedded in paraffin for light or high-resolution confocal microscopy. For light microscopic evaluation, 1.5 µm thick sections were cut on a rotation microtome and stained with periodic-acid-Schiff reagent (Merck) according to the manufacturer’s instructions. For immunofluorescent stainings, 3 µm paraffin sections were deparaffinized and antigen retrieval was performed by steamer boiling (10 mM citrate buffer, pH 6.1) or by protease XXIV (Sigma-Aldrich, 5 µg/ml) digestion. Unspecific binding was blocked in 5% horse serum and 0.05% TritonX-100 for 30 min. Primary antibody incubations (in blocking buffer, o/n, 4 °C) were followed by incubation with biotinylated or AF488-, AF647-, AF586-, Cy3-, or Cy5-coupled secondary antibodies (1:400, 30 min RT). Nuclei were visualized using Hoechst (1:1000, Molecular Probes). Stainings were evaluated with an LSM510 meta microscope for conventional microscopy or with an LSM800 with airyscan for high-resolution confocal microscopy using LSM or ZENblue software (all Zeiss).

### Quantification of podocyte loss

For quantification of podocyte loss, 3 µM paraffin sections were stained for the nuclear marker p57 using the ZytoChem-Plus AP Polymer Kit (Zytomed Systems) and Neufuchsin (Merck) according to the manufacturer’s instructions for color development. 50 glomeruli per mouse per condition were analyzed for glomerular tuft area and respective number of p57-positive podocytes in a double blinded fashion at a 400fold magnification.

### Podocyte Exact Morphology Measurement Procedure (PEMP)

Visualization of foot process morphology, and analysis of filtration slit density was performed^57,58^. In brief, 3 μm paraffin sections mounted on high-precision coverslips coated with Poly-L-lysine (Sigma) were stained for nephrin. Stained sections were mounted with ProLong Gold (Thermo Fisher) for imaging. For visualization super-resolution STED microscopy was performed using a 4 channel STED microscope (Abberior). For quantification, 3D-SIM z-stacks of nephrin-stained kidney sections were acquired with a Zeiss Elyra PS.1 SIM microscope using the ZEN software (all Zeiss, Jena, Germany) with five horizontal shifts and five rotations of the illumination pattern with a slice-to-slice distance of 0.3 µm. Reconstruction of 3D-SIM images was performed using the Zen Black software. PEMP analysis was performed using the PEMP macro for FIJI^58^. For this, the capillary area was encircled, and the slit diaphragm length was determined. Filtration slit density (FSD) values were calculated from the ratio of slit diaphragm length and capillary area. Per animal, 12 glomeruli with 31-39 ROIs were analyzed by PEMP.

### Electron microscopy

For electron-microscopical analyses, small cortical samples fixed in 4% buffered paraformaldehyde were used. Tissue was post-fixed with 1% osmium in 0.1 M phosphate buffer (1 h at RT), stained with 1% uranylacetate (1 h at RT in 70% ethanol), dehydrated and embedded in epoxy-resin (Durcupan, Sigma-Aldrich). Ultrathin sections were cut (Ultramicrotome UC6, Leica) and contrasted with lead citrate. Micrographs were generated with a transmission-electron microscope (TEM 910, Zeiss, Oberkochen, Germany).

### Patient analysis for UCH-L1 expression and autoantibody formation

For the correlative analysis of clinical outcome to podocyte UCH-L1 expression in membranous nephropathy, 28 patient biopsies from the Hamburger GN register were stained for UCH-L1 using the mouse mAB anti-UCH-L1 antibody (13C4, 1:50, Abcam, using the TSA amplification kit, New England Biolabs), nuclei were counterstained using hematoxylin. Biopsies with no glomeruli or with an absent UCH-L1 signal in distal tubuli were excluded from the analyses, due to technical issues in antigen-preservation and detectability. UCH-L1 immunosignal was classified into “low-medium-high” in relation to the distal tubular staining intensity within the 27  included biopsies in a double-blinded manner. Correlation analyses to clinical outcome were performed using Student *t*-test and Fisher’s exact test.

The determination of sero-positivity for anti-UCH-L1 autoantibodies was performed in a double-blinded manner. Briefly, for the detection of anti-UCH-L1 autoantibodies in 78 MN patient sera and 9 healthy control sera, human WT or mutant I93M UCH-L1 protein fused to both an N-terminal flag and a myc tag were expressed in Freestyle293-F cells (Thermo Fisher, #100044202) and purified by affinity chromatography using anti-Flag M2 Affinity Gel (Sigma Aldrich). UCH-L1 protein was eluted by pH shift. The purity of the elution fraction and protein concentration was checked by SDS PAGE and the stainfree technology (Biorad). Additionally, protein concentration was confirmed by photometrics, and proteins were separated by SDS PAGE (4–15% gradient gel, Biorad) and blotted onto PVDF membrane (Merck Millipore) using a Transblot Turbo device (Biorad). After blotting, the membrane was blocked in 3% non-fat milk in TBST (10 mM Tris-HCl pH 7.4, 100 mM NaCl, 0,05% Tween-20) for 1 h at RT. Membrane snippets were cut in a way that each snippet contained one lane molecular weight marker (HighQu), one lane WT protein and one lane mutant protein. Each snippet was incubated in a 2 mL tube with 20 µL of patient serum in 1 mL of 3% non-fat milk in TBST on a roller shaker (Cole-Parmer) over night at 4 °C. Snippets were washed 3 times for 10 minutes with TBST followed by an incubation with affinity purified HRP-coupled human anti-IgG+M (Jackson measurement (Nanophotometer, Implen). For sera analyses, 700 ng of each WT and I93M protein were mixed with reducing sample buffer (50 mM Tris-HCl pH 6.8, 2% SDS, 100 mM DTT, 10% glycerol, 0.05% bromphenol blue; final concentrations) and boiled at 95 °C for 5 min. Samples Immunoresearch Laboratories) for 1 h at RT. After another 3 washes with TBST and an incubation of 5 min in SuperSignal West Pico Plus Chemiluminescent Substrate (Thermo Fisher) the snippets were imaged using a Vilber Fusion FX07 system. Maximum detection time was set at 3 min. To confirm that protein was loaded on each snippet, the snippets were stripped for 15 minutes in 200 mM Glycin, 0.1% SDS, 1% Tween 20 pH 2.2 followed by a reprobe with an anti-myc antibody (Santa Cruz sc-40, 1:400 in 3% non-fat milk in TBST).

To quantify the intensity of the lanes, densitometry was done by using the rolling ball method for background substraction with the EvolutionCapt Software (Vilber). The Software was allowed to determine the optimal rolling ball size automatically. Also, the UCH-L1 signals that were detected by using the patient sera as primary antibodies were normalized to the intensity of the signals detected with the myc antibody.

### Structural analysis and docking

For the calculation of the electrostatic surface potential the published crystal structures of Ubiquitin carboxy-terminal hydrolase L1 (UCH-L1^WT^, PDB entry 2ETL, and its I93M variant UCH-L1^I93M^, PDB entry 3IRT) were used. The respective wildtype structure was used to calculate the electrostatic surface potential of an oxidative-modified variant (UCH-L1^WT_ox_mod^). The oxidative modifications were defined as determined by Choi et al^.24^. Using the PyMOL plugin PyTMs (https://github.com/Pymol-Scripts/Pymol-script-repo/blob/master/plugins/pytms.py) the wildtype residues Met1, Met6, Met12, Met124 and Met179 were in silico mutated to their oxidated counterparts methionine sulfoxide. Additionally, the wildtype residue Cys220 was mutated in silico to its corresponding cysteine sulfonic acid. The published PDB files 2ETL and 3IRT as well as the calculated PDB file for the oxidative-modified variant were used to calculate the electrostatic surface potential applying the APBS-PDB2PQR software suite using the corresponding server (https://server.poissonboltzmann.org/). The software package PyMOL (https://pymol.org/2/) was used for visualization.

For the prediction of putative interactions between the human 20S proteasome (PDB entry 5LE5) and UCH-L1^WT^ (PDB entry 2ETL) or its I93M variant UCH-L1^I93M^ (PDB entry 3IRT) the respective published crystal structures were applied to Cluspro (https://cluspro.bu.edu) to calculate representative sets of lowest energy configurations for the docking of UCH-L1 variants to the human 20S proteasome. The software package PyMOL (https://pymol.org/2/) was used for visualization.

### Statistical analysis

All values are expressed as mean ± SEM and analyzed using Prism 8.2.1 for Mac OS X, GraphPad Software, San Diego, California USA, www.graphpad.com. Effects of genotype were analyzed with *t*-test (for two genotypes), or One-way ANOVA (for three genotypes) followed by Bonferroni post-hoc tests when appropriate. If data did not reach the criteria for parametric statistics, non-parametric statistics using Mann-Whitney *U* test or Kruskal-Wallis test followed by Dunn’s multiple comparisons if two or three genotypes, respectively, were tested. In time course analyses a mixed two-way ANOVA with Bonferroni post-hoc test was used. Non-parametric statistic was selected to enable robust conclusions on effects significance in case of departures from normality associated with small sample sizes. Replicates used were biological replicates, which were measured using samples derived from distinct mice. All animals were littermates and were blindly assigned to the experimental groups. Patient data were analyzed using ordinary One-way ANOVA with Tukey’s multiple comparisons test, Wilcoxon test, and Spearman correlation. Unless reported otherwise, all tests were two-tailed and significance was set at *p* ≤ 0.05.

### Reporting summary

Further information on research design is available in the Nature Portfolio Reporting Summary linked to this article.

## Supplementary information

Supplementary Information

Reporting Summary

## Data Availability

All original data generated in this study are accessible via the Supplementary Appendix. Source data are provided with this paper.

## Source data

Source Data
