# Publisher Correction: Non-functional ubiquitin C-terminal hydrolase L1 drives podocyte injury through impairing proteasomes in autoimmune glomerulonephritis

**DOI:** 10.1038/s41467-023-38206-0

**Published:** 2023-04-28

**Authors:** Julia Reichelt, Wiebke Sachs, Sarah Frömbling, Julia Fehlert, Maja Studencka-Turski, Anna Betz, Desiree Loreth, Lukas Blume, Susanne Witt, Sandra Pohl, Johannes Brand, Maire Czesla, Jan Knop, Bogdan I. Florea, Stephanie Zielinski, Marlies Sachs, Elion Hoxha, Irm Hermans-Borgmeyer, Gunther Zahner, Thorsten Wiech, Elke Krüger, Catherine Meyer-Schwesinger

**Affiliations:** 1 Institute of Cellular and Integrative Physiology, Center for Experimental Medicine, University Medical Center Hamburg-Eppendorf, Hamburg, Germany; 2 Institute of Medical Biochemistry and Molecular Biology, University Medicine Greifswald, Hamburg, Germany; 3 Protein production Core Facility, Centre for Structural Systems Biology, Deutsches Elektronen-Synchrotron DESY, Hamburg, Germany; 4 Skeletal Pathobiochemistry, Department of Osteology and Biomechanics, Center for Experimental Medicine, University Medical Center Hamburg-Eppendorf, Hamburg, Germany; 5 Bio-organic synthesis group, Leiden University, Leiden, The Netherlands; 6 III Medical Clinic and Polyclinic, Nephrology, University Medical Center Hamburg-Eppendorf, Hamburg, Germany; 7 Transgenic Animal Service Group, Center for Molecular Neurobiology Hamburg, University Medical Center Hamburg-Eppendorf, Hamburg, Germany; 8 Institute of Pathology, Nephropathology Section, University Medical Center Hamburg-Eppendorf, Hamburg, Germany

**Keywords:** Membranous nephropathy, Mechanisms of disease, Deubiquitylating enzymes

**Correction to**: *Nature Communications* 10.1038/s41467-023-37836-8, published online 13 April 2023

The original version of this Article contained errors in Figures 3b, 7d, 7e and 8b, where boxes were used instead of arrows to indicate the specific signals for the blots, and in Figure 8c, the format of the “betas“ was incorrect. This has been corrected in both the PDF and HTML versions of the Article.

