# Supplementary material for: Non-functional ubiquitin C-terminal hydrolase L1 drives podocyte injury through impairing proteasomes in autoimmune glomerulonephritis
Source: Nat Commun. 2023 Apr 13;14:2114. doi: 10.1038/s41467-023-37836-8 (PMC10102022; doi:10.1038/s41467-023-37836-8)
Supplement: Supplementary file 1 — Supplementary Information [file 41467_2023_37836_MOESM1_ESM.pdf]

Supplementary Material

Title *Non-functional Ubiquitin C-Terminal Hydrolase L1 Drives Podocyte Injury Through Impairing Proteasomes in Autoimmune Glomerulonephritis*

Authors Julia Reichelt, Wiebke Sachs, Sarah Frömbling, Julia Fehlert, Maja Studencka-Turski, Anna Betz, Desiree Loreth, Lukas Blume, Susanne Witt, Sandra Pohl, Johannes Brand, Maire Czesla, Jan Knop, Bogdan I. Florea, Stephanie Zielinski, Marlies Sachs, Elion Hoxha, Irm Hermans-Borgmeyer, Gunther Zahner, Thorsten Wiech, Elke Krüger, Catherine Meyer-Schwesinger

==

Supplementary Figures

Supplementary Figure 01. UCH-L1 protein is stabilized by proteasomal inhibition in podocytes.

Supplementary Figure 02. Upregulation of reactive oxygen species detoxifying enzymes in cultured murine podocytes.

Supplementary Figure 03. Polyclonal sheep anti-podocyte nephritis (APN) antibodies contain antibodies with reactivity to UCH-L1.

Supplementary Figure 04. UCH-L1 is *de novo* expressed in podocytes in human und murine membranous nephropathy.

Supplementary Figure 05. Expression of UCH-L1 protein in podocytes does not correlate with clinical disease outcome in patients with membranous nephropathy.

Supplementary Figure 06. I93M-UCH-L1 but not WT-UCH-L1 exhibits reduced hydrolytic activity.

Supplementary Figure 07. Mouse models of genetic modulation of UCH-L1 expression in podocytes.

Supplementary Figure 08. I93M-UCH-L1 but not WT-UCH-L1 exhibit ubiquitin accumulations in podocytes.

Supplementary Figure 09. Podocytes of UCH-L1 overexpressing but not UCH-L1 knockdown mice exhibit accumulations of slit diaphragm protein nephrin.

Supplementary Figure 10. Glomerular filtration barrier alterations are attenuated in UCH-L1Δpod mice in experimental membranous nephropathy.

Supplementary Figure 11. I93M-UCH-L1 but not WT-UCH-L1 mice exhibit severe foot process effacement in experimental membranous nephropathy.

Supplementary Figure 12. Overexpression of I93M-UCH-L1 aggravates podocyte injury in the THSD7A-associated model of membranous nephropathy (MN).

Supplementary Figure 13. The proteolytic β5c and β5i subunits of the proteasome co-localize with UCH-L1 in podocytes in experimental MN.

Supplementary Figure 14. Representative immunoblots depicting transfection efficiency of transiently transfected HEK293T cells used for proteasomal interaction studies in Fig. 6d.

Supplementary Figure 15. I93M-UCH-L1 and WT-UCH-L1 interact with the proteasome.

Supplementary Figure 16. Protein abundance of β1c, β2c and β5c in HEK293T cells transiently transfected with wildtype or I93M-UCH-L1.

Supplementary Figure 17. In silico docking predictions based on electrostatic potential of UCH-L1.

Uncropped gels and blots of the supplemental appendix.

Author/Institute Information

Institute of Cellular and Integrative Physiology, Center for Experimental Medicine, University Medical Center Hamburg-Eppendorf, Martinistraße 52, 20246 Hamburg, Germany.
Julia Reichelt, j.reichelt@uke.de
Wiebke Sachs, w.sachs@uke.de
Sarah Frömbling, s.froembling@uke.de
Julia Fehlert, julia.fehlert@gmail.com
Anna Betz, anna.betz@googlemail.com
Desiree Loreth, d.loreth@uke.de
Lukas Blume, lu.blume@uke.de
Johannes Brand, j.brand@uke.de
Maire Czesla, maire.czesla@gmail.com
Jan Knop, Jan.Knop@Hamburg.de
Stephanie Zielinski, s.zielinski@uke.de
Marlies Sachs, msachs@uke.uni-hamburg.de
Catherine Meyer-Schwesinger, c.meyer-schwesinger@uke.de

Institute of Medical Biochemistry and Molecular Biology, University Medicine Greifswald, Ferdinand-Sauerbruch-Straße / Klinikum DZ 7, 17475 Greifswald, Germany.
Maja Studencka-Turski, majastudencka.ms@gmail.com
Elke Krüger, elke.krueger@uni-greifswald.de

Protein production Core Facility, Centre for Structural Systems Biology, Deutsches Elektronen-Synchrotron DESY, Notkestraße 85, Building 15, 22607 Hamburg, Germany.
Susanne Witt, susanne.witt@cssb-hamburg.de

Skeletal Pathobiochemistry, Department of Osteology and Biomechanics, Center for Experimental Medicine, University Medical Center Hamburg-Eppendorf, Martinistraße 52, 20246 Hamburg, Germany.
Sandra Pohl, s.pohl@uke.de

Bio-organic synthesis group, Leiden University, Einsteinweg 55, 2333CC Leiden, the Netherlands.
Bogdan I. Florea, b.florea@chem.leidenuniv.nl

III. Medical Clinic and Polyclinic, Nephrology, University Medical Center Hamburg-Eppendorf, Martinistraße 52, 20246 Hamburg, Germany.
Elion Hoxha, e.hoxha@uke.de
Gunther Zahner, zahner@uke.de

Transgenic Animal Service Group, Center for Molecular Neurobiology Hamburg, University Medical Center Hamburg-Eppendorf, Falkenried 94, 20251 Hamburg, Germany.
Irm Hermans-Borgmeyer, hermans@zmnh.uni-hamburg.de

Institute of Pathology, Nephropathology Section, University Medical Center Hamburg-Eppendorf, Martinistraße 52, 20246 Hamburg, Germany.
Thorsten Wiech, t.wiech@uke.de

Supplementary Fig. 1

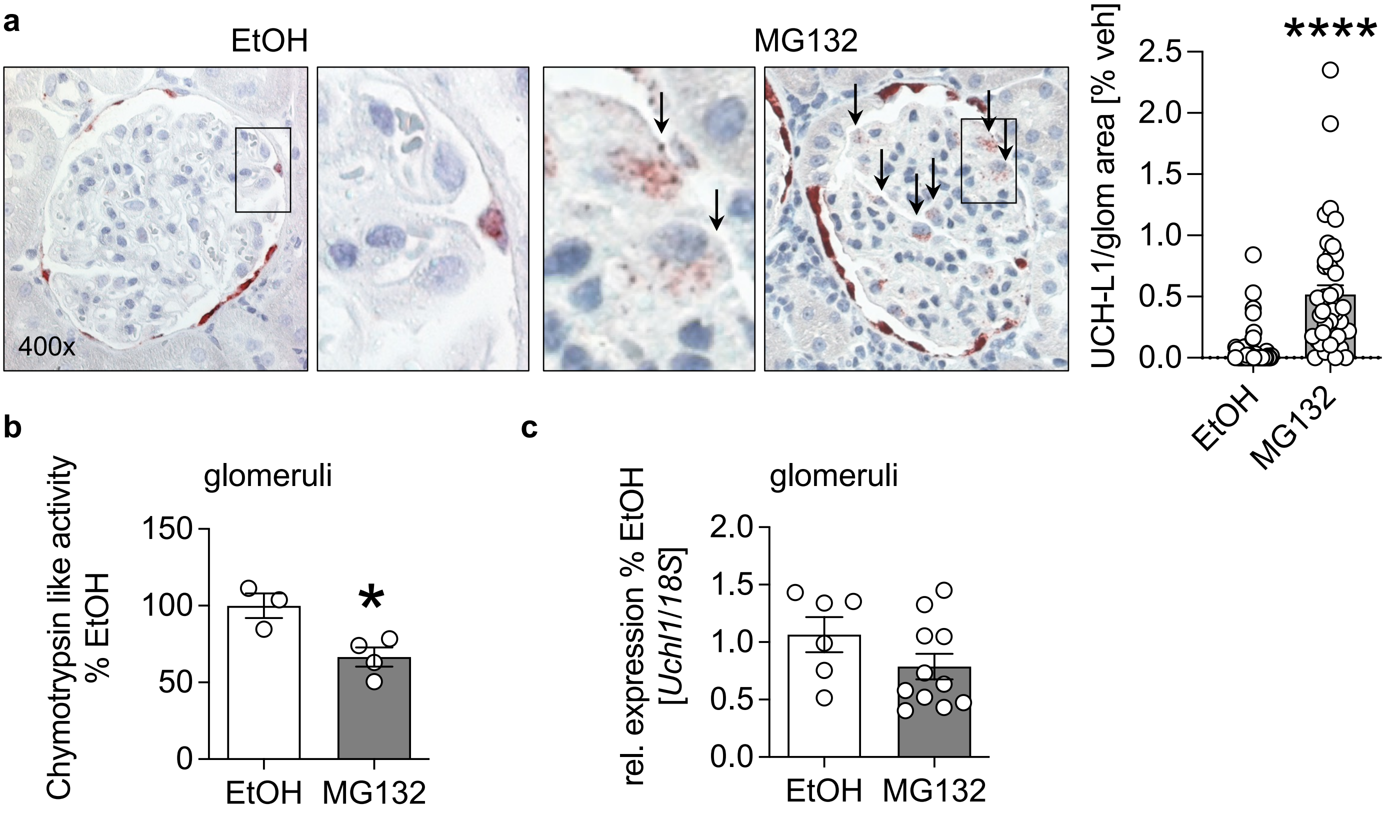

Suppl. Fig. 1: UCH-L1 protein is stabilized by proteasomal inhibition in podocytes. For proteasomal inhibition MG132 (60 mg/kg body-weight in 15% ethanol (EtOH)) or ethanol alone was introduced in 2ML Alzet osmotic minipumps, which were implanted subcutaneously for 14 days in Wistar rats. A kidney piece was processed for immunofluorescence, glomeruli were isolated by sieve technique. (**a**) Representative micrographs depicting UCH-L1 expression in podocytes (arrows) following proteasomal inhibition. Graph exhibits quantification of UCH-L1 immunosignal in n=2 rats per group, pooled data from 2 independent experiments, mean +/-SEM, ****p < 0.0001, two-tailed Mann Whitney U test. (**b**) Proteasomal chymotrypsin-like activity in isolated glomeruli showing successful proteasomal inhibition with MG132, which also exerts inhibitory effects on other proteases besides the proteasome, n=3 (EtOH) or n=4 (MG132), 1 representative experiment, mean +/-SEM, *p = 0.02, two-tailed Unpaired t-test. (**c**) Quantitative PCR of relative *Uchl1* expression normalized to *18S* in isolated glomeruli, pooled values of 2 independent experiments, n=6 (EtOH) or n=11 (MG132), mean +/-SEM, two-tailed Unpaired t-test.

Supplementary Fig. 2

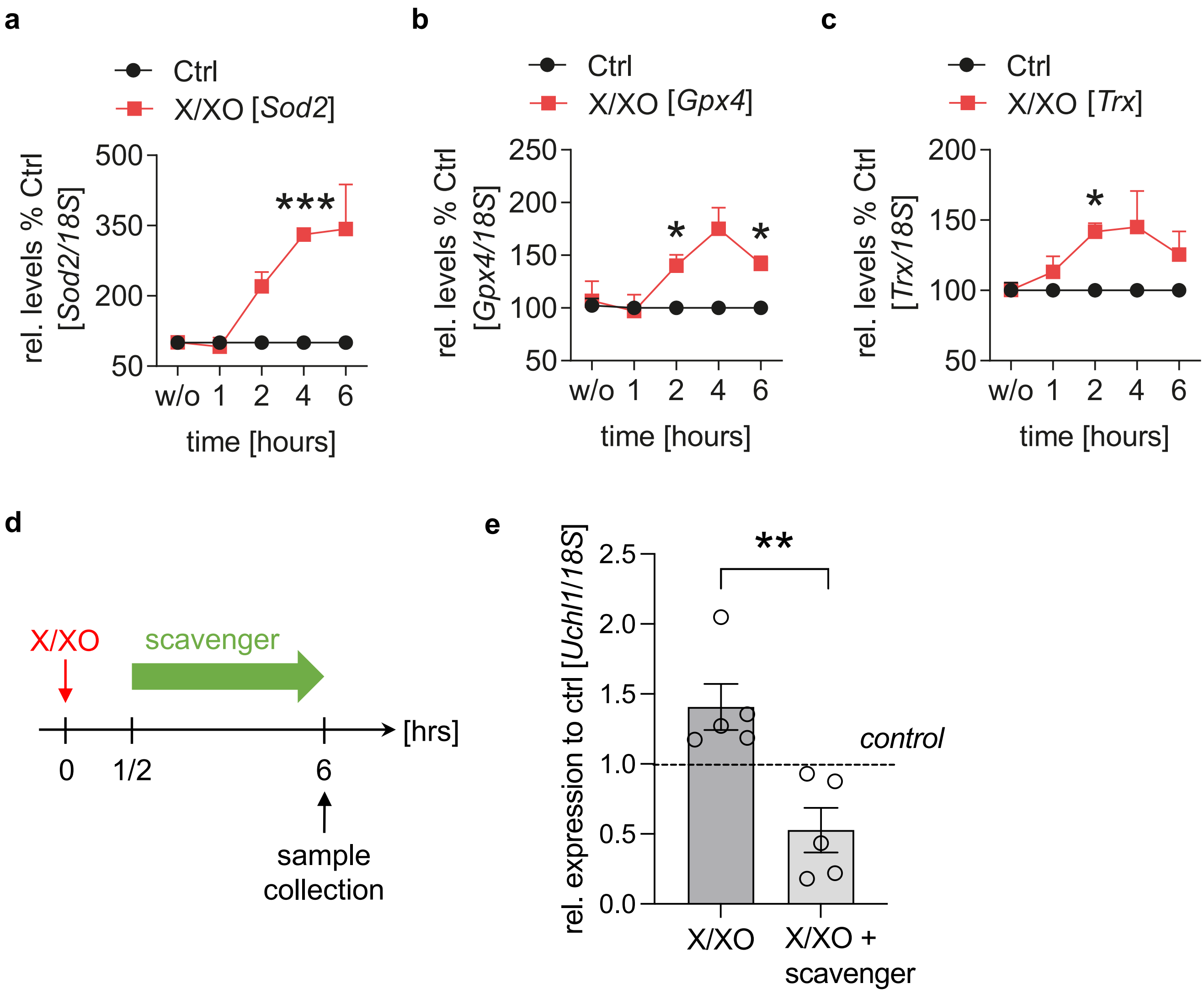

Suppl. Fig. 2: Upregulation of reactive oxygen species detoxifying enzymes in cultured murine podocytes. Immortalized murine podocytes were incubated for 0 (w/o), 1, 2, 4, and 6 hours with 200 mU xanthine oxidase and its substrate xanthine (150 µM) to induce the production of reactive oxygen species (ROS) or equal volume of NaOH and K_2HPO_4 (Ctrl). qPCR analyses for ROS-detoxifying transcripts (**a**) super oxide dismutase 2 (*Sod2*), (**b**) Phospholipid-Hydroxyperoxid-Glutathion-Peroxidase (*Gpx4)*, and (**c**) thioredoxin (*Trx)*. Relative transcript changes in X/XO (red line) in comparison to respective controls (Ctrl, black line) was assessed by normalization to *18S* as housekeeper. Pooled values of 4 independent experiments with n=1, mean +/-SEM. *p* from left to right, 0.0007, 0.0476, 0.0179, 0.0315; Two-Way ANOVA with Geisser-Greenhouse correction. (**d**) Experimental setup. To induce oxidative stress cultured murine podocytes were treated 6 hours with 150 µM xanthine (X) and 200 mU xanthine oxidase (XO) or equal volume of NaOH and K_2HPO_4 (Ctrl). Supplementation with a mixture of radical scavengers (10 mM sodium pyruvate, 5000 U/ml pancreatic catalase, 100 mM mannitol, 100 µM carboxy-PTIO) after 30 min of X/XO treatment provoke inhibition of generated reactive oxygen species. (**e**) Relative *Uchl1* transcript abundance of murine podocytes treated with X/XO and further those complemented with scavengers was assessed in comparison to control (dotted line) by qPCR after normalization to *18S* as housekeeper. Values of 1 independent experiment are presented as mean +/-SEM, n=5, ***p* = 0.0079, two-tailed Mann Whitney U test.

Supplementary Fig. 3

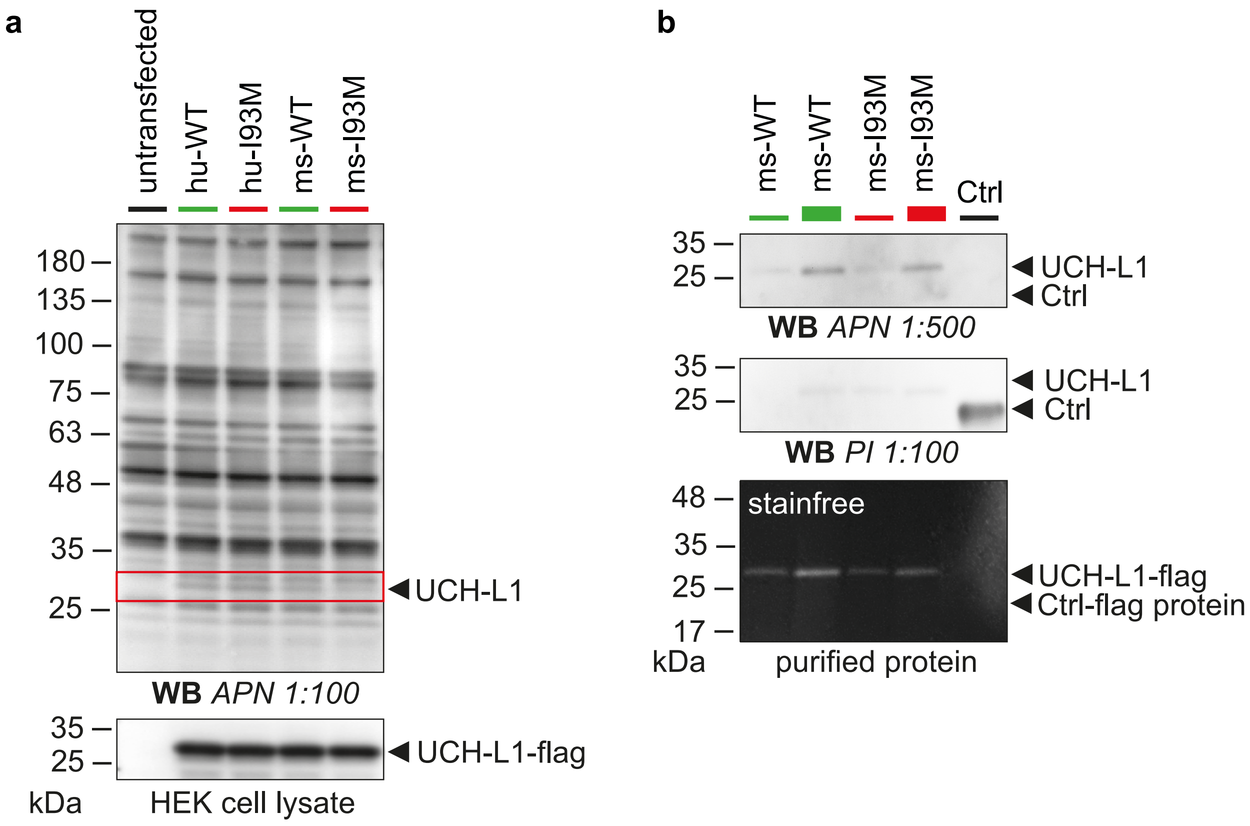

Suppl. Fig. 3: Polyclonal sheep anti-podocyte nephritis (APN) antibodies contain antibodies with reactivity to UCH-L1. (**a**) HEK293T cells were transfected with murine (ms) or human (hu) WT-UCH-L1-flag or I93M-UCH-L1-flag constructs, untransfected HEK293T cells were used as negative control. Total lysates were blotted and analyzed for a binding of sheep APN antibodies to the UCH-L1-flag constructs (red box). Immunoblot of 1 independent experiment to UCH-L1 depicts successful and equal expression of constructs in transfected cells. (**b**) Murine (ms) UCH-L1 constructs were flag-purified and 600 ng or 1.1 µg of purified protein was loaded, as well as a flag-tagged control protein (Ctrl). Immunoblot detection was performed with APN antibodies (1:500) or pre-immune (PI) antibodies (1:100). Stainfree shows purity and loading amount of purified proteins. Analyses of 1 independent experiment.

Supplementary Fig. 4

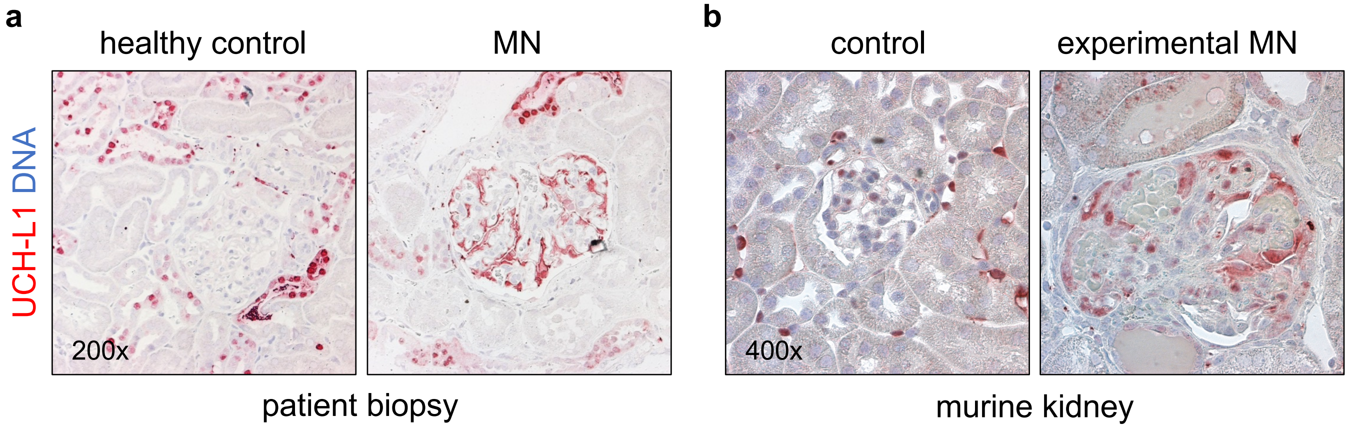

Suppl. Fig. 4: UCH-L1 is de novo expressed in podocytes in human and murine membranous nephropathy. UCH-L1 was stained using a monoclonal antibody to UCH-L1 (13C4, abcam; red) in paraffin sections of (**a**) a patient with membranous nephropathy (MN) and a healthy control or (**b**) a sheep IgG injected control mouse and a sheep anti-murine podocyte antibodies (AP-abs) injected experimental MN mouse on day 14 after disease induction. DNA was stained with hematoxylin (blue). Analyses of 2 independent experiments with n=3, respectively.

Supplementary Fig. 5

a

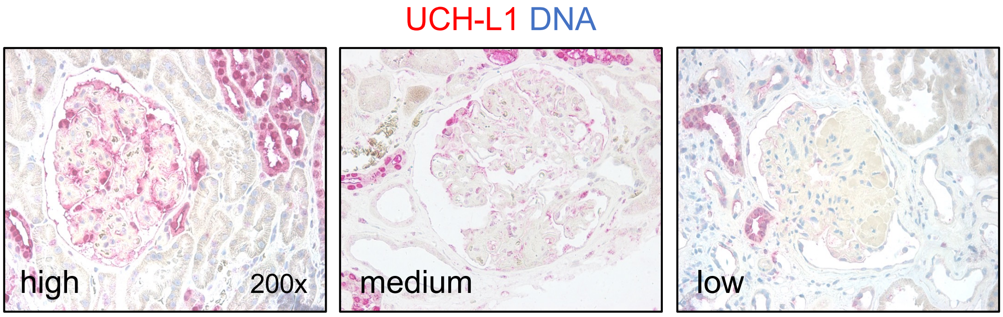

b

UCH-L1 IHC	spontaneous remission	no remission	dialysis
high positivity	n=3	n=3	n=4
medium positivity	n=4	n=3	n=4
low positivity	n=3	n=2	n=1
mean	0,690	0,668	0,747
SEM	0,179	0,233	0,214

spontaneous remission vs. no remission	*p* -value	0,819
spontaneous remission vs. on dialysis	*p* -value	0,538
no remission vs. on dialysis	*p* -value	0,477

c

Comparison MN with low positivity and strong positivity

	p -value
creatinine	0,376
GFR	0,725
proteinuria	0,348
sex	0,604
age	0,464
PLA2R1 titer	0,184
remission of proteinuria achieved	0,250
doubling of creatinine	0,307
tumor	1,000

Suppl. Fig. 5: Expression of UCH-L1 protein in podocytes does not correlate with clinical disease outcome in patients with membranous nephropathy. (**a**) 27 diagnostic kidney biopsies from patients with MN were stained for UCH-L1 (13C4, Abcam; red). DNA was stained with hematoxylin (blue). Podocyte UCH-L1 immunosignal was graded as high-medium-low in a blinded manner in 27 MN biopsies in 1 independent experiment. (**b**) Table summarizing the number of patients with high-medium-low UCH-L1 immunoreactivity in podocytes in the respective clinical outcome groups. The mean +/-SEM indicates the amount of podocyte UCH-L1 immunoreactivity in the scored glomeruli of the three clinical outcome groups. The lower table indicates the statistical analyses of podocyte UCH-L1 expression in relation to clinical outcome, two-tailed Unpaired t-test. (**c**) Subgroup analyses comparing MN patient biopsies with low UCH-L1 immunosignal in podocytes to patient biopsies with high immunosignal to UCH-L1 with the patient's clinical parameters from the time of diagnosis and endpoint, two-tailed Unpaired t-test and Fisher's exact tests.

Supplementary Fig. 6

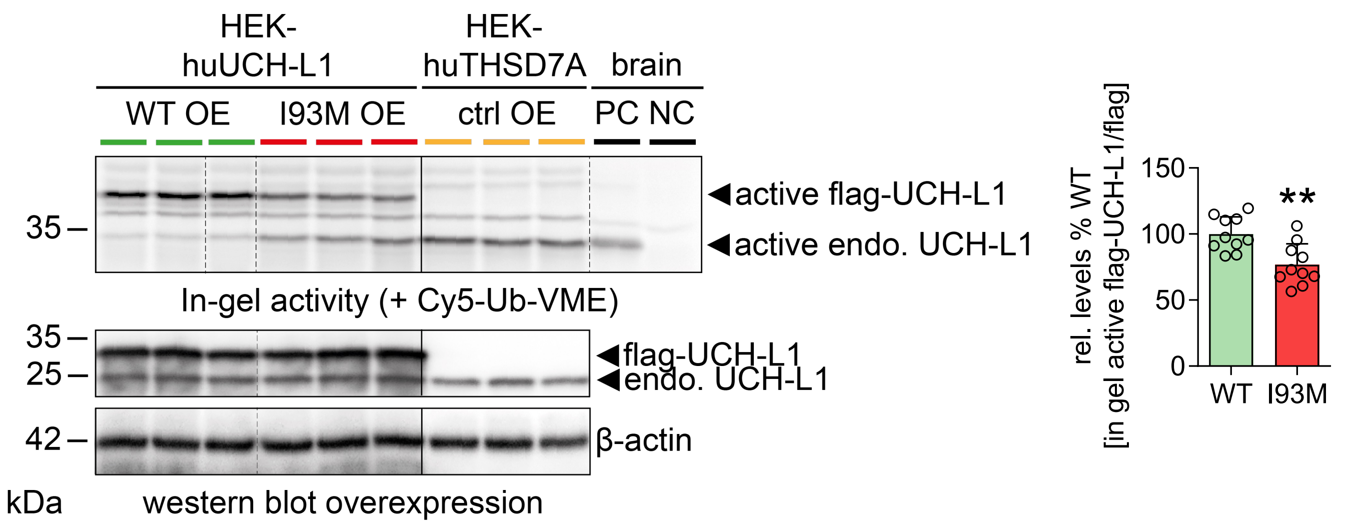

Suppl. Fig. 6: I93M-UCH-L1 but not WT-UCH-L1 exhibits reduced hydrolytic activity. HEK293T cells were transiently transfected with human (hu) wildtype UCH-L1-flag, hu-I93M-UCH-L1-flag or as negative control with human THSD7A-flag. Upper panel: Enzymatic in-gel activity of UCH-L1 was measured using a ubiquitin-based activity probe (Cy5-Ub-VME), which irreversibly binds to the catalytic center of active deubiquitinating enzymes, including UCH-L1. Binding of Cy5-Ub-VME to the active center of UCH-L1 induces a molecular shift of the enzyme (~25 kDa unbound endogenous UCH-L1, ~28 kDa unbound UCH-L1-flag constructs to ~35 kDa bound endogenous UCH-L1 and ~38 kDa bound UCH-L1-flag constructs, respectively). Brain lysates of *Uchl1*$^{-/-}$ mice were used as negative control (NC) and *Uchl1*$^{+/+}$ mice as positive control (PC). Levels of active UCH-L1-flag constructs were quantified by measuring in-gel fluorescence of labeled probe at ~38 kDa subsequently normalized to respective total UCH-L1-flag protein levels. Pooled values of 2 independent experiments are presented as mean +/-SEM, n=10, ***p* = 0.0029, two-tailed Mann Whitney U test. Lower panel exhibits immunoblot for UCH-L1 to show transfection efficiency of UCH-L1-flag constructs. Endo. UCH-L1 = endogenous HEK293T intrinsic UCH-L1. Note the by nearly 25% decreased in-gel hydrolytic activity of I93M-UCH-L1 in comparison to WT-UCH-L1. Interestingly, endogenous HEK293T UCH-L1 shows a contra regulatory increase in activity, depending on the amount of UCH-L1 activity arising from the transfected UCH-L1 constructs (highest in huTHSD7A control (ctrl) transfected overexpression (OE)).

Supplementary Fig. 7

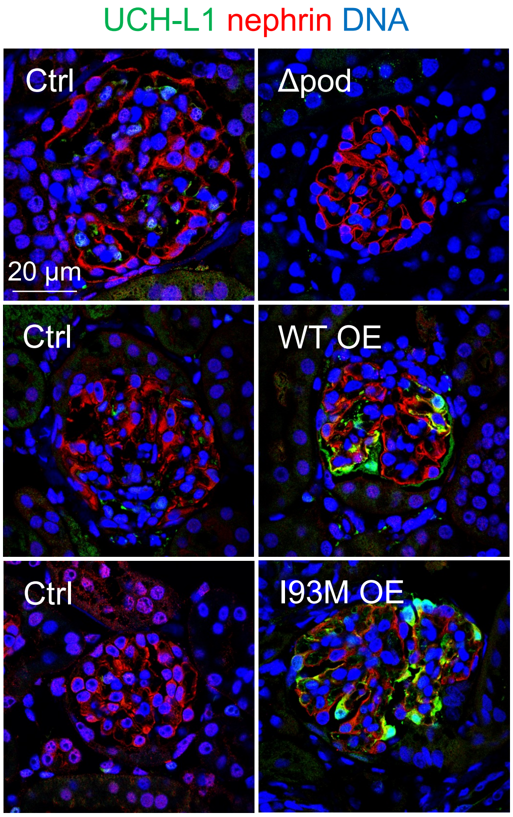

Suppl. Fig. 7: Mouse models of genetic modulation of UCH-L1 expression in podocytes. Transgenic mouse models of modified UCH-L1 expression in podocytes were generated using the Cre-*LoxP* system. By breeding mice with podocin (*Nphs2*)-driven *Cre*-expression with founder mice having 1) lox*P* flanked alleles of UCH-L1 (promoter/ex3) the excision of floxed DNA cause an UCH-L1 knockdown only in podocytes (Δpod). In contrast the *Cre*-driven excision of *loxP* flanked stop cassettes mediates the podocyte-specific overexpression of introduced downstream genes 2) UCH-L1^{WT} (WT OE) and 3) UCH-L1^{I93M} (I93M OE), respectively. Representative confocal images of mouse kidney slices verify UCH-L1 knockdown/overexpression (green) in podocytes. Nephrin (red) was used to depict the glomerular filtration barrier and DNA was stained with Hoechst (blue). Analyses of 2 independent experiments with n=3, respectively.

Supplementary Fig. 8

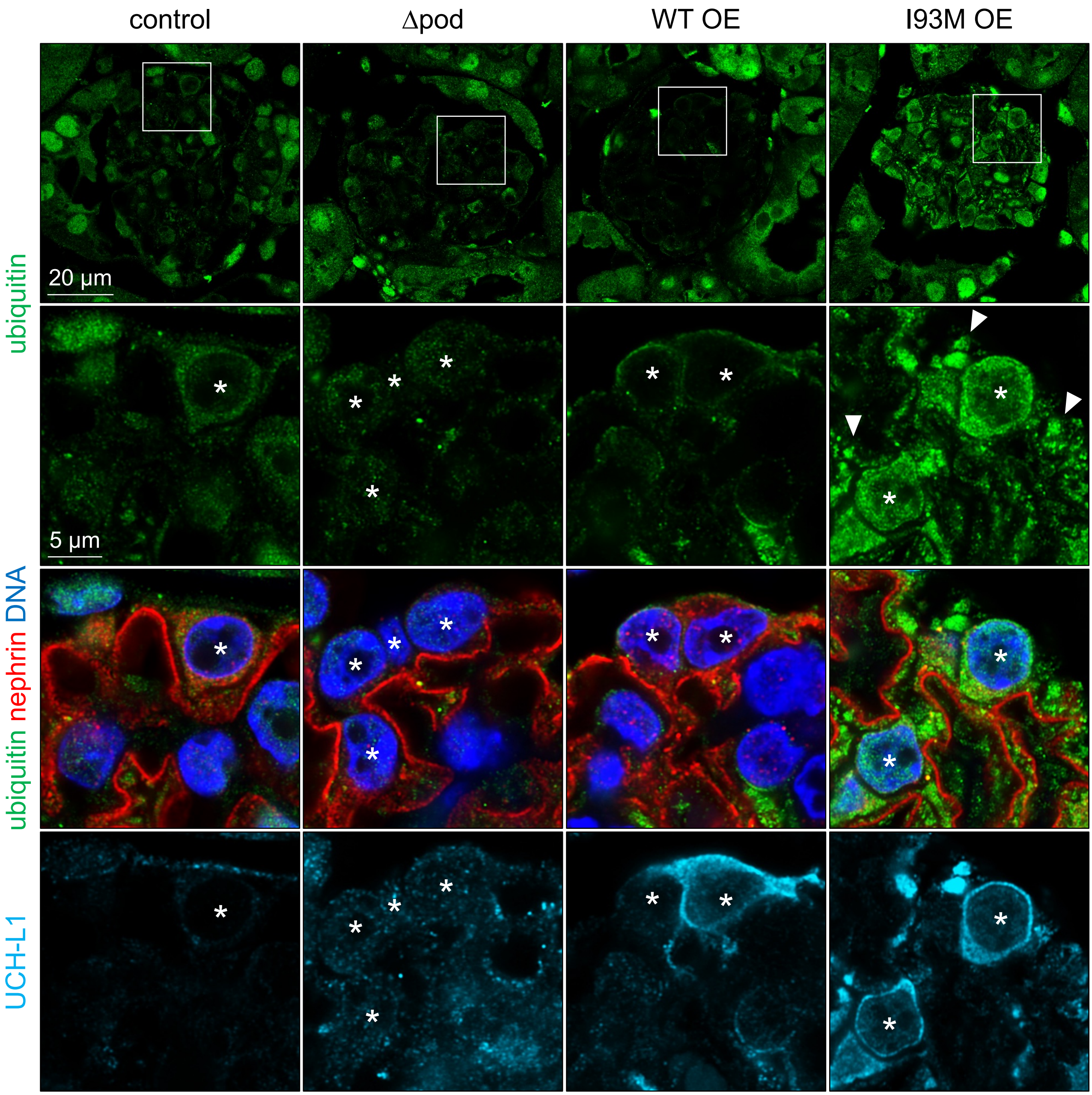

Suppl. Fig. 8: I93M-UCH-L1 but not WT-UCH-L1 exhibit ubiquitin accumulations in podocytes. Representative staining of ubiquitinated proteins (green) in relation to UCH-L1 expression (turquoise) in naive transgenic mice. Note the strong ubiquitin accumulations in I93M-UCH-L1-overexpressing podocytes. Nephrin (red) was used to depict the glomerular filtration barrier and DNA was stained with Hoechst (blue). Asterisks = podocyte nuclei, white arrowheads = granular ubiquitin accumulations. Analyses of 2 independent experiments with n=3, respectively.

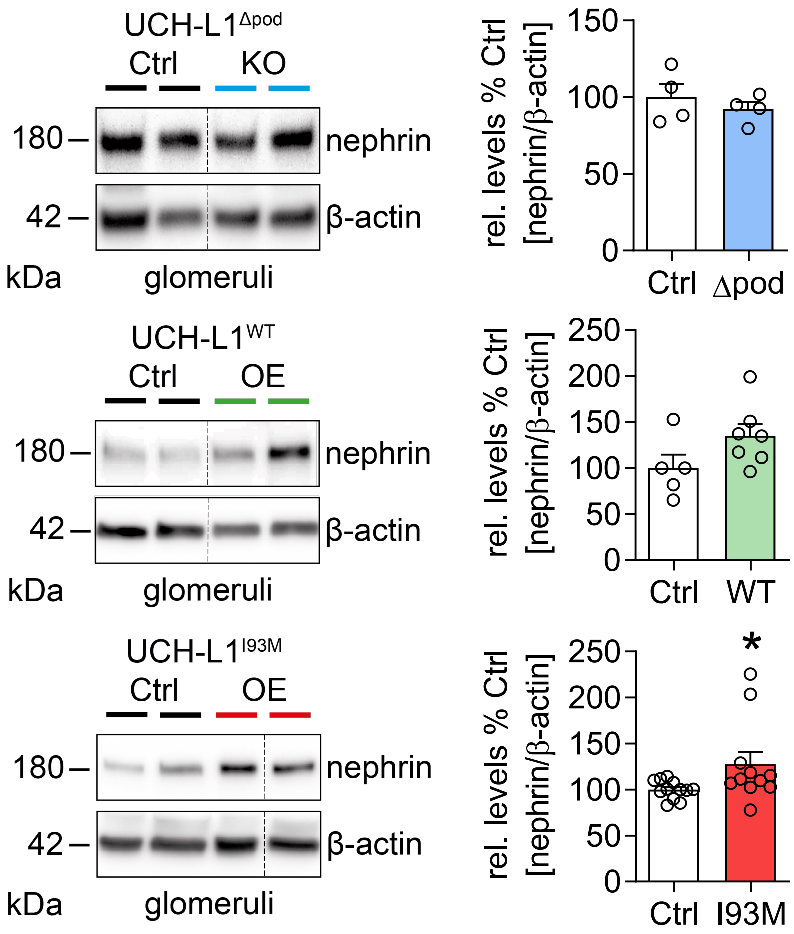

Suppl. Fig. 9: Podocytes of UCH-L1 overexpressing but not UCH-L1 knockdown mice exhibit accumulations of slit diaphragm protein nephrin. Immunoblot analyses for nephrin were performed in glomeruli from naive transgenic mice. Graphs show relative nephrin abundance (normalized to β-actin) to respective littermate controls (Ctrl). Pooled values of 1 independent experiment (UCH-L1 KO, n=4 Ctrl or n=4 Δpod), 3 independent experiments (WT-UCH-L1, n=5 Ctrl or n=7 WT OE) or of 5 independent experiments (I93M-UCH-L1, n=12 Ctrl or n=11 I93M OE), mean +/-SEM, *p = 0.0225, two-tailed Mann Whitney U test.

Supplementary Fig. 10

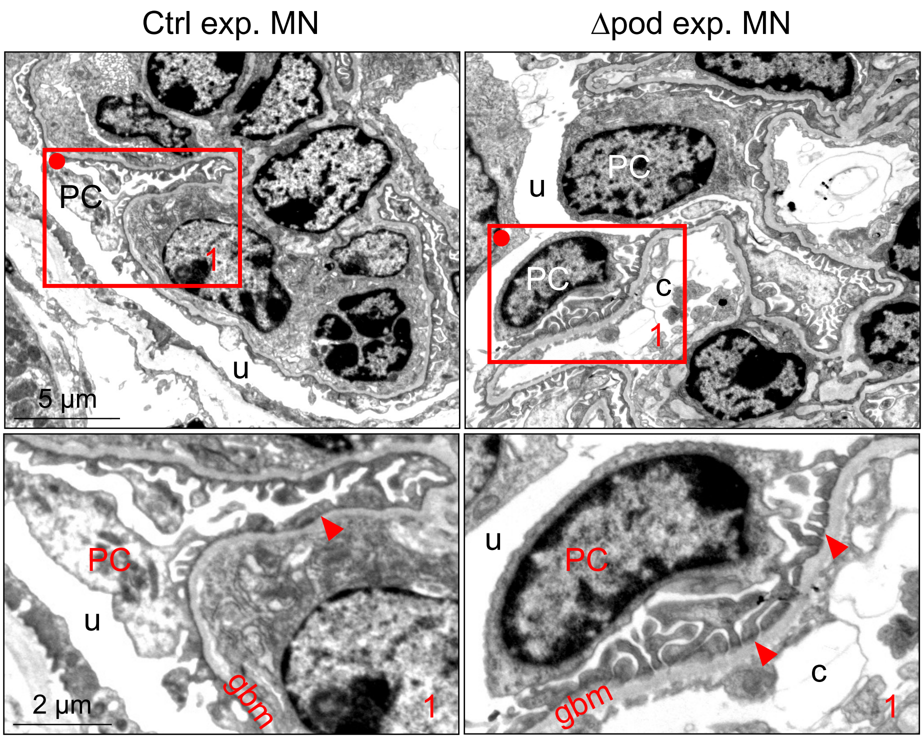

Suppl. Fig. 10: Glomerular filtration barrier alterations are attenuated in UCH-L1Δpod mice in experimental membranous nephropathy. Experimental MN was induced by i.v. injection of sheep anti-murine podocyte antibodies (AP-abs) in podocyte-specific UCH-L1-deficient (Δpod) and littermate (Ctrl) mice. Kidneys were collected on day 14. Ultrastructural electron microscopical (EM) analyses of glomerular basement integrity exhibits effaced foot processes in Ctrl and mostly preserved foot processes in Δpod mice (panel 1). Analyses of 1 independent experiment with n=3, respectively. PC = podocyte, c = capillary lumen, u = urinary space, gbm = glomerular basement membrane, red arrowheads = foot processes.

Supplementary Fig. 11

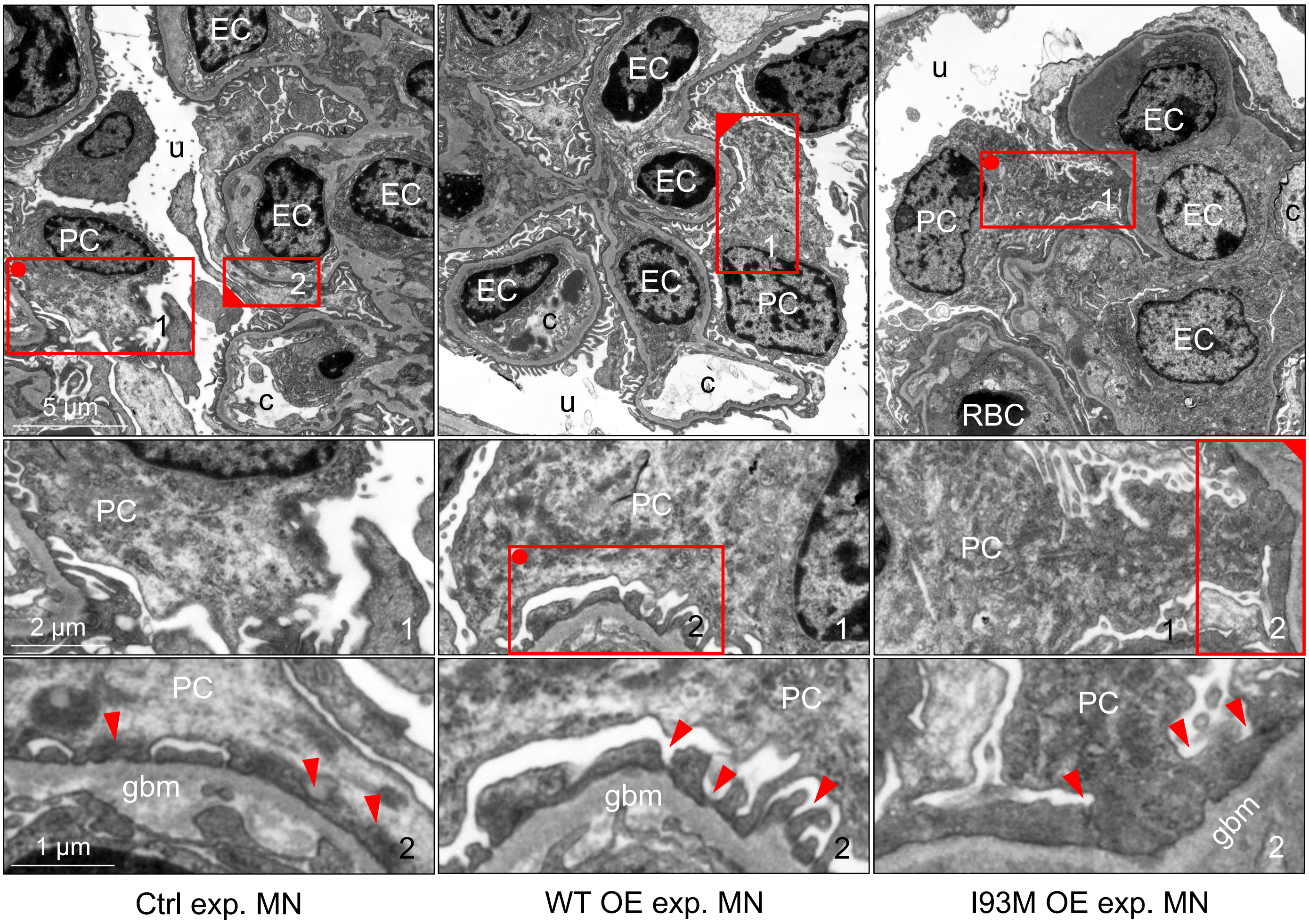

Suppl. Fig. 11: I93M-UCH-L1 but not WT-UCH-L1 mice exhibit severe foot process effacement in experimental membranous nephropathy. Experimental MN was induced by i.v. injection of sheep anti-murine podocyte antibodies (AP-abs) in podocyte-specific WT-UCH-L1 and I93M-UCH-L1 overexpressing (OE), and littermate (Ctrl) mice. Kidneys were collected on day 14 for ultrastructural electron microscopical (EM) analyses. Panel 1 illustrate representative podocyte with nucleus section and cytoplasm with organelles. Lower panels (panel 2) depict severely effaced foot processes in I93M OE mice in comparison to the mildly effaced foot processes in WT-UCH-L1 OE and in Ctrl littermates (red arrowheads). Analyses of 1 independent experiment with n=3, respectively. PC = podocyte, EC = endothelial cell, RBC = red blood cell, c = capillary lumen, u = urinary space, gbm = glomerular basement membrane, red arrowheads = foot processes.

Supplementary Fig. 12

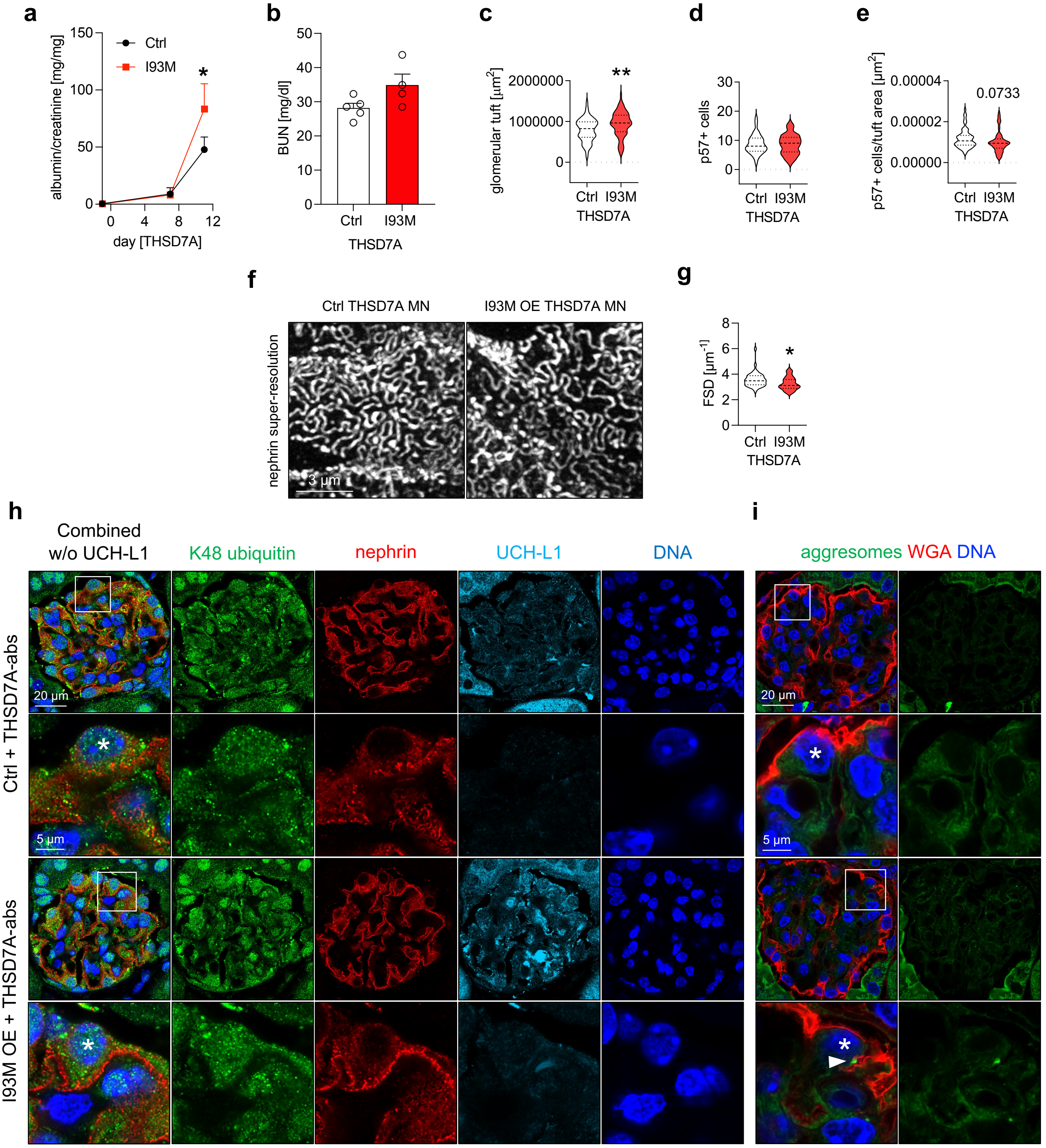

Suppl. Fig. 12: Overexpression of I93M-UCH-L1 aggravates podocyte injury in the THSD7A-associated model of membranous nephropathy (MN). I93M-UCH-L1 overexpressing mice (backcrossed for 3 generations from the C57BL/6J background with BALB/c to a mixed background) and their respective littermates (Ctrl) were injected with rabbit anti-THSD7A IgG for the induction of THSD7A-associated MN. Mice were analyzed on day 12 after disease induction. Data from 1 experiment with n=5 (Ctrl) or n=4 (I93M OE) are shown. (**a**) Urinary albumin to creatinine ratio, mean +/-SEM, **p* = 0.0188, Two-Way ANOVA to assess glomerular filtration barrier functionality. (**b**) Blood urea nitrogen (BUN) to evaluate renal function, mean +/-SEM. (**c-e**) Quantification of podocyte loss by determination of glomerular tuft area (**c**), the corresponding number of stained p57-positive podocytes

per glomerulus (**d**) and ratio of both (**e**), pooled values from n=60 (Ctrl) or n=60 (I93M OE) glomeruli, mean +/-SEM, ***p* = 0.0085, two-tailed Unpaired t-test. (**f**) Super-resolution analyses of nephrin meanders to visualize podocyte foot process effacement. (**g**) Quantification of filtration slit density (FSD) length per area (μm^{-1}) using the podocyte exact morphology procedure (PEMP) to quantify the extent of foot process effacement, pooled values from n=41 (Ctrl) or n=28 (I93M OE), mean +/-SEM, **p* = 0.0151, two-tailed Mann Whitney U test. (**h**) Confocal images of K48-ubiquitinated proteins (green) in affected podocytes (marked by asterisks). Nephrin (red) was used to depict the glomerular filtration barrier. UCH-L1 (turquoise) was stained to illustrate overexpressing podocytes. DNA was stained with Hoechst (blue). (**i**) Confocal images of inclusion bodies (aggresomes, marked by white arrowhead) in I93M OE podocyte (marked by asterisk). Wheat germ agglutinin (WGA, red) highlights the podocyte plasma membrane. DNA was stained with Hoechst (blue). Analyses of 2 independent experiments with n=3, respectively.

Supplementary Fig. 13

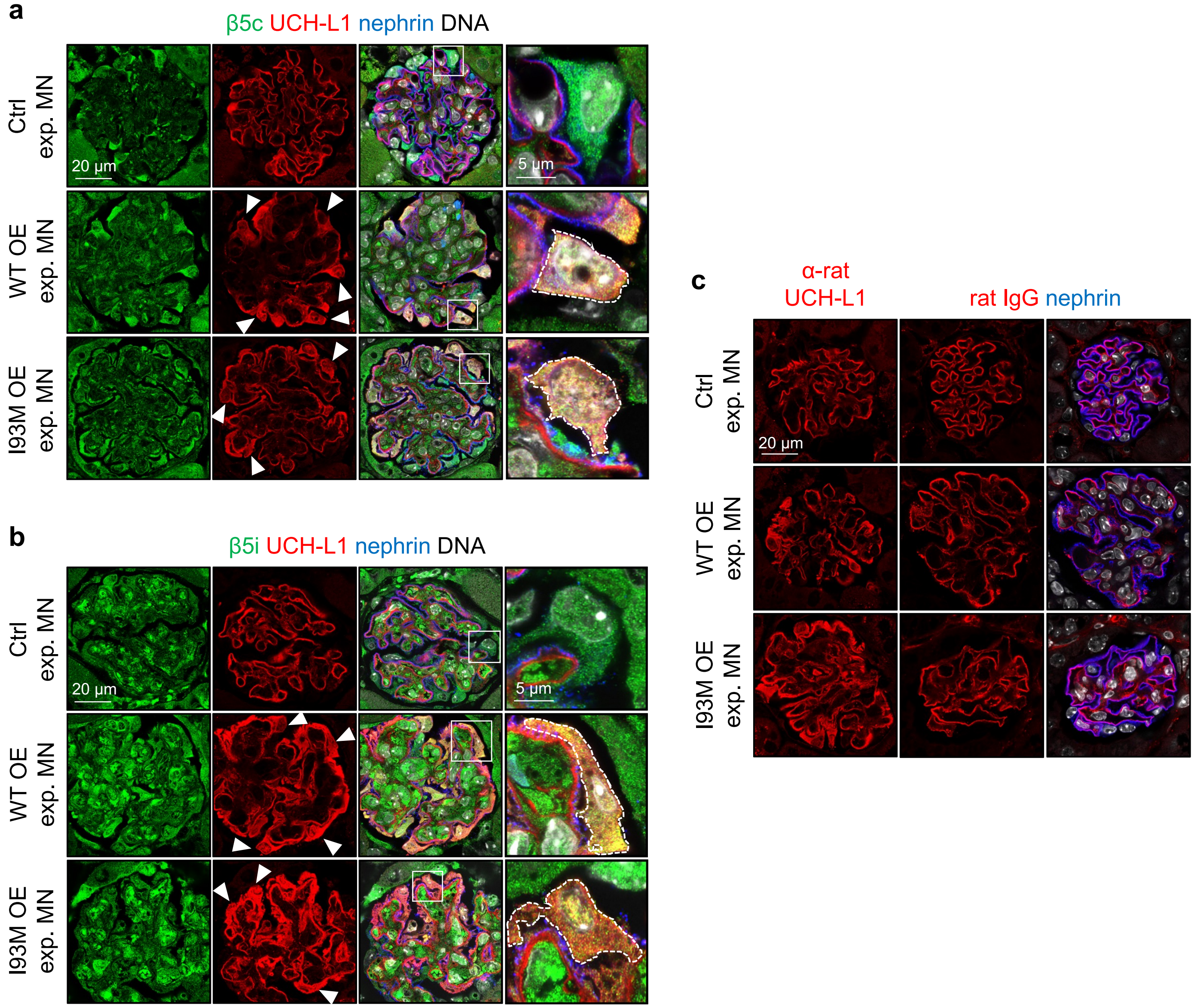

Suppl. Fig. 13**: **The proteolytic β5c and β5i subunits of the proteasome co-localize with UCH-L1 in podocytes in experimental MN. Experimental MN was induced by i.v. injection of sheep anti-murine podocyte antibodies (AP-abs) in UCH-L1 overexpressing (WT OE and I93M OE, respectively) and their relative littermate (Ctrl) mice. Kidneys were collected on day 14. Representative confocal images showing co-localization of the (**a**) β5c and (**b**) β5i proteolytic proteasomal subunits (green) with UCH-L1 (red) in podocytes. Nephrin (blue) was used to depict the glomerular filtration barrier and DNA was stained with Hoechst (grey). Yellow: Spatial proximity of β5c and β5i with UCH-L1 in podocytes. White arrowheads highlight podocytes with cytoplasmic UCH-L1 expression. Analyses of 2 independent experiments with n=3, respectively. (**c**) Note the additional linear red signal arising from the deposited mouse IgG, which is unspecifically detected by the rat anti-UCH-L1 monoclonal antibody used for staining. Nephrin (blue) was used to depict the glomerular filtration barrier. Analyses of 2 independent experiments with n=3, respectively.

Supplementary Fig. 14

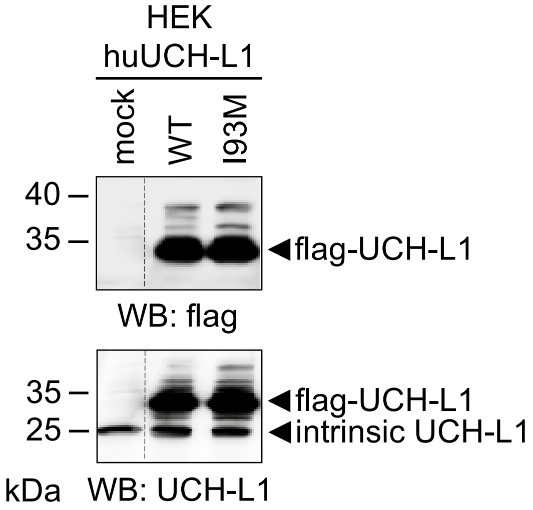

Suppl. Fig. 14**: **Representative immunoblots depicting transfection efficiency of transiently transfected HEK293T cells used for proteasomal interaction studies in Fig. 6d. HEK293T cells were transiently transfected with WT-UCH-L1 (human (hu) wildtype UCH-L1-flag) and I93M-UCH-L1 (hu-I93M-UCH-L1-flag). Representative immunoblots analyses of 1 independent experiment against flag (upper panel) and UCH-L1 (lower panel) depict equal expression of I93M- and WT-UCH-L1 constructs. Intrinsic UCH-L1 = UCH-L1 expressed by HEK293T cells.

Supplementary Fig. 15

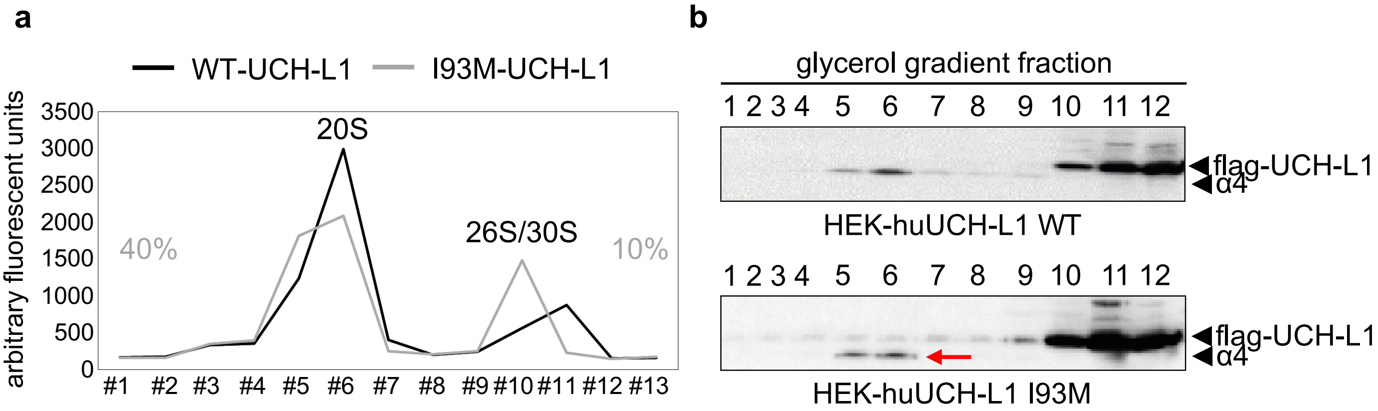

Suppl. Fig. 15**: **I93M-UCH-L1 and WT-UCH-L1 interact with the proteasome. Glycerol gradient fractionation (10%-40% glycerol) of cell lysates of HEK293T cells expressing huUCH-L1 WT or huUCH-L1 I93M. (**a**) Gradient fractions were measured for the chymotryptic peptide-hydrolyzing activity of proteasomes. The peaks for proteasome complexes are indicated (#4-7 20S proteasome core complexes; #9-12 26S/30S proteasome complexes). (**b**) Western blot analysis of the glycerol gradient fractions stained for UCH-L1 and α4. UCH-L1-I93M-flag signal co-migrates more pronounced with 20S proteasome fractions (red arrow). Analyses of 1 independent experiment.

Supplementary Fig. 16

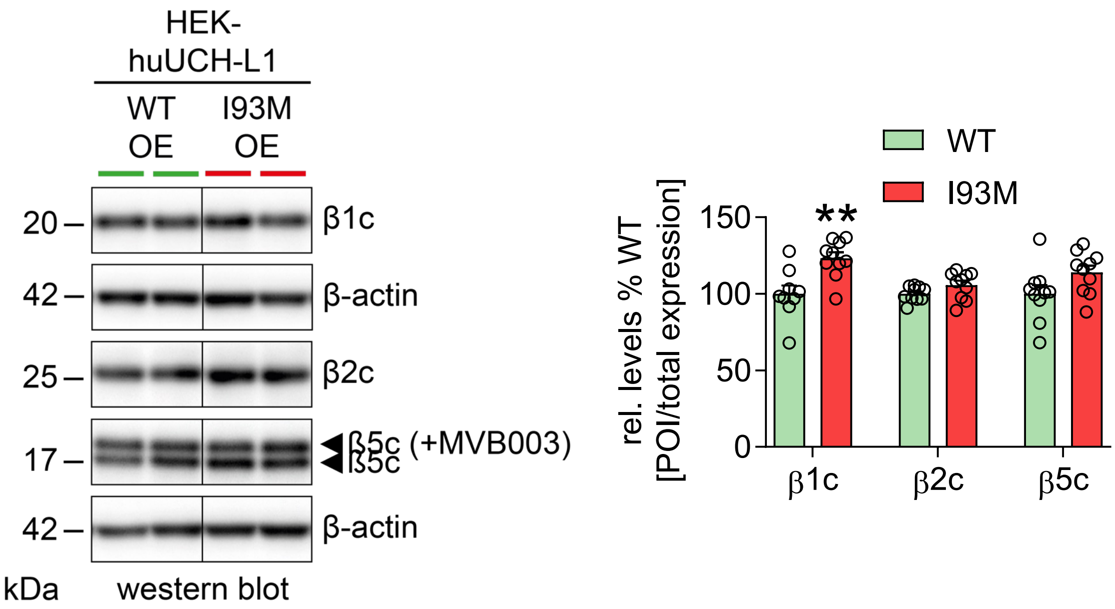

Suppl. Fig. 16**: **Protein abundance of β1c, β2c and β5c in HEK293T cells transiently transfected with wildtype or I93M-UCH-L1. Quantification of proteasome β-subunit abundance in HEK293T cells transiently transfected with human (hu) wildtype UCH-L1-flag (WT-UCH-L1) and hu-I93M-UCH-L1-flag (I93M-UCH-L1) protein. The depicted representative immunoblots to β1c, β2c, and β5c normalized to β-actin of the respective membrane were generated by blotting the gel from an in-gel subunit activity assay. MVB003 = pan proteasomal activity-based probe, which binds covalently to active subunits, hence inducing a small molecular shift. Graph depicts pooled relative values to WT OE transfected HEK293T cells of 2 independent experiments expressed as mean +/-SEM, n=9 (β1c WT OE) or n=10 for residual groups, ***p* = 0.0076, two-tailed Mann Whitney U test.

Supplementary Fig. 17

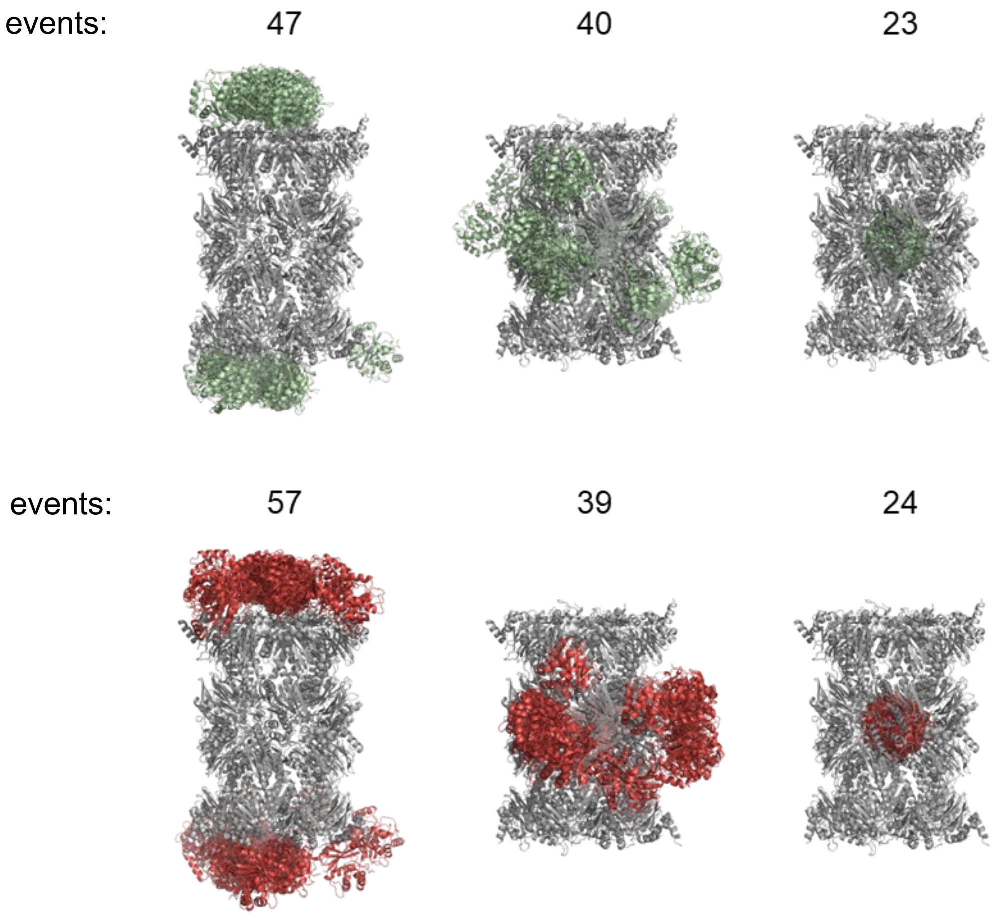

Suppl. Fig. 17**: **In silico docking predictions based on electrostatic potential of UCH-L1. Predicted binding configurations of hu-WT-UCH-L1 (green) and hu-I93M variant (red) to the human 20S proteasome (grey) as calculated using Cluspro (https://cluspro.bu.edu). Shown are the number and the configuration of predicted binding events classified by binding sites. Side views of identical orientations of the 20S proteasome (PDB 5LE5) are shown in grey cartoon representation. UCH-L1 molecules are shown in all predicted binding configuration in green (UCH-L1 wildtype) and in red (I93M-UCHL1 variant) in cartoon representation. The predicted binding sites are classified as potential binding to the proteasomal alpha subunits (left panel), to the proteasomal beta subunits (middle panel) or to the internal proteasomal cavity (right panel). As binding of natively folded UCH-L1 molecules to the internal cavity is not of any biological relevance these cases are not further analyzed. The number of predicted binding events to the cavity are subtracted from the total number of calculated events, which is 110 in the case of the WT-UCH-L1 and 120 in the case of the I93M-UCH-L1 variant. Considering only binding events of biological relevance, binding of WT-UCH-L1 occurs almost equally likely to alpha and to beta subunits with a ratio 54% to 46%. In comparison there is a slight increase in the likelihood of binding of the I93M-UCH-L1 variant to the alpha subunits compared to the beta subunits with a ratio of 59% to 41%.

Uncropped gels and blots

Supplementary Fig. 3a

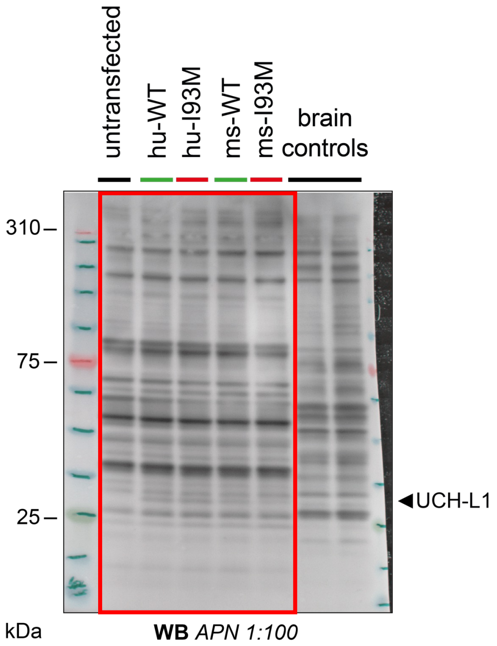

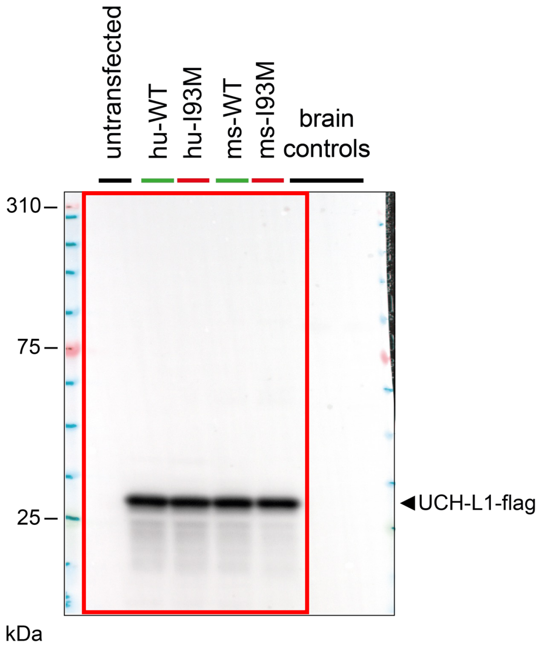

antibodies:
anti-goat-HRP 1:10000
mouse anti-FLAG (clone M2, Sigma-Aldrich), 1:1000 in Superblock

Supplementary Fig. 3b

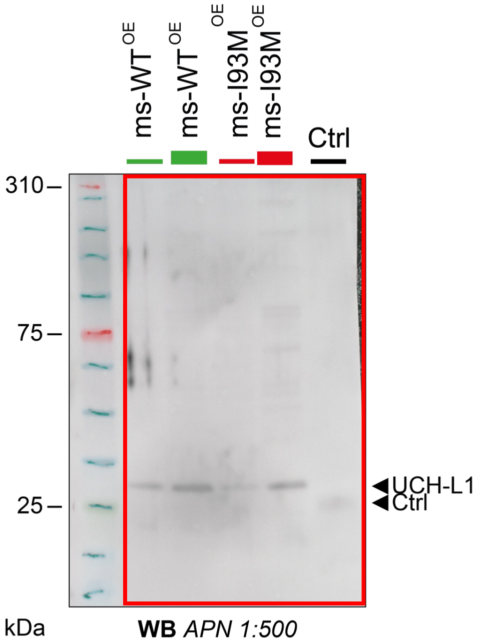

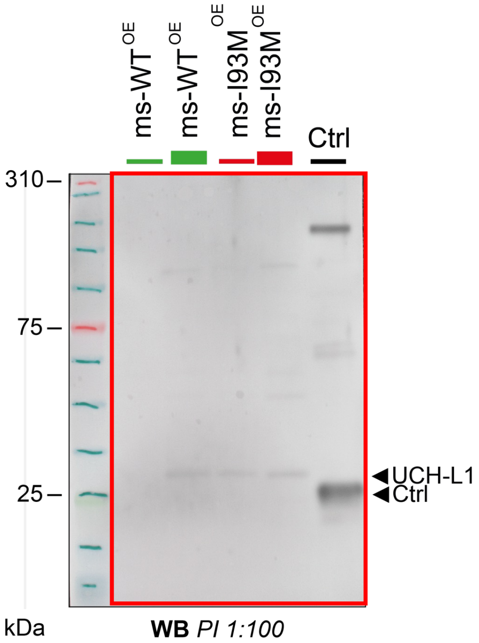

antibodies:
anti-goat-HRP 1:10000

Uncropped gels and blots

Supplementary Fig. 3b

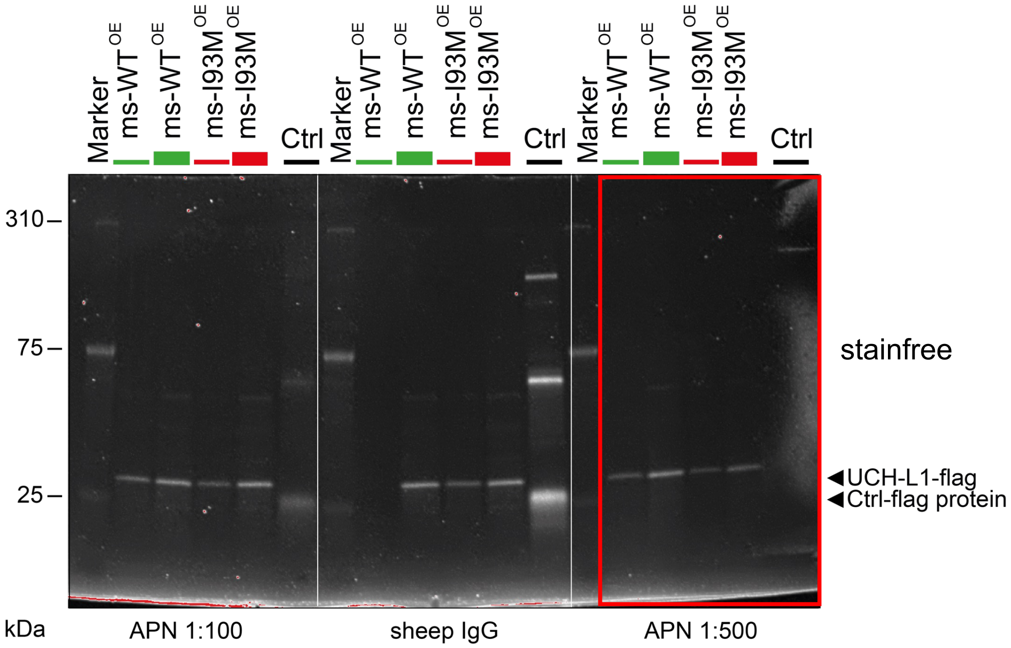

<u>panel:</u>
Stainfree
detected with Tryptophan UV activation, filter F590 (FUSION FX07 (Vilber))

Supplementary Fig. 6

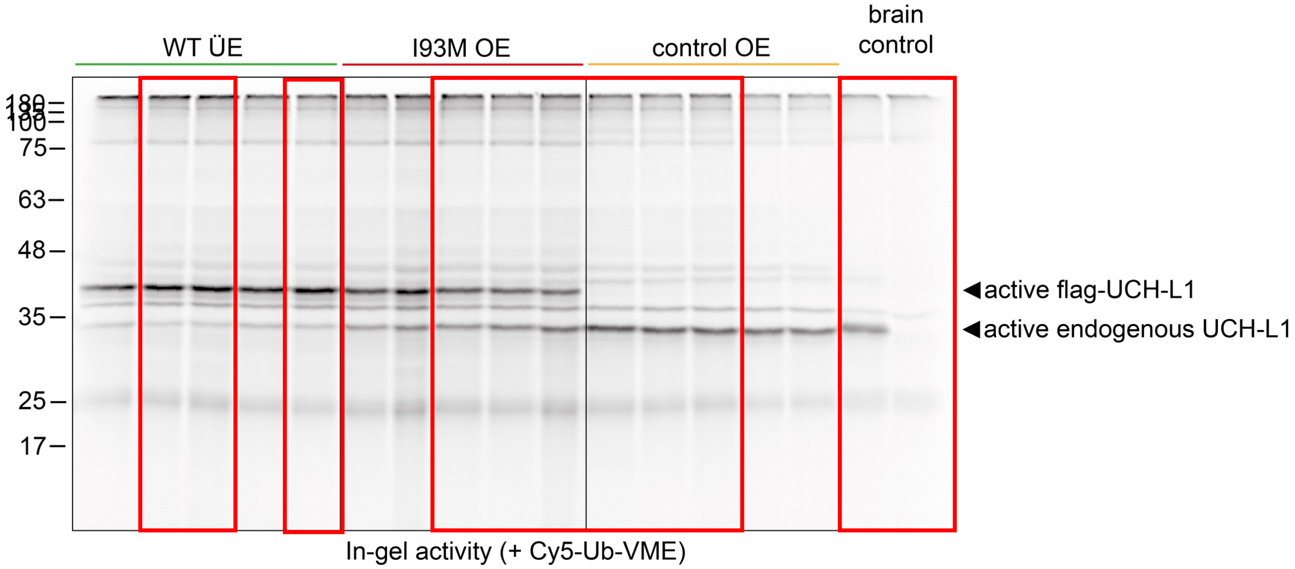

<u>panel:</u>
labelled with Cy5-Ub-VME
detected with light capsule C640, filter F710 (FUSION FX07 (Vilber))

Uncropped gels and blots

Supplementary Fig. 6

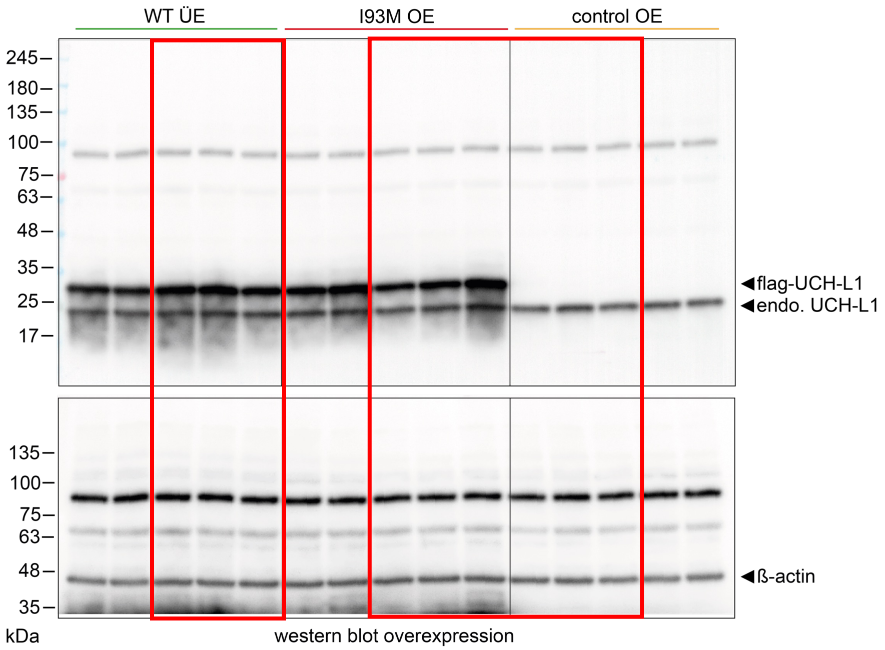

antibodies:
rabbit anti-UCH-L1 (ab27053, Abcam), 1:250 in 3% non-fat milk
mouse anti-β-actin (clone AC-15, Sigma-Aldrich), 1:10000 in Superblock

Supplementary Fig. 9 *(UCH-L1$^{\Delta pod}$ & UCH-L1^{WT})*

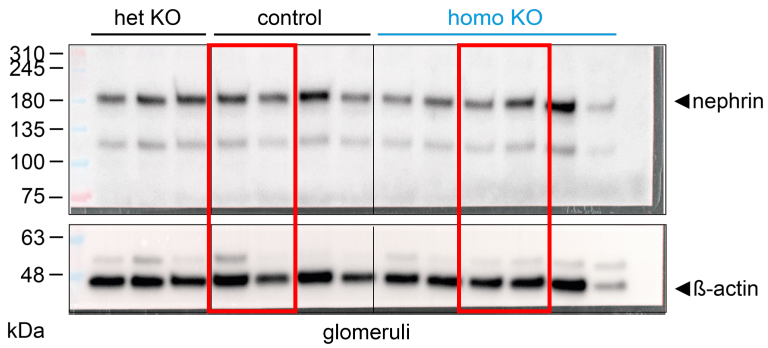

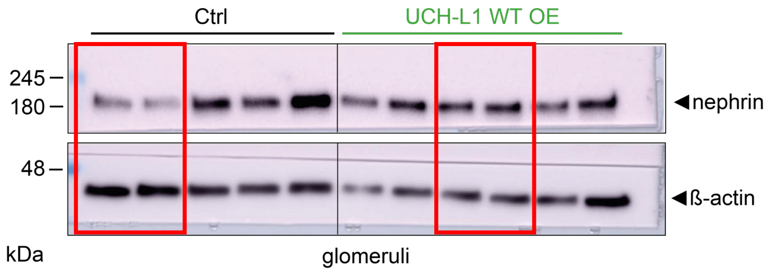

Uncropped gels and blots

Supplementary Fig. 9 *(UCH-L1[I93M])*

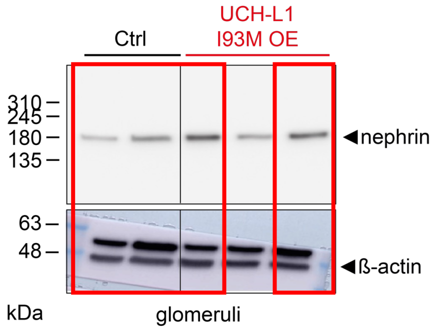

antibodies:
guinea-pig anti-nephrin (GP-N2, Progen), 1:2000 in Superblock
mouse anti-β-actin (clone AC-15, Sigma-Aldrich), 1:10000 in Superblock

Supplementary Fig. 14

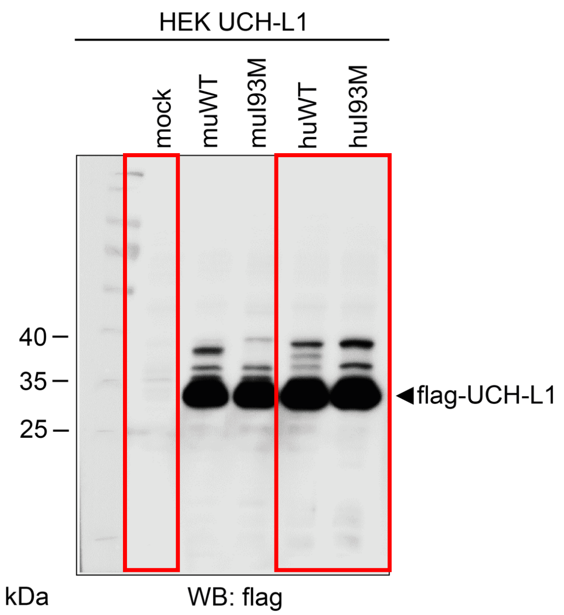

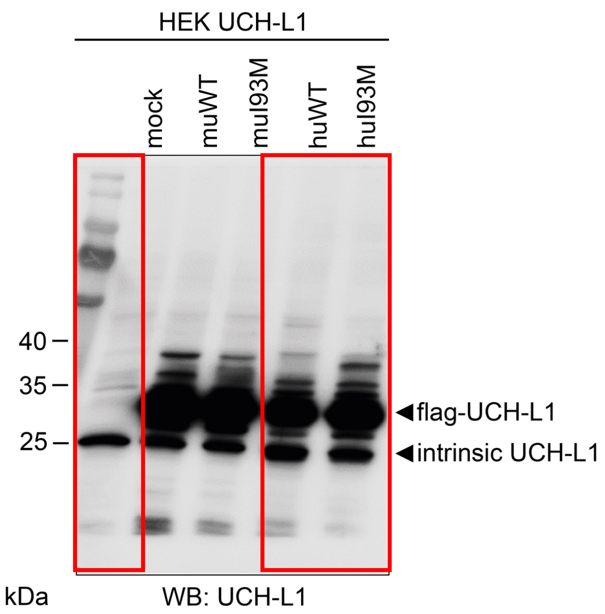

antibodies:
mouse anti-FLAG (clone M2, Sigma-Aldrich), 1:1000 in Superblock
rat anti-UCH-L1 (Sosna *et al.*), 1:250 in 3% non-fat milk

Uncropped gels and blots

Supplementary Fig. 15b

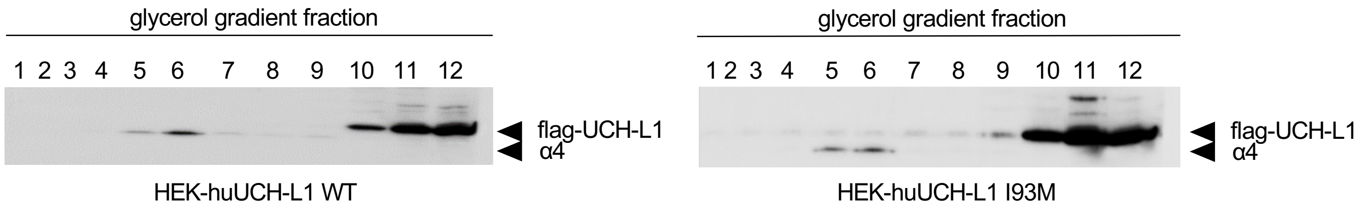

<u>antibodies:</u>
rat anti-UCH-L1 (Sosna *et al.*), 1:250 in 3% non-fat milk
rabbit anti-α4 (laboratory stock), 1:1000 in Superblock

Supplementary Fig. 16

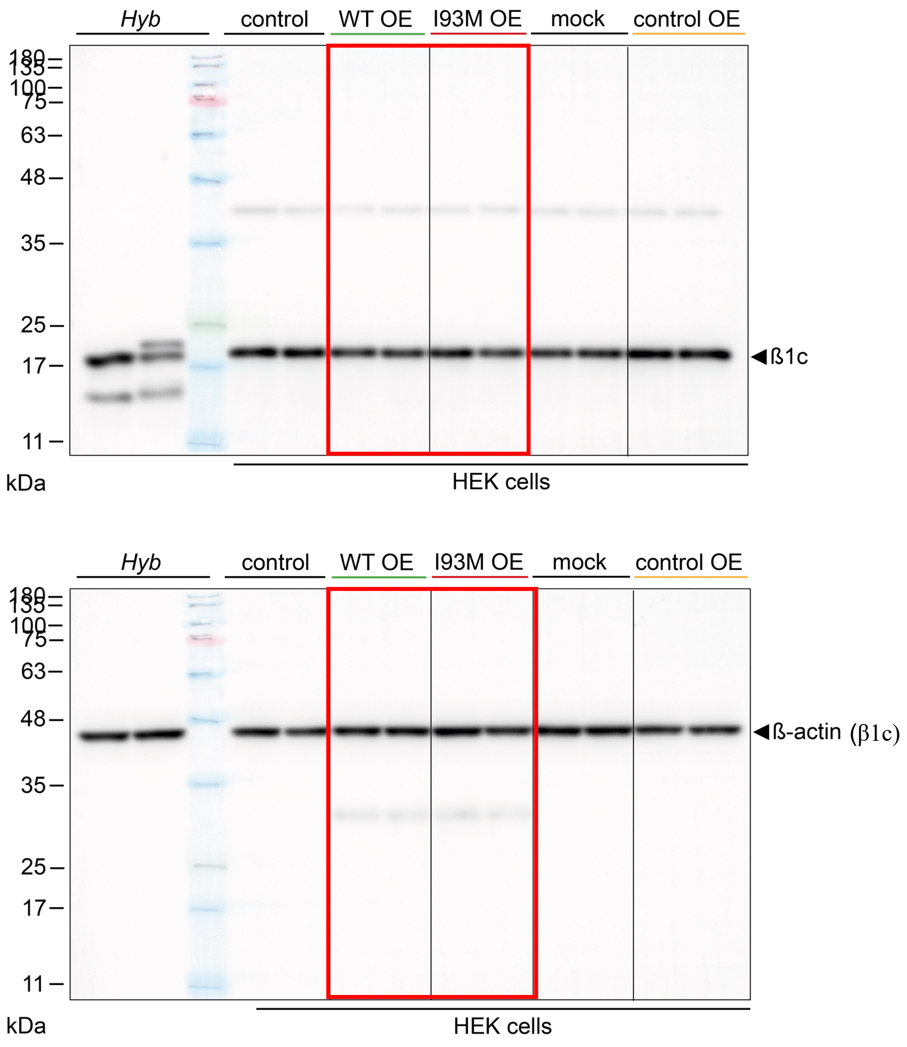

<u>antibodies:</u>
rabbit anti-β1c (PA1-978, Invitrogen), 1:1000 in Superblock
mouse anti-β-actin (clone AC-15, Sigma-Aldrich), 1:10000 in Superblock

Uncropped gels and blots

Supplementary Fig. 16

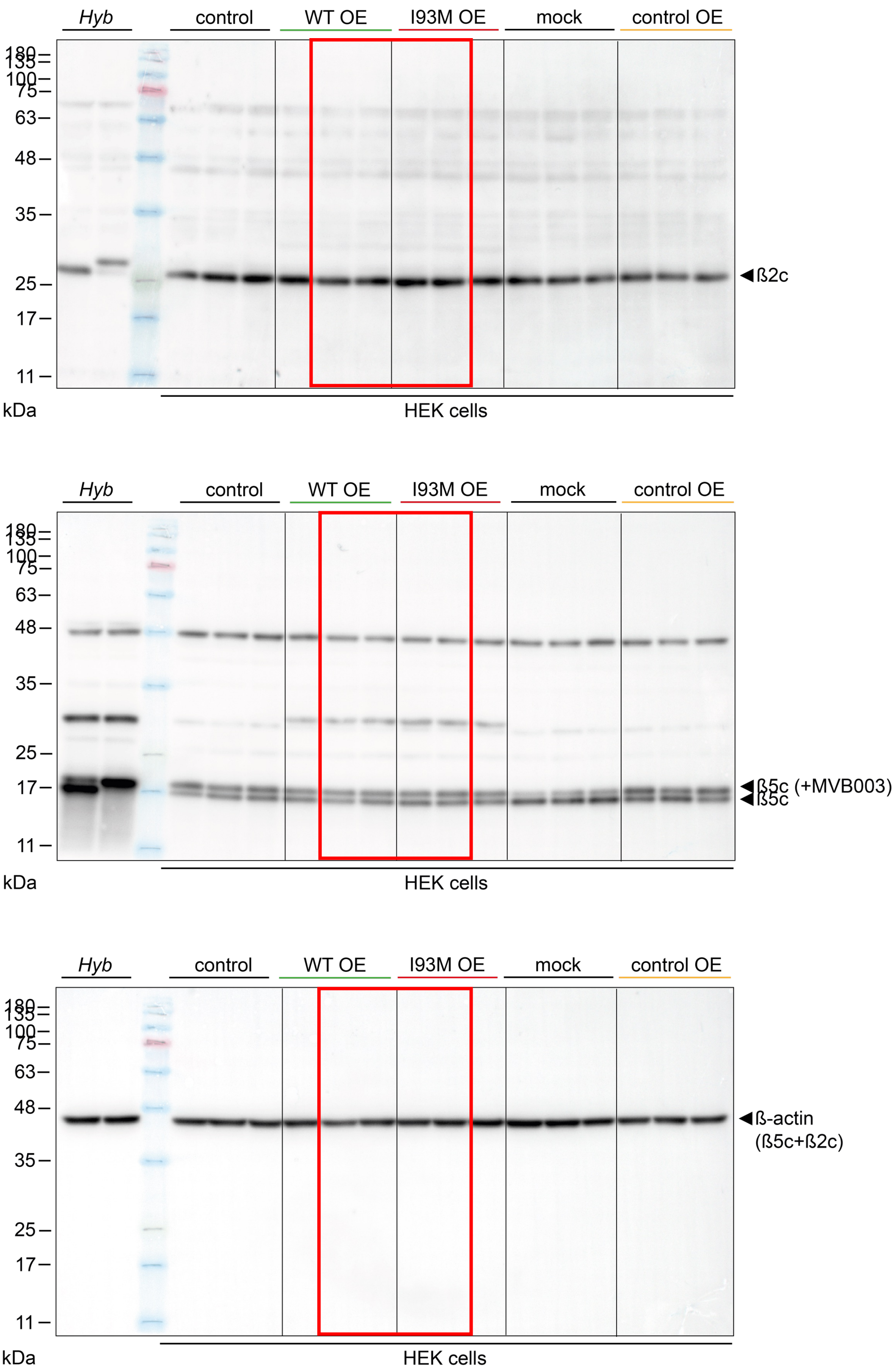

antibodies:
rabbit anti-β2c (PA5-30988, Invitrogen), 1:1000 in Superblock
rabbit anti-β5 (X. Wang, University of South Dakota, USA), 1:5000 in Superblock
mouse anti-β-actin (clone AC-15, Sigma-Aldrich), 1:10000 in Superblock
