# Supplementary material for: Non-functional ubiquitin C-terminal hydrolase L1 drives podocyte injury through impairing proteasomes in autoimmune glomerulonephritis
Source: Nat Commun. 2023 Apr 13;14:2114. doi: 10.1038/s41467-023-37836-8 (PMC10102022; doi:10.1038/s41467-023-37836-8)
Supplement: Supplementary file 2 — Reporting Summary [file 41467_2023_37836_MOESM2_ESM.pdf]

nature portfolio

Corresponding author(s): Catherine Meyer-Schwesinger

Last updated by author(s): 2023/03/31

Reporting Summary

Nature Portfolio wishes to improve the reproducibility of the work that we publish. This form provides structure for consistency and transparency in reporting. For further information on Nature Portfolio policies, see our Editorial Policies and the Editorial Policy Checklist.

Statistics

For all statistical analyses, confirm that the following items are present in the figure legend, table legend, main text, or Methods section.

n/a	Confirmed	
☐	☒	The exact sample size (*n*) for each experimental group/condition, given as a discrete number and unit of measurement
☐	☒	A statement on whether measurements were taken from distinct samples or whether the same sample was measured repeatedly
☐	☒	The statistical test(s) used AND whether they are one- or two-sided<br>*Only common tests should be described solely by name; describe more complex techniques in the Methods section.*
☒	☐	A description of all covariates tested
☒	☐	A description of any assumptions or corrections, such as tests of normality and adjustment for multiple comparisons
☐	☒	A full description of the statistical parameters including central tendency (e.g. means) or other basic estimates (e.g. regression coefficient) AND variation (e.g. standard deviation) or associated estimates of uncertainty (e.g. confidence intervals)
☐	☒	For null hypothesis testing, the test statistic (e.g. *F*, *t*, *r*) with confidence intervals, effect sizes, degrees of freedom and *P* value noted<br>*Give P values as exact values whenever suitable.*
☒	☐	For Bayesian analysis, information on the choice of priors and Markov chain Monte Carlo settings
☒	☐	For hierarchical and complex designs, identification of the appropriate level for tests and full reporting of outcomes
☐	☒	Estimates of effect sizes (e.g. Cohen's *d*, Pearson's *r*), indicating how they were calculated

Our web collection on statistics for biologists contains articles on many of the points above.

Software and code

Policy information about availability of computer code

Data collection	Data collection was performed with<br>Fusion FX7EDGE V0.7 Imager (Vilbert Lourmat)<br>Amersham Imager 600 (GE Healthcare)<br>ZEISS LSM800 with airyscan1 microscope<br>ZEISS LSM510 microscope<br>4 channel STED microscope (Abberior)<br>Elyra PS.1 SIM microscope<br>transmission-electron microscope (TEM 910)<br>QuantStudio 5 Real Time PCR System<br>Mithras LB 940
Data analysis	Data analysis was performed with<br>GraphPad Prism, Version 9.5.0<br>Adobe Photoshop Version 23.0.1<br>Excel Version 16.68<br>Bio 1D Software (Vilbert Lourmat)<br>EvolutionCapt Software (Vilber Lourmat)<br>ZEN 3.6<br>ZEN 3.0

LSM Image Browser
FIJI Version 2.0
PyMOL plugin PyTMs (https://pymol.org/2/)
APBS-PDB2PQR software suite (https://server.poissonboltzmann.org/)
Cluspro (https://cluspro.bu.edu)

For manuscripts utilizing custom algorithms or software that are central to the research but not yet described in published literature, software must be made available to editors and reviewers. We strongly encourage code deposition in a community repository (e.g. GitHub). See the Nature Portfolio guidelines for submitting code & software for further information.

Data

Policy information about availability of data

All manuscripts must include a data availability statement. This statement should provide the following information, where applicable:

- Accession codes, unique identifiers, or web links for publicly available datasets
- A description of any restrictions on data availability
- For clinical datasets or third party data, please ensure that the statement adheres to our policy

Key sources such as genotyping, RT-qPCR primers and antibodies are provided in the method section. All relevant data supporting the key findings of this study are available within the article and its supplementary information files or from the corresponding author upon reasonable request. A reporting summary for this article is available as a supplementary information file. Source data are provided within this paper.

Human research participants

Policy information about studies involving human research participants and Sex and Gender in Research.

Reporting on sex and gender	For the human studies no differences in sex were made.
Population characteristics	The patient cohort comprised 39 patients with the primary diagnosis of membranous nephropathy.<br>10 females and 29 males<br>age 59 +/-2.32 years
Recruitment	Through the Hamburg GN Registry
Ethics oversight	From the Hamburg GN Registry board: Prof. Dr. Wiech, PD Dr. Hoxha, PRof. Dr. T.B. Huber

Note that full information on the approval of the study protocol must also be provided in the manuscript.

Field-specific reporting

Please select the one below that is the best fit for your research. If you are not sure, read the appropriate sections before making your selection.

[x] Life sciences [ ] Behavioural & social sciences [ ] Ecological, evolutionary & environmental sciences

For a reference copy of the document with all sections, see nature.com/documents/nr-reporting-summary-flat.pdf

Life sciences study design

All studies must disclose on these points even when the disclosure is negative.

Sample size	Sample size per experiment was determined depending on<br>1) Experimental feasability<br>2) Mouse breeding- and genotype availability<br>In general, 2-3 independent experiments were pooled to reach statistical power in cases where experimental feasability did not allow experimentation of a sufficient n for statistical power.
Data exclusions	Mice were excluded depending on<br>1) the efficiency of transgene expression and<br>2) tubular contamination of glomerular preparations
Replication	All experiments were independently replicated over 2 times by different researchers and over the course of 15 years. The number of replications for each experiment is indicated in the figure legend
Randomization	allocation to the groups occured based on the genotype

Blinding	Aquisition of histological measurements were performed in a blinded fashion.<br>The aquisition of animal data were performed in a blinded manner, revelation of genotypes occurred after data aquisition.

Reporting for specific materials, systems and methods

We require information from authors about some types of materials, experimental systems and methods used in many studies. Here, indicate whether each material, system or method listed is relevant to your study. If you are not sure if a list item applies to your research, read the appropriate section before selecting a response.

Materials & experimental systems

n/a	Involved in the study
☐	☒ Antibodies
☐	☒ Eukaryotic cell lines
☒	☐ Palaeontology and archaeology
☐	☒ Animals and other organisms
☒	☐ Clinical data
☒	☐ Dual use research of concern

Methods

n/a	Involved in the study
☒	☐ ChIP-seq
☒	☐ Flow cytometry
☒	☐ MRI-based neuroimaging

Antibodies

Antibodies used

Primary antibodies used for the study were: rat anti-UCH-L1 (immunofluorescence microscopy mouse (IF) 1:50, immunoblot (WB) 1:250, Prof. Grötzinger, CAU Kiel, Sosna et al.(1)); mouse anti-UCH-L1 (IF human kidney 1:50, TSA-amplification, clone 13C4, #ab8189, Abcam); rabbit anti-UCH-L1 (WB 1:250, #ab27053, Abcam); rabbit anti-SOD2 (IF 1:200, #TA326596, Origene); mouse anti-FLAG (WB 1:1000, clone M2, #F3165, Sigma-Aldrich); rabbit anti-alpha2 (WB 1:500, #2455, Cell Signaling); rabbit anti-alpha4 (WB 1:1000, laboratory stock, Prof. E. Krüger, Biochemistry Greifswald); mouse anti-alpha6 (WB 1:1000, clone MCP20, #BML-PW8100, Enzo); rabbit anti-beta1c (WB 1:1000, #PA1-978, Invitrogen); rabbit anti-beta2c (WB 1:1000, #PA5-30988, Invitrogen); rabbit anti-beta5c (WB 1:1000, #PA1-977, Invitrogen); rabbit anti-beta5c (IF 1:300, WB 1:5000, laboratory stock, Prof. X. Wang, University of South Dakota, USA); rabbit anti-beta5i (IF, 1:300, WB 1:5000, laboratory stock, Prof. E. Krüger, Biochemistry Greifswald); rabbit anti-ubiquitin (IF 1:300, #NB300-129, Novus); mouse anti-ubiquitin (WB 1:250, clone Ubi-1, #MAB1510, Millipore); rabbit anti-K48-polyubiquitin (IF 1:300, #ab140601, Abcam); rabbit anti-K48-polyubiquitin (WB 1:1000, clone Apu2, #05-1307, Millipore); guinea-pig anti-nephrin (IF 1:200, WB 1:2000, #GP-N2, Progen); rabbit anti-alpha-actinin 4 (IF 1:200, clone IG-701, #0042-05, ImmunoGlobe); rabbit anti-p57 (IF 1:400, #sc-8298, Santa Cruz); rhodamine-wheat germ agglutinin (IF 1:400, WGA, #RL-1022, Vector); rat anti-HA (WB 1:1000, clone 3F10, #11867423001, Roche); rabbit pan-proteasomal antibody (IP 2 µg, laboratory stock. Prof. E. Krüger, Biochemistry Greifswald); mouse anti-beta-actin (WB 1:10000, clone AC-15, #A5441, Sigma-Aldrich); for the detection of aggresomes PROTEOSTAT® Protein aggregation assay (ENZO, #ENZ-51023-KP050); sheep anti-murine podocyte antibodies (in vivo application, 1:100 cell culture, 225 µl per mouse in experimental MN, laboratory stock, Meyer-Schwesinger et al (2), AG Meyer-Schwesinger); rabbit anti-THSD7A antibodies (in vivo application 180 µl per mouse, laboratory stock, Tomas et al. (3), AG Meyer-Schwesinger); control rabbit-IgG (in vivo application 180 µl per mouse, #I5006, Sigma); control pre-immune sheep IgG (in vivo application 225 µl per mouse, laboratory stock, Meyer-Schwesinger et al (2), AG Meyer-Schwesinger). All secondary antibodies used were either biotinylated, HRP- or fluorescent dye-conjugated affinity purified donkey antibodies (Jackson ImmunoResearch).

Literature

1. Sosna, J., et al. The proteases HtrA2/Omi and UCH-L1 regulate TNF-induced necroptosis. Cell Commun Signal 11, 76 (2013).
2. Meyer-Schwesinger, C., et al. Nephrotic syndrome and subepithelial deposits in a mouse model of immune-mediated anti-podocyte glomerulonephritis. J Immunol 187, 3218-3229 (2011).
3. Tomas, N.M., et al. A Heterologous Model of Thrombospondin Type 1 Domain-Containing 7A-Associated Membranous Nephropathy. J Am Soc Nephrol 28, 3262-3277 (2017).

Validation

We thoroughly describe the protocol for the usage of antibodies in our manuscript in the method section. All antibodies were validated either
1) All UCH-L1 antibodies, and b5i antibodies were knockout validated by us.
2) HA and FLAG antibodies were validated within the experiments by use of positive and negative controls by us.
3) All other antibodies used were either company or publication validated or validated by us using isotype controls.

Eukaryotic cell lines

Policy information about cell lines and Sex and Gender in Research

Cell line source(s)	murine podocytes from Prof. Karl-Hans Endlich, Anatomy, University Greifswald (Schiwek, D., et al. Stable expression of nephrin and localization to cell-cell contacts in novel murine podocyte cell lines. Kidney Int 66, 91-101 (2004).)<br>HEK293T were purchased from Sigma Aldrich (#12022001), Freestyle293-F were purchased from Thermo Fisher (#100044202).
Authentication	Murine podocytes are authenticated based on their expression of podocyte proteins podocin and synaptopodin at the timing

	of experiments.<br>HEK293T and Freestyle293-F cells were not authenticated.
Mycoplasma contamination	Cells were checked every 3 months for mycoplasma contamination. No contamination was present.
Commonly misidentified lines<br>(See ICLAC register)	No commonly misidentified cell lines were used in the study.

Animals and other research organisms

Policy information about studies involving animals; ARRIVE guidelines recommended for reporting animal research, and Sex and Gender in Research

Laboratory animals	Mice used in the study were older than 8 weeks of age and were predominantly analyzed at 10 - 38 weeks of age. Mice were kept at ambient temperature and humidity, had free access to water and standard animal chow standard animal chow (Altromin 1328 P) and were synchronized to a 12h light : 12h dark cycle.<br>UCH-L1Dpod mice were generated by Genoway, fully backcrossed to the C57BL/6 background and housed at the institutional animal facility.<br>The UCH-L1-WT and UCH-L1-I93M mice were generated by us, backcrossed to the C57BL/6 background and housed at the institutional animal facility.<br>For THSD7A-MN, UCH-L1-I93M mice were backcrossed for 3 generations with BALB/c mice and used in a mixed background.<br>Mice were single housed (males) or maintained in groups (females) in the animal facility of the University Medical Center Hamburg-Eppendorf. housing conditions for the mice, describing dark/light cycle, ambient temperature and humidity
Wild animals	Our study did not involve wild animals
Reporting on sex	Male mice were used for the experimental MN studies (anti-podocyte antibodies and THSD7A-antibodies).<br>Male and female mice were used for the naive studies
Field-collected samples	Our study did not involve field-collected samples
Ethics oversight	The animal studies were conformed to the requirements of the German Animal Welfare Act and approvals were obtained from the State Authority of Hamburg (Behörde für Justiz und Verbraucherschutz, Amt für Verbraucherschutz, Lebensmittelsicherheit und Veterinärwesen), Germany.

Note that full information on the approval of the study protocol must also be provided in the manuscript.
